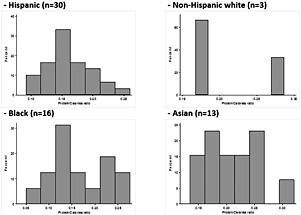# Abstracts of the 9^th^ International Conference on Cachexia, Sarcopenia and Muscle Wasting, Berlin, Germany, 10–11 December 2016 (part 1)

**DOI:** 10.1002/jcsm.12164

**Published:** 2016-11-18

**1-01**

**New formulation based on anti‐atrophic peptides and dendrimers for skeletal muscle atrophy treatment**

Johanna Ábrigo ^1,3^, Valeria Márquez‐Miranda^2,5^, Juan C. Rivera^1,3^, Ingrid Araya‐Durán^2^, Javier Aravena^1,3^, Nicolas Pacheco^3^, Fernando D. González‐Nilo^2,4,5^ and Claudio Cabello‐Verrugio^1,3^

^1^ *Laboratory of Biology and Molecular Physiopathology, Universidad Andres Bello;* ^2^ *CBIB, Universidad Andrés Bello;* ^3^ *IMII, Santiago;* ^4^ *Centro Interdisciplinario de Neurociencia de Valparaíso, Universidad de Valparaíso, Valparaíso, Chile;* ^5^ *Fundación Fraunhofer Chile Research, Santiago, Chile*

**Background**: Loss of muscle strength and myofibrillar proteins are key features in skeletal muscle atrophy by disuse. One of the main mechanisms involved is the over‐activation of ubiquitin–proteasome pathway (UPP). Angiotensin‐(1‐7) [Ang‐(1‐7)], a vasoactive peptide with anti‐atrophic activity in skeletal muscle, is rapidly degraded *in vivo* and inefficient as treatment therapy. Many peptide‐delivery strategies have been studied, including direct injection or administration using osmotic pumps. Dendrimers are promising vehicles for the protection and transport of numerous bioactive molecules, being hydroxyl poly(amidoamine) (PAMAM‐OH) suggested as an safe drug carriers without toxic effects during *in vitro* and *in vivo* applications.

**Aim**: The aim of the study is to evaluate the effect of Ang‐(1‐7)/PAMAM‐OH dendrimer complex intraperitoneally (IP) administered in skeletal muscle atrophy induced by disuse.

**Methods**: C57/BL10J mice were IP injected with vehicle (PBS), Ang‐(1‐7), PAMAM‐OH, and Ang‐(1‐7)/PAMAM‐OH, and 24 h after, immobilized in the lower hindlimb for 24 h or 14 days. Gastrocnemius muscle was extracted, and maximal isometric strength was measured by electrophysiological analyses. Histological analyses were made by hematoxylin eosin stain. Muscle fiber cross‐sectional area (CSA) was determined estimating the minimal Feret's diameter of cryosections stained with Wheat Germ Agglutinin (WGA). Myosin Heavy Chain (MHC) levels were determined by western blot. Atrogin‐1 and MuRF‐1 were evaluated by RT‐qPCR.

**Results**: IP administration of Ang‐(1‐7)/PAMAM‐OH complex, but not Ang‐(1‐7) alone, avoided the decrease of muscle strength, diminution of fiber diameter and decrease of MHC protein levels in the skeletal muscle, induced by disuse. Ang‐(1‐7)/PAMAM‐OH also prevented the increment of atrogin‐1 and MuRF‐1 expression.

**Conclusion**: Ang‐(1‐7) IP administrated as complex with PAMAM‐OH dendrimer avoided the atrophic effects in skeletal muscle induced by disuse. Ang‐(1‐7)/PAMAM‐OH complex can be an efficient method in therapy for treatment of skeletal muscle atrophy.

**Funding**: AFM #16670; FONDECYT #1120380, 1161646; IMII #P09‐016‐F; UNAB DI‐741‐15/N; PhD Scholarship CONICYT; F.G.N.; FCR‐CSB 09CEII‐6991, ACT1107, and RED CYTED 214RT0482; CINV.

**1-02**

**Endotoxin‐induced autophagy dependent on Beclin1/Bcl2 complex is decreased by Angiotensin‐(1‐7) in skeletal muscle**

Juan Carlos Rivera ^1,2^, Johanna Abrigo^1,2^, Mario Chiong^3^, Michael Bader^4,5^, Robson A. Santos^5^, Enrique Brandan^6,7^, María José Acuña^6,7^ and Claudio Cabello‐Verrugio^1,2^

^1^ *Laboratorio de Biología y Fisiopatología Molecular, Universidad Andrés Bello;* ^2^ *IMII;* ^3^ *ACCDiS, Universidad de Chile, Santiago, Chile;* ^4^ *Max‐Delbrück‐Center for Molecular Medicine, Berlin‐Buch, Germany;* ^5^ *National Institute in Science and Technology in Nanobiopharmaceutics, UFMG, Belo Horizonte, Brazil;* ^6^ *CARE;* ^7^ *Department of Cell and Molecular Biology, Universidad Católica, Santiago, Chile*

**Background and Aim**: Autophagy is a key mechanism in endotoxin‐induced cachexia by lipopolysaccharide (LPS). The vasoactive peptide of non‐classical axis of renin angiotensin system, Angiotensin‐(1‐7) [Ang‐(1‐7)] and Mas receptor have anti‐atrophic effects in cachexia, however, is still unknown the effect on autophagy and mechanism in cachectic skeletal muscle.

**Methods**: C57BL/6 J (WT) or KO Mas receptor (KO) mice were treated with LPS in absence or presence of Ang‐(1‐7), and we evaluated autophagic proteins. C_2_C_12_ culture cells exposed to LPS and Ang‐(1‐7), and we analyze autophagic and MAPK proteins, and autophagosome formation.

**Results**: Our results showed that Ang‐(1‐7) decreased the increment of LPS‐induced LC3II/LC3I ratio protein levels in diaphragm muscle compared to control of WT mice. In KO Mas mice, Ang‐(1‐7) lost this effect.

In culture cells exposed to LPS, Ang‐(1‐7) produced a reduction on the autophagy flux, LC3II/LC3I ratio and the amount of autophagosome. Interestingly, LPS increased autophagy by the disruption of the Beclin1/Bcl2 complex, while Ang‐(1‐7) restored its formation. Finally, Ang‐(1‐7) reduced LPS‐activated p38MAPK and JNK, two signalling pathways involved in the separation of the Beclin1/Bcl2 complex in presence of LPS.

**Conclusion**: We suggest that Ang‐(1‐7) is a new regulator of autophagy in endotoxin‐induced cachexia by a mechanism dependent on MAPK and Beclin1/Bcl2 complex.

**Funding**: Association‐Francaise Contre Les Myopathies AFM #16670; FONDECYT #1161646, 1120380; IMII #P09‐016‐F; UNAB DI‐741‐15/N. Conicyt Scholarship #21141242 and #21161353.

**1-03**

**Identification of E2 enzymes involved in MuRF1‐dependent skeletal muscle atrophy**

Cécile Polge, Agnès Claustre, Christiane Deval, Daniel Béchet, Lydie Combaret, Didier Attaix and Daniel Taillandier

*Unité de Nutrition Humaine (UMR 1019 INRA/Clermont Université), CRNH Auvergne63000, Clermont‐Ferrand, France*

**Background and aims**: The Ubiquitin Proteasome System (UPS) is the major actor of muscle wasting during various physio‐pathological situations. This system involves an enzymatic cascade E1, E2, and E3. The targeting specificity of the UPS relies on the capacity of the system to first recognize (E3s) and then label (E2s) the proteins to be degraded generally with a polyubiquitin chain. E2–E3 interactions are therefore crucial as they determine the fate of the substrates. In the past 15 years, numerous experiments have contributed to depict an incomplete picture of the mechanisms responsible for myofibrillar proteolysis. This includes the discovery of muscle‐specific E3 ligases (e.g. MuRF1) and the identification of the signaling pathways involved. Our main objective was to identify the E2‐MuRF1 couples involved in the targeting of myofibrillar proteins in atrophying muscles.

**Methods**: We focused on 14 E2 enzymes that are either abundant in skeletal muscle and/or up‐regulated in atrophying conditions. We used control fa‐C2C12 myotubes (expressing flag‐actin) treated or not with dexamethasone (Dex, 1 μM) and muscles from hindlimb suspensed rats to determine the expression levels of these enzymes. The MuRF1 cognate E2 enzymes were identified by knockdown and interactomic approaches.

**Results**: Dex treatment increased mRNA levels of UBE2A, UBE2B, UBE2D1, UBE2D2, UBE2G1, and UBE2J1. UBE2A did not interact with MuRF1 and was not involved in contractile protein degradation. We next demonstrated that UBE2B was involved in α‐actin and myosin heavy‐chain destabilization in fa‐C2C12 myotubes. However, this process was restricted to the cytoplasmic fraction and was MuRF1‐independent. By contrast with previous assumptions, we found that UBE2D2 is not the MuRF1 partner for α‐actin degradation *in cellulo* and is probably not involved in muscle wasting.

**Conclusions**: UBE2B is the first E2 involved in contractile protein targeting, this action being presumably downstream of MuRF1 action.

**1-04**

**Repetitive pulmonary inflammation in emphysematous mice induces sustained muscle wasting due to impaired muscle mass recovery**

Judith JM Ceelen^1^, Annemie MWJ Schols^1^, Stefan J van Hoof^2^, Chiel C de Theije^1^, Frank Verhaegen^2^ and Ramon CJ Langen ^1^

^1^ *Maastricht University Medical Center, Department of Respiratory Medicine, Maastricht, The Netherlands;* ^2^ *Department of Radiation Oncology (MaastRO)*

**Background and aims**: Exacerbations in COPD are often accompanied by pulmonary inflammation, and associated with increased prevalence of weight loss and muscle wasting. Emphysema‐associated muscle atrophy may result from the cumulative effects of acute muscle mass loss during disease exacerbations, and subsequent impaired muscle regrowth. The aim of this study was to test whether muscle mass recovery following muscle atrophy induced by pulmonary inflammation is impaired in emphysematous mice and culminates in sustained muscle wasting.

**Methods**: Emphysema was induced by 3 weekly intra‐tracheal (IT) elastase instillations. Subsequently, 3 weekly boluses of IT‐LPS were administered to mimic a repetitive pulmonary inflammation‐driven disease exacerbation. Using micro cone‐beam CT‐scans, emphysema was verified, and muscle mass changes were monitored and correlated to muscle strength. At 2 and 3 days following the first IT‐LPS administration and 7 days after the third IT‐LPS bolus, skeletal muscle was collected for analyses.

**Results**: Irrespective of emphysema, muscle weight and strength was reduced 48 h after the first bolus IT‐LPS and recovered thereafter. mRNA and protein levels of genes of the ubiquitin‐proteasome pathway (UPS) and the autophagy‐lysosomal pathway (ALP) were upregulated 48 h following IT‐LPS. In contrast, mTOR signaling was reduced 48 h post‐induction of pulmonary inflammation in control and emphysematous mice. Importantly, muscle strength recovery following subsequent IT‐LPS challenges was impaired in emphysematous mice, resulting in a sustained decrease in muscle mass after 3 successive rounds of pulmonary inflammation.

**Conclusion**: Pulmonary inflammation in emphysematous mice evokes acute loss of muscle mass, mimicking COPD exacerbation‐associated muscle atrophy. Although acute muscle atrophy in response to pulmonary inflammation is not affected by emphysema, muscle mass recovery is impaired by emphysema, resulting in sustained muscle wasting. This suggests that muscle wasting in COPD patients with frequent exacerbations may be a step‐wise process, dictated by phases of loss and impaired recovery of muscle mass.

This study was supported by a grant from the Lung Foundation Netherlands (3.2.11.036).

**1-05**

**Parallel activation of autophagy and myogenic signalling during muscle mass recovery following disuse‐atrophy**

Anita A.E.M. Kneppers ^1^, Nicholas A.M. Pansters^1^, Pieter A. Leermakers^1^, Lex B. Verdijk^2^, Luc J.C. van Loon^2^, Annemie M.W.J. Schols^1^ and Ramon C.J. Langen^1^

^1^ *Department of Respiratory MedicineNUTRIM School of Nutrition and Translational Research in Metabolism, Maastricht University Medical Centre+, Maastricht, The Netherlands;* ^2^ *Department of Human Biology and Movement SciencesNUTRIM School of Nutrition and Translational Research in Metabolism, Maastricht University Medical Centre+, Maastricht, The Netherlands*

**Background and aim**: Muscle wasting is a multifactorial disorder associated with chronic diseases such as chronic obstructive pulmonary disease (COPD). Therapeutic interventions aimed at maintenance and restoration of muscle mass are only in part effective, and further elucidation of the molecular and cellular responses during recovery of muscle mass is required. To better understand the muscle regenerative response during muscle recovery, we studied muscle remodelling‐related processes in a mouse model of reversible skeletal muscle disuse‐atrophy.

**Methods**: Male C57/Bl6 mice were subjected to 14 days of hind limb suspension (HS) followed by 5 days of reloading (RL). Mice were sacrificed at baseline, after HS, and after 1, 2, 3, and 5 days of RL. *M*. *Gastrocnemius* was excised and used for mRNA and protein assessment of markers of autophagy and myogenesis.

**Results**: At 14 days of hind limb suspension, expression of autophagy and myogenesis markers did not differ from baseline. Upon reloading, however, an acute (day 1) increase in ULK1 inhibitory and activating phosphorylation, as well as an increase in LC3B mRNA expression, and P62 mRNA and protein expression occurred. Moreover, LC3BII/I ratio acutely decreased upon reloading. Concomitantly, mRNA expression of M‐cadherin, Myogenin and MyoD were increased at 1 day of reloading. These myogenic differentiation markers, as well as the autophagy markers, were returned to baseline at 3 days of reloading, whereas mRNA expression of the myoblast fusion marker Myomaker was increased on reloading day 5.

**Conclusion**: The parallel changes in expression patterns of autophagy and myogenic differentiation markers during early muscle reloading imply a coordinated regulation of these processes during recovery of muscle mass following atrophy.

**1-06**

**Mobilee®: New Option for Prevention and Treatment of Muscle Atrophy**

Anna Torrent ^1^, Eulàlia Montell^1^, Josep Vergés^1^, Kristy Swiderski^2^, Jarrod E. Church^2^, Dale M. Baum^2^ and Gordon S. Lynch^2^

^1^ *Pre‐Clinical R&D Area, Pharmascience Division, Bioibérica S.A., Barcelona, Spain;* ^2^ *Basic and Clinical Myology LaboratoryDepartment of Physiology, The University of Melbourne, Australia*

**Introduction**/**Aim**: Disuse atrophy might occur from an injury that forces the individual to keep an area in a cast for a prolonged period and in situations in which bed rest or non‐weight‐bearing is mandated for rehabilitation from an injury. Aging is also associated with a progressive decline of muscle mass, quality, and strength (sarcopenia). Any deterioration in the properties of skeletal muscle has an extremely important effect on human health; therefore, there is an acute need to develop novel therapeutic strategies. The aim of this study was to investigate the therapeutic potential of Mobilee®, a rooster comb natural ingredient containing hyaluronic acid, other polysaccharides and collagen, to combat muscle atrophy.

**Methods**: An assay was performed to evaluate the potential to stimulate proliferation of murine C2C12 muscle cells. Two different conditions were evaluated: cells cultured with growth media (10% FBS) and cells cultured with low‐serum media (2%FBS). In addition, the effect on myoblast proliferation in the presence of the cytokine IL‐6 was also tested. In another experiment, we evaluated whether Mobilee® could prevent muscle atrophy (i.e. myotube thickness) during serum starvation conditions.

**Results**: This preparation exerted little effect on cell proliferation in 10% FBS growth serum, but it significantly stimulated proliferation in low‐serum media after 48 hours treatment (131% increase). IL‐6 produced a reduction of myoblast proliferation, and this effect was counteracted by the presence of Mobilee®. In addition, treatment with this extract was able to counteract myotube atrophy compared with the 0% control, as evident from a 20% reduction in myotube width.

**Conclusions**: The results indicate that Mobilee® has potential anabolic effects that could promote myogenesis and anti‐catabolic effects on muscle that could counteract atrophy under adverse conditions. Therefore, Mobilee® may have therapeutic potential for preventing atrophy in muscle wasting conditions such as disuse atrophy, sarcopenia, and other disorders.

**1-07**

**Regulation of skeletal muscle mass by the Vitamin D receptor**

Joseph J. Bass ^1^, Abid A. Kazi^2^, Colleen S. Deane^3^, Asif Nakhuda^1^, Daniel J. Wilkinson^1^, Bethan E. Phillips^1^, Kenneth Smith^1^, Daniel Craig^3^, Andrew Philp^3^, Janelle Tarum^4^, Fawzi Kadi^4^, Nathaniel J. Szewczyk^1^, Mark E. Cleasby^5^ and Philip J. Atherton^1^

^1^ *MRC/ARUK Centre of Excellence for Musculoskeletal Ageing ResearchSchool of Medicine, University of Nottingham, UK;* ^2^ *Department of Cellular and Molecular Physiology, Pennsylvania State University College of Medicine, Hershey, Pennsylvania, USA;* ^3^ *School of SportExercise and Rehabilitation Sciences, University of Birmingham, UK;* ^4^ *School of Health Sciences, Örebro University, Sweden;* ^5^ *Molecular Physiology of Diabetes LaboratoryDepartment of Comparative Biomedical Sciences, Royal Veterinary College, UK*

Vitamin D (VitD) deficiency is present in ~25% of the world's population and has been associated with numerous conditions involving skeletal muscle. Moreover, multiple epidemiological studies have linked VitD deficiency to age‐related sarcopenia (1). VitD supplementation has been shown to enhance muscle function/fibre cross‐sectional area (CSA) in the elderly (2,3). VitD acts through and regulates the Vitamin D receptor (VDR), with expression linked to muscle regeneration (4). However the mechanistic links between VDR and muscle metabolism are poorly defined.

We initially examined the role of the VDR in the myogenic C2C12 cell line by knocking down the VDR (VDR‐KD). VDR‐KD impaired myogenesis as evidenced by blunted proliferation (−27 ± 6%, *P* < 0.01) and DNA synthesis (−31 ± 7%, *P* < 0.05). Moreover, VDR‐KD also impaired differentiation, blunting myosin expression (−89 ± 2%; *P* < 0.001) and rates of myofibrillar protein synthesis (−40 ± 4%; *P* < 0.0001). Next, using *in vivo* shRNA in rat hind limbs, we demonstrated that VDR‐KD muscles underwent fibre atrophy (~8 ± 2%, *P* < 0.001) had reduced protein content (−28 ± 16%, *P* < 0.05) and increased markers of autophagy mediated protein degradation (e.g. LC3B‐II + 84 ± 43%, *P* < 0.05) without impairing anabolic processes. Following this, we over‐expressed the VDR (VDR‐OE), yielding muscle fibre hypertrophy (+17 ± 7%, *P* < 0.05) increased protein content (−28 ± 16%, *P* < 0.05), global protein synthesis (+69 ± 7%, *P* < 0.05), translational efficiency (e.g. mTOR +93% ± 30%, *P* < 0.05), and ribosomal biogenesis (e.g. RPS28 + 79% ± 24%, *P* < 0.05). Finally, to determine the clinical relevance of VDR regulation of muscle mass, we measured VDR expression in humans after 20 weeks of resistance exercise training (RET). Following RET, VDR gene expression increased significantly (+11 ± 4%, *P* < 0.05), tracking with muscle mass increases (Q4 + 20 ± 7%, *P* < 0.05). Interestingly, serum‐VitD (25[OH]D) levels were disconnected from muscle mass changes and VDR expression; however, local expression of VitD metabolizing enzymes increased (CYP27B1 + 121 ± 8%, *P* < 0.01). These data imply a truly mechanistic role of the VDR as an essential aspect of muscle protein metabolism, with genuine implications for maintenance of muscle health.

1. Cangussu LM, Nahas‐Neto J, Orsatti CL, Bueloni‐Dias FN, Nahas E a. P. Effect of vitamin D supplementation alone on muscle function in postmenopausal women: a randomized, double‐blind, placebo‐controlled clinical trial. Osteoporos Int. 2015;

2. Ceglia L, Niramitmahapanya S, Price LL, Harris SS, Fielding R a, Dawson‐Hughes B. An evaluation of the reliability of muscle fiber cross‐sectional area and fiber number measurements in rat skeletal muscle. Biol Proced Online. Biological Procedures Online; 2013;15(1):6.

3. Sato Y, Iwamoto J, Kanoko T, Satoh K. Low‐dose vitamin D prevents muscular atrophy and reduces falls and hip fractures in women after stroke: a randomized controlled trial. Cerebrovasc Dis. 2005 Jan;20(3):187–92.

4. Srikuea R, Zhang X, Park‐Sarge O‐K, Esser K a. VDR and CYP27B1 are expressed in C2C12 cells and regenerating skeletal muscle: potential role in suppression of myoblast proliferation. Am J Physiol Cell Physiol. 2012 Aug;303(4):C396–405.

**1-08**

**New insights into muscle atrophy: role of autophagy in a model of chronic sciatic nerve constriction**

Vincenzo Musolino ^1,2,3^, Francesca Bosco^1,2^, Saverio Nucera^1,2,3^, Luigino Antonio Giancotti^1,2,3^, Sara Ilari^1,2,3^, Filomena Lauro^1,2,3^, Cristina Carresi^1,2,3^, Micaela Gliozzi^1,2,3^, Carolina Muscoli^1,2,3^ and Vincenzo Mollace^1,2,3^

^1^ *Institute of Research for Food Safety & Health (IRC‐FSH), University of Catanzaro “Magna Graecia”, Catanzaro, Italy;* ^2^ *NUTRAMED S.c.a.r.l., Roccelletta di Borgia, Catanzaro, Italy;* ^3^ *IRCCS San Raffaele Pisana, Rome, Italy*

**Background**: Chronic constriction injury (CCI) of the rat sciatic nerve is a widely used model for research of neuropathic pain, but little is known about musculoskeletal changes associated with this injury. Here, we investigated modulation of anabolic and catabolic pathways, focusing on autophagy, in skeletal muscle, using a model of atrophy induced by chronic sciatic nerve constriction.

**Methods**: Rats were divided into CCI and naive groups. In CCI group, sciatic nerve of right hind limb (ipsilateral) was exposed and constricted. Left hind limb (controlateral) underwent no surgical procedures. The naive group rats did not undergo surgery. Body weight and composition were measured before the injury and after 14 days then rats were anesthetized, the ipsilateral and controlateral skeletal muscles dissected and weighed. Anabolic/catabolic signaling was assessed in gastrocnemius muscle (GC).

**Results**: At 14 days post‐sciatic nerve ligation, the CCI group had significant decreases in body weight and body composition compared to naive animals. Muscle weight of the injured hind limb decreased compared to the contralateral side in CCI group. In the ipsilateral GC of the CCI group, western blotting showed an up regulation of the catabolic regulators: Beclin‐1, p62, TRAF6 and LC3, whereas atrogin‐1 was down regulated. Moreover, Pax‐7, p‐Akt and total Akt were up regulated.

**Conclusions**: CCI led to atrophy driven by an increase in autophagic markers. The ubiquitin E3‐ligases atrogin‐1 is decreased, suggesting that its involvement could occur at an earlier time point. Higher Pax7 expression in injured GC was observed, indicating that sciatic nerve damage associates with an expansion of satellite cells in atrophic muscle. Levels of p‐Akt and total Akt were up regulated, a result expected in a hypertrophic model. Although, further experiments are needed to clarify this observation, muscle atrophy induced by chronic sciatic nerve constriction might have different mechanisms than common muscle atrophic models.

**1-09**

**A metabolomics investigation of the impact of a natural myostatin reducing agent derived from fertile egg yolk on humans: a feasibility study**

Neerav D. Padliya ^1^, Laura Shelton^2^, Alex Buko^2^, Maghsoud Dariani^1^ and Robert J. Hariri^1^

^1^ *MYOS Corporation, Cedar Knolls, NJ, USA;* ^2^ *Human Metabolome Technologies America, Inc, Boston, MA, USA*

**Background and aims**: We investigated the feasibility of applying metabolomic profiling to distinguish between subjects that gained varying degrees of muscle mass while participating in a double blind, placebo‐controlled human clinical study to evaluate the impact of the egg yolk‐based dietary supplement, Fortetropin® on skeletal muscle growth over 12 weeks. Supplementation with Fortetropin® lead to increased muscle thickness relative to the placebo macronutrient matched control group (p ≤ 0.05) (Sharp *et*. *al*. *J*. *Am*. *Coll*. *Nutr*. **2016**, in press).

**Methods**: From the 37 subjects that participated in the clinical study, 4 representative subjects were selected for a feasibility study. Subjects A and B exhibited the greatest increase and decrease in muscle thickness, respectively, while undergoing Fortetropin® supplementation over 12 weeks. Subjects C and D exhibited the greatest increase and decrease in muscle thickness, respectively, while receiving a macronutrient matched placebo over 12 weeks.

**Results**: When comparing the subjects that exhibited the greatest increases in muscle thickness over 12 weeks, a significant decrease was found in the lactate/pyruvate ratio for the subject that received Fortetropin® supplementation while a significant increase was found for the subject that received the macronutrient matched placebo, suggesting increased glycolytic activity with Fortetropin®. A further comparison of the subjects that exhibited the greatest increases in muscle thickness over 12 weeks revealed decreased levels of branched chain amino acids (BCAAs) in the subject that received Fortetropin® supplementation, while increased levels of BCAAs were observed in the subject that received the macronutrient matched placebo. Depletion of circulating BCAAs relative to placebo could suggest active muscle protein synthesis, while an increase in BCAAs would suggest protein degradation.

**Conclusion**: In conclusion, metabolomic profiling has shown feasibility to provide insights in the mechanism of action of Fortetropin®.

**1-10**

**Dietary protein intake and protein sources and their associations with selected muscle and physical function measures in older Chinese adults with sarcopenia: preliminary findings**

Ruth Chan^1,2^, Liu‐Ying Zhu^1^, Suey Yeung^1^, Liz Li^1^, Timothy Kwok^1^ and Jean Woo ^1,2^

^1^ *Department of Medicine and Therapeutics, The Chinese University of Hong Kong, Hong Kong SAR;* ^2^ *Centre for Nutritional Studies, The Chinese University of Hong Kong, Hong Kong SAR*

**Background and aims**: Protein intake is a major risk factor of sarcopenia. We aimed to assess the dietary protein intake and sources and their associations with muscle and physical function measures in older Chinese adults with sarcopenia.

**Methods**: Baseline data of 62 Chinese sarcopenic adults aged >65 of an ongoing trial of assessing the role of exercise and nutrition in sarcopenia were analyzed. Sarcopenia was defined using the Asian Working Group Criteria. Dietary data were assessed using a 3‐day diet record. Physical activity was measured using the Physical Activity Scale for the Elderly (PASE). Measurements included body composition, 6‐meter usual pace walk, seated medicine ball throw, 5‐chair stands, 6‐minute walk test, leg extension and SF‐12. Linear regression analyses were used to examine the age‐ and sex‐adjusted association of dietary protein intake and sources with various outcomes.

**Results**: Relative protein intake averaged 1.6 ± 0.5 g/kg‐bw/day. Nine (14.5%) participants showed a protein intake below 1.2 g/kg‐bw/day. Animal sources and plant sources contributed to 60% and 40%, respectively, of the total protein intake. Dietary protein intake averaged 12.7 ± 8.3 g/day at breakfast, 21.4 ± 10.5 g/day at lunch and 30.4 ± 12.3 g/day at dinner. Animal protein contributed to 48.5%, 56.0% and 65.6% of protein intake at breakfast, lunch and dinner, respectively. There was no association between total protein intake and outcome measures. Animal protein intake was inversely associated with seated medicine ball throw, 6‐minute walk test, and leg extension. Plant protein intake was positively associated with SF‐12. Regression analyses showed PASE as a more significant factor to various outcome measures compared to protein intake and sources.

**Conclusions**: We found a minimal association of protein intake and sources with muscle and physical function measures in older Chinese adults with sarcopenia, possibly due to the replete protein intake of our sample.

**1-11**

**Decreased risk of sarcopenia with higher blood levels of leucine, essential amino acids, EPA and 25‐hydroxyvitamin D**

Sovianne ter Borg ^1^, Yvette C. Luiking^1,2^, Sjors Verlaan^1^, Yves Boirie^3,4,5,6^, Jos M.G.A. Schols^7^ and Lisette C.P.G.M. de Groot^7^

^1^ *Nutricia Research, Nutricia Advanced Medical Nutrition, Utrecht, The Netherlands;* ^2^ *Center for Translational Research in Aging and LongevityDepartment of Health and Kinesiology, Texas A&M University, USA;* ^3^ *University of Clermont Auvergne, Unité de Nutrition Humaine, Clermont‐Ferrand, France;* ^4^ *INRA, UMR 1019, UNH, CRNH Auvergne, Clermont‐Ferrand, France;* ^5^ *CHU Clermont‐Ferrand, Clinical Nutrition Department, Clermont‐Ferrand, France;* ^6^ *Department of Health Services Research and Department of Family MedicineSchool CAPHRI, Maastricht University, Maastricht, The Netherlands;* ^7^ *Wageningen University, Division of Human Nutrition, Wageningen, The Netherlands*

**Background**: Nutrition is seen as an important pillar in sarcopenia prevention. Our objective was to investigate the relation between biochemical nutrient markers and the risk of sarcopenia.

**Methods**: Blood samples were collected from 226 community‐dwelling older adults (≥65 years), who participated in the Maastricht Sarcopenia Study. Postabsorptive serum amino acids, 25‐hydroxyvitamin D (25OHD), magnesium, α‐tocopherol/cholesterol ratio, red blood cell (RBC) fatty acid composition and plasma homocysteine were assessed. Sarcopenia was identified in 23%, according the European Working Group on Sarcopenia in Older People definition. Blood biochemical nutrient level quartiles (Q) were examined for their association with sarcopenia by logistic regression analysis. The covariates age, sex and BMI were included. Data are presented as odds ratio (OR) with [95% confidence interval].

**Results**: Participants in Q4 of leucine, branched chain amino acids (BCAA), and essential amino acids (EAA) had a lower risk of having sarcopenia compared to those in Q1 (OR 0.27 [0.10–0.75]; OR 0.23 [0.08–0.64]; OR 0.28 [0.11–0.73], respectively). Similarly, participants in Q4 of EPA and 25OHD had a lower risk of sarcopenia compared to those in Q1 (OR 0.33 [0.12–0.87]; OR 0.33 [0.13–0.84], respectively). Those in Q4 of homocysteine had a higher risk of sarcopenia (OR 2.74 [1.11–6.75]), compared to those in Q1. No significant associations with sarcopenia were observed for total amino acids (OR 0.42 [0.16–1.07]), magnesium (OR 0.96 [0.41–2.24]), α‐tocopherol/cholesterol ratio (OR 1.33 [0.53–3.36]) and other RBC fatty acids. Age was identified as a possible confounder of the observed ORs.

**Conclusions**: High concentrations of leucine, BCAA, EAA, EPA and 25OHD are associated with a lower risk of having sarcopenia. High concentration of homocysteine is associated with a higher risk of sarcopenia. This suggests that diet quality, i.e. quality of the protein and fat source and adequate micronutrients, may be relevant in the prevention of sarcopenia.

**1-12**

**Heart failure induces extensive alterations of the skeletal muscle gene expression program**

Lucia M. Leitner ^1^, Katharina Bottermann^1^, Mirjam Pfeffer^1^, Jana Nemmer^1^, René Deenen^2^, Karl Köhrer^2^, Johannes Stegbauer^3^ and Axel Gödecke^1^

^1^ *Heinrich‐Heine‐University, Cardiovascular Physiology, Düsseldorf, Germany;* ^2^ *Heinrich‐Heine‐University, BMFZ, Düsseldorf, Germany;* ^3^ *Heinrich‐Heine‐University, Nephrology, Düsseldorf, Germany*

**Background**: Angiotensin II induces a severe heart failure (HF) phenotype in cardiomyocyte (CM) specific KO of p38α MAP kinase within 2 days characterized by LV dilatation and reduced ejection fraction (EF). We used this model to study the extent of crosstalk between the failing heart and skeletal muscle (SkM).

**Methods**: p38 Map kinase was inactivated in CM of adult mice, and HF was induced by Angiotensin II (1.5 mg/kg/day) applied via osmotic mini pumps for 2 days. Cardiac function was assessed by high resolution ultrasound. Gene expression in heart and SkM (*M*. *plantaris*) was analyzed by microarrays (Agilent 8x60K Mouse Array) and quantitative RT‐PCR.

**Results**: Pressure overload induced a reduced EF and LV dilatation in KO mice within 2 days. Microarray analysis uncovered more than 4000 (hearts) and 1300 (*M*. *plantaris*) differentially expressed genes (>3‐fold) in AngII treated KO mice when compared to AngII treated controls. Both tissues showed increased levels of cytokine expression (e.g. IL‐6, IL‐1b, IL‐6r, and IL‐1r2), additionally several deregulated atrogenes were found in *M*. *plantaris* (including FoxO1, FoxO3, MuRF1, and Atrogin1), indicating the start of a wasting related gene program, triggered by HF. A possible heart‐skeletal muscle crosstalk was further reinforced by the time course (12, 24, and 48 h after AngII treatment) of gene expression, which showed first changes in heart after 24 hrs followed by SkM changes after 48 hrs. Granulocyte depletion as a potential intervention partially rescued the cardiac and SkM phenotype.

**Conclusions**: The high number of deregulated genes in the skeletal muscle of CM specific p38 KO mice after 2 days of AngII treatment are most likely triggered by the failing heart. The severity of the HF phenotype clearly correlates with SkM changes. Cytokine secretion seems to be an early mediator of HF induced muscle wasting.

**1-13**

**Accelerated grip strength decline in older men with poor health and hormonal deficits – the prospective STRAMBO study**

Pawel Szulc and Roland Chapurlat

*INSERM UMR 1033, University of Lyon, Department of Rheumatology and Bone Pathology, Hôpital Edouard Herriot, Pavillon F, Place d'Arsonval69437, Lyon, France*

**Background**: Ageing‐related health deterioration may contribute to the aggravation of dynapenia.

**Aim**: To assess the determinants of the prospectively assessed grip strength decline.

**Methods**: In 756 men aged ≥60 followed up for 8 years, grip strength was measured every 4 years (Martin Vigorimeter dynamometer). Physical performance was assessed using a composite score (chair stands and balance). The analyses were adjusted for confounders including age, weight, lifestyle, treatments, and other investigated conditions described below.

**Results**: Average grip strength decline was −1.09 kPa/year (p < 0.001). Strength decline was more rapid in men with insulin‐treated diabetes mellitus vs. men without diabetes (difference between the groups: −6.08 kPa/year; 95%CI: −11.41, −0.75; p < 0.05). Prior myocardial infarction and Parkinson disease were each associated with more rapid strength decline (−3.48 kPa/year, p < 0.05, and −6.53 kPa/year, p < 0.05, respectively, vs. men without these characteristics). Men with vertebral fractures has more rapid strength decline vs. men without vertebral fractures (−3.18 kPa/year; p < 0.01).

Men self‐reporting poor health had more rapid strength decline vs. men with excellent/good/fair health (−17.75 kPa/year; 95%CI: −25.02, −10.47; p < 0.001). Poor physical performance (lowest tertile) was associated with more rapid strength decline (−7.14 kPa/year; 95% CI: −8.73, −5.56; p < 0.001).

Men with free testosterone levels <150pmol/L had more rapid strength decline vs. men with higher levels (−2.85 kPa/year; 95%CI: −5.01, −0.69; p < 0.01). Men with 25‐hydroxycholecalciferol (25OHD) levels <20 ng/mL had more rapid grip strength decline (−2.49 kPa/year; 95% CI: −4.22, −0.76; p < 0.005) vs. men with 25OHD >30 ng/mL. Men with high‐sensitivity C‐reactive protein (hs‐CRP) levels >3 ng/mL had more rapid grip strength decline (−3.15 kPa/year; 95% CI: −4.90, −1.40; p < 0.001) vs. men with hs‐CRP <1 ng/mL.

**Conclusion**: In older men poor health status and hormonal deficits are associated with the accelerated grip strength decline regardless of potential confounders. These factors should be included in the assessment of dynapenia in the clinical practice.

**1-14**

**Efficacy of Mobilee on muscle function in patients with joint discomfort: A subject‐level meta‐analysis**

Anna Torrent ^1^, Daniel Martínez‐Puig^1^, David Moriña^2^, Rosa M. Valls^3^, Anna Pedret^3^, Monica Giralt^3^ and Rosa Solà^3^

^1^ *Bioiberica, S.A., Palafolls, Spain;* ^2^ *Technological Center of Nutrition and Health (CTNS) ‐TECNIO ‐URV ‐CEICS, Reus, Spain;* ^3^ *Universitat Rovira i Virgili, Reus, Spain*

**Background and aims**: A rooster comb extract containing hyaluronic acid (HA) polysaccharides and collagen (Mobilee™) is proposed to strength muscles in mild knee pain patients. The aim of this meta‐analysis was to determine the effects of daily consumption of a low‐fat dairy product supplemented with Mobilee™ (80 mg/day) on muscle function, echographic evolution, and knee discomfort using a Visual‐Analogue‐Scale (VAS) compared with a low‐fat dairy product consumed by 3 months on affected knee.

**Methods**: The individual data from 148 volunteers of Intention‐To‐Treat population (ITT) (51 men; aged from 20 to 75 y) of two randomized, controlled, double‐blind, parallel trials (68 volunteers, Sanchez et al 2014; and 80 volunteers, Solà et al 2015) performed on patients with mild knee pain (VAS between 30 and 50 mm) developed in Barcelona and Reus (Spain) by implementing the same protocol. The muscle function determined by peak torque, total work, and power mean in flexion and extension at speeds of 180°/s and 240°/s using an isokinetic dynamometer Biodex4. The outcomes evaluation was performed by an ANCOVA model with the baseline value as covariate without missing data imputation on the ITT population.

**Results**: After 3 months, compared to control, improved total work in flexion at 180°/s (P = 0.039), reduced synovial effusion (P = 0.037), and perception of pain diminished (P = 0.003) on affected knee particularly in men older than 50.

**Conclusions**: Long‐term low‐fat dairy product supplemented with HA consumption improves muscle strength, synovial effusion, and pain providing clinical benefit, especially in men 50 years and older on mild knee pain patients.

**1-15**

**The effects of cigarette smoke on catabolism of skeletal muscle—implications to sarcopenia**

Oren Rom^1^, Dror Aizenbud^1,2^ and Abraham Z. Reznick ^1^

^1^ *Rappaport Faculty of Medicine, Technion—Israel Institute of Technology, Haifa, Israel;* ^2^ *Orthodontic and Craniofacial Department, Rambam Health Care Campus, Haifa, Israel*

Cigarette smoking has been identified as a risk factor for sarcopenia, the age‐related loss of skeletal muscle mass and strength. This study aimed to investigate the mechanisms by which cigarette smoke (CS) induces muscle catabolism and to identify components of CS that may be responsible. Also, this study aimed to investigate the effects of smoking versus smoking cessation on muscle mass, strength, and body composition (BC).

Skeletal myotubes differentiated from the C2 myoblast cell‐line were exposed to CS and CS components that have been suggested to damage skeletal muscle – the aldehydes acetaldehyde and acrolein and the reactive nitrogen species peroxynitrite. Their effects on oxidative stress, p38 MAPK, and NF‐kB pathways, the ubiquitin‐proteasome system and breakdown of muscle proteins were studied using microscopy, western blotting, and qPCR.

A clinical study of 81 adult smokers recruited from the smoking cessation program of Clalit Health Services was conducted. Measurements were held at the beginning of the program and after 12 months. BC was assessed by dual‐energy X‐ray absorptiometry and bioelectrical‐impedance analysis. Muscle strength was measured by handgrip dynamometry and one‐repetition maximum tests. Dietary intake and physical activity were estimated by questionnaires, and smoking status was determined by urine cotinine levels. Linear regression models were used to assess the effect of smoking versus smoking cessation.

Exposure of myotubes to CS caused increased oxidative stress and activation of the p38 MAPK and NF‐kB pathways which led to the up‐regulation of MAFbx/atrogin‐1 and MuRF1. CS caused a time‐ and dose‐dependent degradation of myosin heavy chain and reduction of myotube diameter. Pretreatment with N‐acetylcysteine, essential amino acid leucine, and inhibitors of p38 MAPK, NF‐kB, and the proteasome abolished the effects of CS. Exposure of myotubes to acrolein and peroxynitrite but not to acetaldehyde activated a similar catabolic pathway as CS exposure.

Forty‐one participants completed all measurements (76% smokers; 24% quitters). Adjusting for dietary intake and physical activity changes, significant increases in body weight, muscle mass, fat mass, bone mineral density, and muscle strength were found in quitters compared with smokers.

The *in vitro* study provided a cellular mechanism for the deleterious effects of CS on skeletal muscle and suggested that acrolein and peroxynitrite but not acetaldehyde may be responsible for CS‐induced muscle catabolism. The clinical study demonstrated that smoking cessation is associated with increased muscle mass, strength, and bone density compared with smoking continuation. Therefore, smoking cessation may be a possible strategy to delay or prevent sarcopenia.

**1-16**

**What is the role for beta‐aminoisobutyric acid (BAIBA) in physical activity and its barriers among patients on chronic hemodialysis (HD)?**

Alessio Molfino ^1^, Maria Ida Amabile^1^, Maria Grazia Chiappini^2^, Luana Lionetto^3^, Alessio Farcomeni^4^, Maurizio Simmaco^5^, Thomas Ammann^2^, Filippo Rossi Fanelli^1^, Alessandro Laviano^1^ and Maurizio Muscaritoli^1^

^1^ *Department of Clinical Medicine, Sapienza University of Rome, Rome, Italy;* ^2^ *Hemodialysis Unit, Fatebenefratelli Isola Tiberina Hospital, Rome, Italy;* ^3^ *Personalized Medicine Unit, Istituto Dermopatico dell'Immacolata‐IRCCS, Rome, Italy;* ^4^ *Department of Public Health and Infectious Diseases, Sapienza University of Rome, Italy;* ^5^ *Neuroscience, Mental Health and Sense Organs (NESMOS) DepartmentAdvanced Molecular Diagnostics Unit, Sant'Andrea Hospital, Rome, Italy*

**Background and aims**: Physical inactivity is frequently found in HD patients with frailty, and it represents a predictor of morbidity and mortality. Barriers to physical activity have been identified. BAIBA is a contraction‐induced myokine, whose plasma levels increase with exercise and are inversely associated with metabolic risk factors. Aim is to ascertain whether physical inactivity and clinical parameters relate to plasma BAIBA levels in HD patients.

**Methods**: Adult HD patients were included. Physical activity was assessed by questionnaire. BAIBA levels were measured by mass spectrometry in patients and controls. Barriers to physical activity were assessed investigating 23 items concerning psychological, physical, financial, and social barriers. Body composition was evaluated by body impedance analysis and muscle strength by hand grip dynamometer. Non‐parametric tests and logistic regression analyses were performed to study the association between barriers to physical activity, participation to physical activity, BAIBA levels, and clinical parameters.

**Results**: Forty‐nine HD patients were studied, 51% resulted inactive patients. Forty‐three patients showed barriers to physical activity, and 61% of inactive patients reported 3 or more barriers. BAIBA levels were significantly lower in HD patients respect to healthy subjects (P < 0.001). Stratifying patients as active and inactive, both groups showed significantly lower BAIBA levels vs controls (P = 0.0005, P < 0.001, respectively). Non‐diabetic patients showed increased BAIBA levels with respect to diabetic patients (0.43, IQR:0.26–0.85 vs 0.32, IQR:0.14–0.43) (P < 0.001). Patients endorsing the two most frequent barriers showed lower BAIBA levels vs those not reporting these barriers (0.26, 95% CI:0.12–0.43 vs 0.47, 95% CI:0.29–0.85, respectively; P = 0.006). Active patients showed higher intracellular water (P = 0.008), and active and inactive patients showed significant correlation between total body muscle mass and hand grip (P = 0.04, P = 0.005, respectively).

**Conclusions**: Physical inactivity is highly prevalent among HD patients, and BAIBA correlates with the two most common endorsed barriers to physical activity by inactive patients.

**1-17**

**Musculoskeletal system and cognitive function in an Iranian population: Bushehr Elderly Health (BEH) program (Stage II)**

Gita Shafiee ^1^, Ramin Heshmat*^1^, Afshin Ostovar^2^, Hossein Darabi^2^, Farshad Sharifi^3^, Alireza Raeisi^2^, Bagher Larijani^4^ and Iraj Nabipour^5^

^1^ *Chronic Diseases Research CenterEndocrinology and Metabolism Population Sciences Institute, Tehran University of Medical Sciences, Tehran, Iran;* ^2^ *The Persian Gulf Tropical Medicine Research Center, Bushehr University of Medical Sciences, Bushehr, Iran;* ^3^ *Elderly Health Research CenterEndocrinology and Metabolism Population Sciences Institute, Tehran University of Medical Sciences, Tehran, Iran;* ^4^ *Endocrinology and Metabolism Research CenterEndocrinology and Metabolism Clinical Sciences Institute, Tehran University of Medical Sciences, Tehran, Iran;* ^5^ *The Persian Gulf Marine Biotechnology Research CenterThe Persian Gulf Biomedical Sciences Research Institute, Bushehr University of Medical Sciences, Bushehr, Iran*

**Introduction**: The world population is aging at an unprecedented rate, and health of elderly has become one of the main priorities of public health systems. Musculoskeletal and cognitive diseases are prevalent, and they are significant determinants of morbidity and mortality in older adults. Osteoporosis should be considered as the most common bone diseases that may lead to an increase risk of fractures. Sarcopenia, the age‐related decline in muscle mass and function, is a major risk factor of falling, functional limitation, and disability in the elderly.

Given the importance of geriatric diseases, the aim of this study is to investigate the prevalence of musculoskeletal and cognitive diseases and their risk factors and to assess their associations during future follow ups. Also, we will investigate a number of possible molecular mechanisms linking to geriatric diseases.

**Methods**: Bushehr Elderly Health (BEH) program is a population‐based prospective cohort study which is being conducted in a southern province of Iran. The second stage has been begun from October 2015, and 3000 subjects aged ≥60 years from stage I will be re‐invited. Data including demographic status, lifestyle factors, general health, medical history, mental‐functional health, and medication use are collected through a questionnaire.

Anthropometric data, handgrip strength, usual gait speed (physical performance), and the Short Physical Performance Battery are measured. Body composition and bone mineral density of the lumbar spine and total hip are measured using DXA. Mental and functional health assessments are performed by using standard questionnaires. A total blood is taken, and sera are stored for future analyses. By July 2016, data of 950 subjects are collected.

**Conclusion**: This study protocol focuses on the epidemiological characteristics of geriatric disorders, in addition to elucidate biological risk factors of musculoskeletal and cognitive diseases, and assessment of associations during future follow ups. The follow‐up assessments will be carried out every 5 years for three consecutive periods. The findings will not only improve our understanding of disease prevalence in the elderly, but also have the potential to inform the development of beneficial interventions to improve the management of musculoskeletal and cognitive diseases in Iran and other countries in the Middle East.

**1-18**

**Can intradialytic exercise increase daily physical activity in maintenance hemodialysis patients?**

Jun Chul Kim and Ji‐Hyung Cho

*Department of Internal MedicineCHA Gumi Medical Center, CHA University, Gumi‐si, Gyeongsangbuk‐do, Korea*

**Background and aims**: Physical inactivity has been reported to relate with poor health‐related quality of life (HRQOL), disability, higher rates of hospitalization, and mortality in dialysis patients. We aimed to investigate the effect of intradialytic exercise (IDE) on daily physical activity (DPA) measured by 3‐dimentional accelerometer in maintenance hemodialysis (MHD) patients.

**Methods**: This study randomly assigned ambulatory MHD patients aged ≥20 years on dialysis ≥6 months, without hospitalization history for the previous 3 months to 4 groups: aerobic (AE), resistance (RE), combination exercise (CE), and control (CG). Stationary bike was used during hemodialysis for AE and TheraBand®/theraball for RE. Twelve‐week IDE program (3 times/week) was completed in AE (n = 11), RE (n = 10), and CE (n = 12). CG (n = 13) received only warm‐up stretching. At baseline and 12‐week follow up, DPA was measured by a 3‐axis accelerometer (wActiSleep‐BT, ActiGraph LLC) during continuous 7‐day wear period.

**Results**: Patients were 55 ± 12 years of age (mean ± SD) on MHD for 64 ± 72 months, 50% female, 44% diabetic. We observed a significant increase of activity‐related MET (Metabolic Equivalent; kcal/h/kg) compared with baseline in AE (1.02 ± 0.03 vs 1.04 ± 0.04, *P* = 0.04) and CE (1.06 ± 0.05 vs 1.09 ± 0.08, *P* = 0.003) at 12‐week. When comparing between‐group changes to MET, there was a significant increase in CE (0.03 ± 0.03 vs −0.01 ± 0.04, *P* = 0.014) compared with CG. The total number of sedentary bouts (per week) decreased significantly in AE (200 ± 37 vs 174 ± 36, *P* = 0.016) and CE (180 ± 45 vs 152 ± 46, *P* = 0.031) at 12‐week compared with baseline.

**Conclusions**: These findings suggest that IDE may play a significant role in the improvement of DPA in MHD patients, although future studies with more study subjects and longer intervention duration are needed to confirm our findings and if this would also lead to improvement of clinical outcomes, such as HRQOL, hospitalization, and mortality.

**1-19**

**Increased myogenic and protein turnover signalling in skeletal muscle of COPD patients with sarcopenia**

Anita A.E.M. Kneppers ^1^, Ramon C.J. Langen^1^, Harry R. Gosker^1^, Lex B. Verdijk^2^, Pieter A. Leermakers^1^, Marco C.J.M. Kelders^1^, Chiel C. de Theije^1^, Nanca Cebron Lipovec^3^, Dane Omersa^4^, Mitja Lainscak^5,6^ and Annemie M.W.J. Schols^1^

^1^ *Department of Respiratory MedicineNUTRIM School of Nutrition and Translational Research in Metabolism, Maastricht University Medical Centre, Maastricht, The Netherlands;* ^2^ *Department of Human Biology and Movement SciencesNUTRIM School of Nutrition and Translational Research in Metabolism, Maastricht University Medical Centre, Maastricht, The Netherlands;* ^3^ *Pharmacy Department, University Clinic of Pulmonary and Allergic Diseases Golnik, Golnik, Slovenia;* ^4^ *Research Department, National Institute of Public Health, Ljubljana, Slovenia;* ^5^ *Department of Cardiology, General Hospital Celje, Celje, Slovenia;* ^6^ *Faculty of Medicine, University of Ljubljana, Ljubljana, Slovenia*

**Background and aims**: Sarcopenia is a frequently observed co‐morbidity of chronic obstructive pulmonary disease (COPD). Although loss of muscle mass is the result of an imbalance in protein turnover, the relative contribution of altered protein synthesis and protein degradation in COPD‐related sarcopenia remains unclear. As alterations in protein turnover are regulated by anabolic and catabolic signalling, we aimed to (1) verify alterations in protein turnover regulation in the skeletal muscle of COPD patients compared to controls and (2) assess differential regulation of protein synthesis and degradation in COPD patients with and without sarcopenia.

**Methods**: Muscle biopsies were obtained from the *M*. *vastus lateralis* of 13 controls (Age 65 ± 5; 54% male) and 92 COPD patients (Age 65 ± 8; 66% male). Patients were clustered based on sarcopenia defined by low appendicular skeletal muscle mass index (53 non‐sarcopenic COPD; 39 sarcopenic COPD). The mRNA and protein expression of regulators (i.e., FOXO1 and FOXO3) and mediators of the ubiquitin proteasome system (UPS) (i.e., MURF1, ATROGIN1, SMART, and MUSA) and the autophagy‐lysosome system (ALP) (i.e., LC3BI/II, P62, BECN1, and ULK1), and protein synthesis (i.e., AKT1, RPS6, and 4E‐BP1) were analysed by RT‐qPCR and western blot. Furthermore, the mRNA expression of the myogenesis markers; PAX7, PCNA, CCND1, MYF5, MYOD1, MYOG, M‐cadherin, and MSTN were analysed.

**Results**: Constituents reflecting UPS signalling seemed unaltered, while ALP signalling was increased in COPD and even further increased in sarcopenic COPD. Similarly, protein synthesis signalling was increased in COPD and even more so in sarcopenic COPD. Furthermore, myogenic signalling was increased in COPD, despite a concomitant increase in MSTN mRNA expression.

**Conclusion**: We confirm increased protein turnover signalling in the skeletal muscle of COPD patients and show a further stimulation of these processes in sarcopenic COPD patients. In combination with the increase in myogenic signalling, these molecular alterations are suggestive of muscle repair and remodelling.

**1-20**

**Skeletal muscle density, but not skeletal muscle mass, is associated with impaired survival in patients with suspected perihilar cholangiocarcinoma and may identify patients at risk for early death**

Jeroen L.A. van Vugt ^1^, Marcia P. Gaspersz^1^, Jaynee Vugts^1^, François E.J.A. Willemssen^2^, Bas Groot Koerkamp^1^ and Jan N.M. IJzermans^1^

^1^ *Department of Surgery, Erasmus University Medical Center, Rotterdam, The Netherlands;* ^2^ *Department of Radiology, Erasmus University Medical Center, Rotterdam, The Netherlands*

**Background**: Low skeletal muscle mass (i.e. sarcopenia) is associated with increased postoperative morbidity and impaired survival following liver resection for perihilar cholangiocarcinoma (PHC). However, the majority of patients does not undergo surgery. The aim of this study was to investigate the association between sarcopenia as biomarker to predict the outcome of patients with suspected PHC, regardless of treatment.

**Methods**: All consecutive patients with suspected PHC treated in a tertiary center between 2002 and 2014 were included. Baseline characteristics and parameters regarding disease (e.g. CA19‐9 and vascular involvement) and treatment were collected and retrospectively analyzed. Skeletal muscle mass and skeletal muscle density, reflecting intramuscular adipose tissue infiltration and muscle quality (in Hounsfield units [HU]), were measured on the level of the third lumbar vertebra (L3) on abdominal computed tomography scans which were performed during the diagnostic work up. Skeletal muscle mass was corrected for patients' height, resulting in the L3 muscle index (cm^2^/m^2^). Cut‐off values for skeletal muscle mass defined by Coelen et al. were used to classify patients as (non)sarcopenic. Overall survival (OS) was compared using the Kaplan Meier method, Cox regression analysis, and log‐rank test.

**Results**: In total, 241 patients with available imaging were included (60.2% men) with a median age of 66 years and BMI of 25 kg/m^2^. The median L3 muscle index was 48.0 cm^2^/m^2^ for men and 38.4 cm^2^/m^2^ for women (p < 0.001), resulting in a sarcopenia prevalence of 46.3%. No significant differences in survival were observed between patients with low compared with those with normal skeletal muscle mass. Using the median skeletal muscle density (35 HU) as cut‐off value, the median survival was 13 versus 6 months in patients with normal compared with patients with low skeletal muscle density (HR 1.42 [95% CI 1.09–1.84], log‐rank p = 0.007). Three‐months and 1‐year OS rates of patients with low skeletal muscle density compared with normal skeletal muscle density were 72.6% versus 89.5% (p = 0.001) and 33.3% versus 54.0% (p = 0.004). No significant differences were observed for 3‐year and 5‐year OS. After correction for age, bilirubin and CA19‐9 level, cholangitis at presentation, and the suspicion of positive lymph nodes or metastases on imaging, skeletal muscle density was not independently associated (adjusted HR 1.19 [95% CI 0.78–1.82], p = 0.418).

**Conclusion**: Sarcopenia is highly prevalent in patients with PHC, but not independently associated with impaired outcome. Nevertheless, low skeletal muscle density may identify patients with PHC at risk for early death.

**1-21**

**The frailty‐based prognostic criteria in heart failure patients. A multicenter prospective cohort study (FLAGSHIP study): Design and preliminary data**

Sumio Yamada ^1^, Hideo Izawa^2^, Toyoaki Murohara^3^, Takaaki Kondo^1^, Takuji Adachi^4^

on behalf of FLAGSHIP study investigators

^1^ *Nagoya University Graduate School of Medicine (Health Sciences), Nagoya, Japan;* ^2^ *Department of Cardiology, Fujita Health University Banbuntane Hotokukai Hospital, Nagoya, Japan;* ^3^ *Department of Cardiology, Nagoya University Graduate School of Medicine, Nagoya, Japan;* ^4^ *Program in Physical and Occupational Therapy, Nagoya University Graduate School of Medicine, Nagoya, Japan*

**Background**: Frailty has been recently well documented as a clinical marker for management of patients with heart failure (HF). However, frailty criteria specific to prognosis of HF have not been established. We therefore started a multicenter prospective cohort study to develop frailty‐based prognostic criteria in HF patients (FLAGSHIP study).

**Methods**: FLAGSHIP study is designed to investigate (1) development of frailty criteria specific to HF patients, (2) associated factors of frailty in HF at the time of discharge, and (3) clinical strategy to manage HF frailty. In‐patients with HF who can walk for 20 m at discharge are eligible for the study. Patients with dementia, mental disorder, short‐term vital prognosis, or difficulty in answering questionnaires are excluded. Each subject receives comprehensive assessment including frailty, nutrition, depression, and clinical state during hospitalization. Frailty is measured using phenotype as follows: weight loss, weak grip strength, slow walking speed, exhaustion, and physical inactivity. Data regarding etiology, medical history, echocardiography, blood tests, and prescription were obtained from medical records. Follow‐up survey is conducted for two years after discharge. Main outcomes of this study include HF readmission, cardiac events, fracture due to fall, and all‐cause death. Frailty criteria will be established based on the HF types (HFrEF or HFpEF).

**Results**: Thirty‐five medical centers have participated in the study, and 30 of them have already enrolled 600 subjects until July 2016. Mean age is 72.1 ± 13.3 and 61.4% are male. The prevalence of HF with preserved ejection fraction (LVEF≧50%) is 47.4%. According to our temporary criteria (Yamada S, et al. ESC Heart Failure 2015), the prevalence of frail HF is 17.2%.

**Conclusion**: FLAGSHIP study will provide the world's first frailty criteria in Asian patients with HF after three years later. The design and latest preliminary data in more will be discussed.

**1-22**

**Role of arteriosclerosis in reduction of skeletal muscle mass in patients with cardiovascular disease**

Haruhito Harada, Yuichi Hattori, Hitoshi Hamamura, Hiroshi Niiyama, Atsushi Katoh and Hisashi Kai

*Department of Cardiology, Kurume University Medical Center, Kurume, Japan*

**Background**: Reduction of skeletal muscle mass is the most important component on diagnosis of sarcopenia. Aging and chronic diseases, including chronic heart failure, are the cause of reduction of skeletal muscle mass. However, little is known about the mechanism of skeletal muscle mass reduction in sarcopenia with cardiovascular disease. The purpose of this study was to assess the relationship of arteriosclerosis to reduction of skeletal muscle mass, because arteriosclerosis is the most common underlying pathogenesis of cardiovascular disease, especially in the elderly.

**Methods**: Study design is a retrospective cross‐sectional analysis. Subjects were 310 in‐patients with cardiovascular disease in our hospital. Flow mediated vasodilatation (FMD), arterial velocity pulse index (AVI), bioelectrical impedance phase angle (PA), and skeletal muscle index (SMI) were assessed, and correlation analyses were performed among these parameters. FMD and AVI are adopted as arteriosclerotic markers. PA and SMI are markers of tissue damage and skeletal muscle volume, respectively. PA and skeletal muscle volume were obtained by bioelectrical impedance assay.

**Results**: Average age of the subjects was 72 ± 12 years old, and proportion of male was 58%. Coronary artery disease, peripheral artery disease, and symptomatic heart failure were found in 47.4%, 4.2%, and 19.4% of the subjects, respectively. The prevalence of hypertension, diabetes mellitus, dyslipidemia, and chronic kidney disease were 62.9%, 36.1%, 40.6%, 14.8%, and 25.5%, respectively. Sarcopenia was diagnosed in 25.5% according to AWGS criteria. FMD weakly correlated with AVI (r = −0.24, p < 0.0001), PA (r = 0.23, p < 0.0001) and SMI (r = 0.17, p < 0.005). AVI negatively correlated with PA (r = −0.40, p < 0.0001) and SMI (r = −0.41, p < 0.0001). Positive correlation was found between PA and SMI (r = 0.49, p < 0.0001).

**Conclusion**: It is suggested that arteriosclerosis would play an important role in the reduction of skeletal muscle mass through the damage of skeletal muscle tissue in patients with cardiovascular disease.

**1-23**

**The impact of muscle depletion on survival in cirrhotic patients with sepsis**

Vincenza Di Gregorio, Barbara Lattanzi, Daria D'Ambrosio, Rosanna Lacetera, Simone Incicco, Valerio Flavio Fioriti and Manuela Merli

*GastroenterologyDepartment of Clinical Medicine, Sapienza University, Rome, Italy*

**Background and Aim** Severe infections and muscle depletion (MD) represent two entities associated with a poor outcome in cirrhosis. In the general population, MD has been associated to an increased susceptibility to infections due to the immune‐modulatory functions attributed to leptin; conversely, the acute inflammatory state related to sepsis may induce a catabolic state further enhancing malnutrition. Data about the possible synergic effect of sepsis and MD in the outcome of cirrhotic patients are lacking. We aimed at studying the effect of MD on in‐hospital mortality in cirrhotic patients with sepsis.

**Patients and Methods** Consecutive cirrhotic patients with sepsis hospitalized from 2011 to 2015 were enrolled. Patients were classified according to Child‐Pugh class. A diagnosis of MD was made when patients had a Mid Arm Muscle Circumference < 5th percentile.

**Results** Seventy‐four patients with sepsis (71% men; median age 64 yrs) were enrolled. MD was diagnosed in 43% of patients. Severity of liver disease and characteristics of infections were not different in patients with and without MD. A multivariate analysis selected MD (p = 0.0015, OR3.2, IC:1.4–4.8) and Child‐Pugh C (p = 0.001, OR3.3, IC:1.5–4.9) as independent predictors of in‐hospital mortality. A stratified analysis according to the Child‐Pugh class showed that in patients with Child‐Pugh C (29 patients) no differences were observed in in‐hospital mortality according to the presence of MD; on the other hand, in Child‐Pugh A–B (45 patients) MD was associated with a higher rate of mortality (50%vs16%; p = 0.01).

The causes of death were multi‐organ failure (72.3%), hematemesis (15.2%), hepatorenal syndrome (8.7%), and pulmonary edema (3.8%).

**Conclusions** Our study confirms a strong influence of MD on survival in cirrhotic patients with sepsis which is particularly evident in patients with mild‐moderate cirrhosis.

**1-24**

**Sarcopenia predicts the occurrence of hepatic encephalopathy after transjugular intrahepatic portosysthemic shunt**

Silvia Nardelli, Barbara Lattanzi, Stefania Gioia, Vincenza Di Gregorio, Oliviero Riggio and Manuela Merli

*GastroenterologyDepartment of Clinical Medicine, Sapienza University, Rome, Italy*

**Introduction**/**aim**: Hepatic Encephalopathy (HE) is a major problem in patients treated with Transjugular Intrahepatic portosystemic shunt TIPS. Our study was aimed at investigating whether a decrease in muscle mass may independently influence the occurrence of HE after TIPS.

**Patients**/**methods**: Forty‐six consecutive cirrhotic patients submitted to TIPS were included. All patients had a computed tomography scan before TIPS, and the skeletal muscle index (SMI) was evaluated at the level of the third lumbar vertebrae. The presence of “sarcopenia” was defined by sex‐specific cut‐offs. The incidence of the first episode of HE after TIPS, taking into account the competing risk nature of the data (death or liver transplantation), was estimated.

**Results**: Twenty‐six patients (57%) had a diagnosis of sarcopenia. Twenty‐one (46%) patients developed overt HE at a distance of 7 ± 9 months from TIPS procedure. All of them were sarcopenic according to SMI. The difference in the incidence of post TIPS HE between the patients with or without sarcopenia was highly significant (p < 0.0001). At multivariate analysis, MELD score: (sHR:1.16, CI:1.01–1.34, p = 0.043) and sarcopenia: (sHR:31.3, CI:4.5–218.07, p&lt;0.001) were independently associated to post TIPS HE. Both basal ammonia (43.5 ± 18.5 vs 56.8 ± 18.6 µg/dl) and its increment after TIPS (+28.4 ± 11.5 vs +53 ± 12.4 µg/dl) were significantly higher in sarcopenic patients.

**Conclusions**: Muscle wasting, probably by reducing the handling of ammonia, is a risk factor for the development of HE after TIPS. Sarcopenia should be considered in the selection of the patients submitted to TIPS. In sarcopenic patients the amelioration of nutritional status before TIPS can be a possible approach to decrease the incidence of post‐TIPS HE.

**1-25**

**Is muscle wasting a negative prognostic factor in compensated cirrhotic patients?**

Barbara Lattanzi, Vincenza Di Gregorio, Daria D'Ambrosio, Rosanna Lacetera, Rossella Iula and Manuela Merli

*GastroenterologyDepartment of Clinical Medicine, Sapienza University, Rome, Italy*

**Background and Aims**: While the deleterious effect of protein malnutrition in cirrhotic patients with advanced liver disease has been extensively reported, data about the influence of muscle depletion in the setting of compensated liver cirrhosis are scarce. This topic was recently included in the research agenda during the last International Consensus on Portal Hypertension which was held in Baveno in 2015. The aim of the present study was to analyze in hospital survival and rate of complications in relation to muscle depletion in compensated cirrhotic patients (Child Pugh A).

**Methods**: We consecutively enrolled all cirrhotic patients admitted in our department during the last five years and graded as Child score A. Demographical, pathological, and nutritional data were collected for each patient and all complications occurring during the hospitalization were recorded. Tests for minimal hepatic encephalopathy (MHE) were performed in all patients without overt Hepatic Encephalopathy (HE). Patients with MAMC <5th percentile were considered muscle depleted (MD).

**Results**: One hundred three Child A cirrhotic patients were enrolled in the study, 22% were MD. Hepatic encephalopathy was more frequent in muscle depleted patients (18 vs 1.5%, p = 0.001) as well as MHE (64 vs 25%). Renal failure, hyponatremia, and infections occurred in a similar percentage of patients in the two groups. The length of the hospital stay was longer in MD (10 + 9 vs 7 + 5, p = 0.045). In‐hospital mortality was low, as expected in compensated cirrhotic patients, being 3.2% in MD and 1.6% in non‐MD patients (p = 0.8).

**Conclusions**: The presence of MD in compensated cirrhotic patients is associated with an increased prevalence of neurocognitive impairment (both clinical and subclinical) during hospitalization and may extend the length of the hospitalization.

**1-26**

**Handgrip strength, adiposity, and blood pressure in Brazilian type 2 diabetics**

Bruna M. Giglio, Ana C. Marini, Renata C. Fernandes, Reika D. Motobu, Vanessa A. Araújo, João F. Mota and Gustavo D. Pimentel

*Clinical and Sports Nutrition Research Laboratory (Labince)Nutrition Faculty (FANUT), Federal University of Goias (UFG), Goiânia, GO, Brazil*

**Background and aims**: Subjects with type 2 diabetes mellitus frequently are associated with frailty, sarcopenia, metabolic abnormalities, and high costs healthcare. The aim was investigate the type 2 diabetes mellitus prevalence, anthropometric indicators, capillary glycaemia concentrations, and blood pressure levels in Brazilian subjects.

**Methods**: Four hundred twenty‐two individuals of both sexes and 55 years old that frequents five different parks in Goiania City, Brazil, were interviewed by a questionnaire. The type 2 diabetes mellitus diagnostic was self‐reported by subjects. For anthropometric measurements, the weight, height, body mass index (BMI), arm circumference, mid‐arm muscle circumference, triciptal skinfold, calf circumference, abdominal circumference, and handgrip strength. Casual glycaemia concentrations and blood pressure levels were collected. The t Students test was applied to compare type 2 diabetic with non‐diabetic and p < 0.05 was adopted as significant.

**Results**: From 422 subjects, (n = 34) 8% were classified as type 2 diabetes mellitus. We found that diabetic patients had higher BMI (27.6 vs. 25.9 kg/m^2^; p = 0.013), triciptal skinfold (26.1 vs. 23.2 mm; p = 0.031), abdominal circumference (96.4 vs. 91.7 cm; p = 0.013), and lower handgrip strength (27.2 vs. 32.9 kg; p = 0.001) when compared to non‐diabetic subjects. However, arm, mid‐arm muscle, and calf circumference were not altered. In addition, type 2 diabetic patients displayed higher systolic blood pressure (139.3 vs. 129.0 mmHg; p = 0.001), but not diastolic (91.6 vs. 88.7 mmHg; p = 0.166) and glycaemia concentrations (167.4 vs. 99.9 mg/dL; p < 0.000) compared to non‐diabetics.

**Conclusions**: In summary, we found a clear link of low handgrip strength and high adiposity and blood pressure in type 2 diabetics. Additionally, these data reinforce the importance of nutritional interventions to attenuate sarcopenia and prevent high glucose level‐induced metabolic consequences.

**1-27**

**Dysmobility syndrome and its association with mild cognitive impairment in Chilean older people**

Cecilia Albala ^1^, Lydia Lera^1^, Hugo Sánchez^1^, Barbara Angel^1^, Carlos Marquez^1^, Patricia Arroyo^2^ and Patricio Fuentes^2^

^1^ *Institute of Nutrition and Food Technology, Universidad de Chile;* ^2^ *Clinic Hospital University of Chile*

**Background**: Reduced mobility and age‐associated brain physiologic decline are key elements of increased age‐associated vulnerability.

**Objective**. To study the frequency of dysmobility and its association with cognitive impairment in older Chileans.

**Methods**. Follow up of ALEXANDROS cohorts designed to study disability associated with obesity in community dwelling people 60y and older living in Santiago/Chile. At baseline 1006 from 2372 participants had DEXA scan measurements. Dysmobility syndrome was defined has having at least three of the following: slow walk speed (<0.8 m/sec), weak hand‐grip strength ≤ p25 Chilean older people (women ≤15Kg; men ≤27 kg), balance problems, low bone mineral density (<−1SD WHO standard for young people), muscle mass ≤ p20 of appendicular skeletal muscle mass index for Chilean older people (men: 7.19 kg/m2; women: 5.77 kg/m2) and body fat ≥ p60 Chilean older people (men 32%, women 44%). Mild Cognitive impairment (MCI) was defined as having a MMSE test < 22.

**Results**. Prevalence of Dysmobility at baseline was 32.6% (men 23.2%; women 37.0%, p < 0.001) The frequency of dysmobility increased with increasing age (60–69y = 27.9%; 70–79y = 44.2%; ≥80 = 51.0%, p < 0.0001). Dysmobility was associated with mild cognitive impairment (MCI 66.7%; non‐MCI 33.3, p < 0.001). After sex and age adjustment, the OR for the association of dysmobilty with MCI was OR = 3.75 (95% CI: 1.71–8.19). Among the components of dysmobility syndrome, the strongest association was found for low hand‐grip strength (OR = 5.37; 95% CI: 1.27–12.37) followed by low gait‐speed (OR = 2.55; 95% CI:1.91–11.4)

**Conclusion**: Dysmobility is highly prevalent and strongly associated with cognitive impairment in older Chileans. The strongest association was found for hand‐dynamometry and gait‐speed.

Funding: Fondecyt Grant 1130947

**1-28**

**Systemic myostatin: Higher is better? Controversial role in stroke**

Nadja Scherbakov ^1,2^, Andrea Schuette^3^, Anja Sandek^4^, Nicole Ebner^4^, Miroslava Valentova^4^, Stephan von Haehling^4^, Stefan D. Anker^4^, Karl Georg Haeusler^1^, Joerg C. Schefold^5^, Michael Joebges^6^, Lutz Schomburg^3^ and Wolfram Doehner^1,2,7^

^1^ *Center for Stroke Research Berlin CSB, Charité ‐ Universitätsmedizin Berlin, Berlin, Germany;* ^2^ *German Centre for Cardiovascular Research (DZHK), Partner Site Berlin, Berlin, Germany;* ^3^ *Institute for Experimental Endocrinology, Charité ‐ Universitätsmedizin Berlin, Berlin, Germany;* ^4^ *Innovative Clinical TrialsDepartment of Cardiology and Pneumology, University Medicine Goettingen (UMG), Goettingen, Germany;* ^5^ *Department of Intensive Care Medicine, Inselspital, Bern University Hospital, Switzerland;* ^6^ *Department of Neurology, Brandenburgklinik Bernau, Bernau, Germany;* ^7^ *Department of Cardiology, Charité ‐ Universitätsmedizin Berlin, Berlin, Germany*

**Introduction**: Myostatin (MSTN) is an inhibitor of skeletal muscle cells growth and differentiation. The increased expression of this protein in skeletal muscle contributes to the pathogenesis of sarcopenia. However, data regarding systemic MSTN levels are controversial. The aim of the present study was to investigate systemic levels of MSTN and its relation to the muscle mass and muscle function in the patients with stroke.

**Methods**: A total of 189 stroke patients (112 patients with acute ischemic stroke, 3 ± 2 days after stroke onset, BMI 28.0 ± 4.6 kg/m^2^, 69 ± 14 years, and 77 BMI‐ and age‐matched patients with subacute stroke, 26 ± 25 days after stroke onset) were prospectively studied. Body composition was assessed by bioelectrical impedance analysis and skeletal mass index (SMI) was calculated. Barthel index (BI), modified Rankin scale (mRS), and handgrip test were performed at enrollment. Sarcopenia was defined according to European Working Group on Sarcopenia in Older People (EWGSOP) criteria. Systemic concentrations of MSTN were measured from frozen serum samples. Ten BMI‐ and age‐matched healthy individuals served as a control.

**Results**: Sixteen patients (14%) with acute stroke and 16 patients (21%) with subacute stroke were identified as sarcopenic. Sarcopenic patients demonstrated lower levels of serum MSTN compared to patients without sarcopenia or controls (1914 ± 980 vs. 2405 ± 1050 vs. 3250 ± 1101 pg/ml, p < 0.01) respectively. Serum levels of MSTN correlated with lean mass (r = 0.18, p = 0.01), inversely with fat mass (r = −0.2, p = 0.003), SMI (r = 0.3, p < 0.01), BI (r = 0.33, p < 0.001), mRS (r = 0.23, p < 0.01), and handgrip strength (r = 0.2, p = 0.005). In multiple logistic regression analysis, sarcopenia was related to MSTN levels in female patients but not in males. According to receiver operator characteristics, MSTN levels <1514 pg/ml identified sarcopenic stroke patients with a sensitivity of 80.1% and a specificity of 45.5%. The area under the curve was 0.65 (95% confidence interval 0.55‐0.75).

**Conclusion**: Assessments of systemic MSTN levels may provide additional information to identify sarcopenia in patients with stroke. Large prospective studies investigating the potential role of systemic MSTN levels in sarcopenia are warranted.

**1-29**

**Electrical impedance myography correlates with muscle mass and function in a cohort at risk for sarcopenia**

W. David Arnold ^1^, Seward B. Rutkove^2^, Martin D. Buck^3^, Jose Bohorquez^3^ and Jonathan F. Bean^4,5^

^1^ *Department of NeurologyDepartment of PM&RDepartment of Neuroscience, The Ohio State University Wexner Medical Center, Columbus, Ohio, 43210;* ^2^ *Department of NeurologyBeth Israel Deaconess Medical Center, Harvard Medical School, 330 Brookline Avenue, Boston, MA, 02215;* ^3^ *Skulpt, Inc, 333 Bryant Street, Suite 220, San Francisco, CA, 94107;* ^4^ *New England GRECCVA Boston Healthcare System, Bedford VAMC, Boston, MA, 02130;* ^5^ *Department of PM&R, Harvard Medical School, Boston, MA, 02115, USA*

**Background and aims**: Sarcopenia, aging‐related loss of muscle mass and strength, is a major health concern in the developed world. We aimed to assess reliability of self‐administered electrical impedance myography (EIM) in a population at risk for sarcopenia and to compare the findings in the quadriceps muscle to standard assessments of muscle size and function.

**Methods**: EIM was measured at the biceps, volar forearm, dorsal forearm, quadriceps, gastrocnemius, and tibialis anterior. Two trials were performed by the participants on themselves and compared with one trial by a trained evaluator to calculate intraclass correlation coefficients (ICC); 50 kHz EIM phase and reactance of the quadriceps were compared to the Short Physical Performance Battery (SPPB), measures of muscle strength, power, and velocity obtained during single leg press, and measures of body composition.

**Results**: Twenty‐eight participants (14 men, 14 women) were enrolled with a mean age of 79.6 ± 6.4 years. Mean SPPB was 9.8 ± 2.0. EIM showed good intra (ICC = 0.90) and interrater (0.84) reliability. Reactance showed significant correlations with SPPB (r = 0.48, p = 0.01) and the mobility sub‐components [4 meter walk test (r = 0.51, p < 0.01) and repeat chair stands (r = 0.42, p = 0.03)] as well as strength (r = 0.42, p = 0.03) and power (r = 0.41, p = 0.04) but not velocity. Phase only correlated with strength (r = 0.44, p = 0.02). In contrast, reactance did not correlate with measures of body composition, whereas phase was correlated with total body impedance analysis (r = 0.82, p < 0.001) and lean lower limb mass on DEXA (r = 0.56, p < 0.01).

**Conclusions**: EIM is a non‐invasive and simple to perform measure of neuromuscular function which has shown good sensitivity to disease status in a variety of neuromuscular disorders. This study provides strong evidence that parameters of EIM measures are good biomarkers for body composition and neuromuscular function in aging populations. Importantly, our findings suggest that EIM can be reliably self‐administered in populations at risk for developing sarcopenia and used for assessing preventive and therapeutic interventions.

**1-30**

**Arterial tonometry as a method to assess endothelial dysfunction in chronic heart failure‐relationship to exercise capacity and muscle wasting**

Nicole Ebner ^1^, Nadja Scherbakov^2^, Anja Sandek^1^, Stefan D. Anker^1^, Stephan von Haehling^1^ and Wolfram Doehner^2^

^1^ *University Medical Center GöttingenCardiology and Pneumology, Göttingen, Germany;* ^2^ *Center for Stroke Research CSB, Charite University Medical School, Berlin*

**Background**: Endothelium dysfunction [ED] is a prominent feature in the pathophysiology of cardiovascular disease. The impact of ED on atherogenesis and increased cardiovascular risk is clearly established. Current assessment methods of ED are, however, complicated by invasive protocols or laborious Doppler ultrasound procedures. The aim of the study was to examine endothelial dysfunction in patients with CHF by a novel non‐invasive and easily applicable method in relation to exercise capacity and clinical status in patients with chronic heart failure.

**Methods**: We studied 75 patients with chronic HF [age 65 ± 11 years, 24% female, body mass index [BMI] 28.6 ± 5.5 Kg/m^2^, New York Heart Association [NYHA] class (I/II/III/IV, 4/41/24/3), left ventricular ejection fraction [LVEF] 36 ± 11%, pVO_2_ 16.6 ± 5.0 ml/min/Kg (all mean ± SD)]. Endothelial dysfunction was assessed by non‐invasive arterial tonometry [EndoPAT] using the reactive hyperaemia index [RHI]. RHI is defined as a ratio between the post‐ and pre‐occlusion arterial tonometry signal of the index finger corrected for baseline vascular tone and for the signal of the non‐occluded contra lateral arm. Exercise capacity was assessed by symptome limited treadmill spiroergometric exercise test (modified Bruce protocol) and six‐minute walk test (6MWT). For comparison, we studied 20 healthy controls [CON] of similar age and gender distribution. Appendicular skeletal muscle mass was assessed by dual energy X‐ray absorptiometry (DEXA) and compared with a healthy reference group of young adults aged 18–40 years. Sarcopenia was defined as appendicular muscle mass 2 SD below the mean of the young reference group, and Präsarcopenia as 1 SD below the mean of the reference group, as suggested for the diagnosis of sarcopenia.

**Results**: RHI was significantly decreased in chronic HF compared to controls (1.80 ± 0.36 vs 2.14 ± 0.62, p = 0.007) and was lowest in patients with advanced chronic HF (NYHA III/IV: 1.7 ± 0.5, p < 0.01). RHI was more reduced in chronic HF patients with ischaemic aetiology than in non‐ischaemic CHF (non‐ischaemic HF vs ischaemic HF, 1.96 ± 0.5 vs. 1.64 ± 0.3; p < 0.05). In linear regression analyses lower RHI was associated with lower pVO_2_ (r = +0.30, p < 0.05) as well as lower 6‐minute walk test (r = +0.40, p = 0.01) and with age (r = +0.35, p < 0.01). 6MWT distance was reduced in chronic HF vs CON (413.5 ± 145.5 m vs 556.4 ± 100.2 m, p < 0.001). RHI was further predicted by age (r = −0.35, p < 0.01). Sarcopenia was detected in 15 patients with chronic HF and Präsarcopenia is 25 patients with chronic HF. RHI was significantly decreased in patients with sarcopenia compared to patients without sarcopenia (p < 0.05).

**Conclusions**: Endothelial dysfunction as assessed by EndoPAT is predictive of reduced functional status and impaired exercise capacity in patients with chronic HF. Endothelial dysfunction may impact on development of poor muscle perfusion, particularly during exercise, and contribute to skeletal muscle wasting. Assessment of endothelial function by this novel non‐invasive method using reactive hyperaemia is a simple and easily applicable method for the use in ambulatory and clinical settings and can be used in patients with and without cachexia.

**1-31**

**Human skeletal muscle changes in improved metabolic health**

Yotam Raz ^1^, Erik van den Akker^1,2^, Joris Deelen^1^, Eka Suchiman^1^, Tijmen Roest^1^, Hailiang Mei^1^, Muhammad Riaz^3^, Vered Raz^3^, Marian Beekman^1^ and Eline Slagboom^1^

^1^ *Molecular Epidemiology, Leiden University Medical Center, Leiden, The Netherlands;* ^2^ *Delft Bioinformatics Lab, Delft University of Technology, Delft, The Netherlands;* ^3^ *Human Genetics, Leiden University Medical Center, Leiden, The Netherlands*

One of the challenges in aging populations is to unravel aging‐associated, metabolic, and life‐style determined diseases. Decrease in musculoskeletal functionality during muscle aging hampers mobility and daily functioning and is known to be affected by poor metabolic health. Moreover, aging‐associated metabolic changes in the muscle tissue can lead to changes in whole‐body metabolism. Such metabolic changes include shifts in the oxidative and glycolytic metabolic capacities and accumulation of lipid droplets with an adverse extra‐cellular environment and are key features of muscle aging. While in early adulthood unfavorable metabolic health leads to detrimental metabolic and architectural changes in the muscle tissue, the process of muscle aging is still poorly understood for older adults. We aim to elucidate how improving whole‐body metabolic health impacts on elderly human skeletal muscles at molecular and tissue levels. For this study we investigated muscle biopsies from 87 older adults (mean age 63 years) before and after a 13‐week lifestyle intervention (the Growing Old Together Study). From both muscle and blood, before and after the intervention, RNA sequencing data were generated, and the molecular signature of the lifestyle intervention was explored in the transcriptomes. Additionally, aging‐associated metabolic and architectural changes in the muscle tissue were studied by novel procedures of semi‐automated quantitative immunofluorescence imaging. Furthermore, whole body metabolism was assessed by traditional clinical markers as well as with ^1^H‐NMR serum metabolites. We aspire to identify novel biomarkers of healthy muscle aging and further the knowledge of tissue‐specific responses to lifestyle interventions.

**1-32**

**The creation of uniform cut‐off values for skeletal muscle mass measurements on abdominal computed tomography scans in healthy subjects**

Jeroen L.A. van Vugt, Yordi van Putten, Ron W. F. De Bruin, Hendrikus J.A.N. Kimenai and Jan N.M. IJzermans

*Department of Surgery, Erasmus University Medical Center, Rotterdam, The Netherlands*

**Introduction**: Currently, there is much debate regarding adequate cut‐off values for computed tomography (CT)‐assessed skeletal muscle mass to categorize patients as (non)sarcopenic. No international consensus has been reached yet, and there is insufficient knowledge on skeletal muscle mass in healthy persons stratified for sex, age, and BMI.

**Methods**: Routinely performed contrast‐enhanced abdominal CTs of living kidney donors between 2010 and 2015 were collected. The cross‐sectional skeletal muscle area was measured and corrected for patients' height (Skeletal Muscle Index [SMI]; cm^2^/m^2^). Mean Skeletal Muscle Density (SMD; in Hounsfield Units [HU]) was recorded as a measure of skeletal muscle quality. Patients were categorized by age (20‐29, 40‐59, and ≥60) and BMI (<20, 20.0–24.9, 25.0–29.9, ≥30 kg/m^2^). Groups were compared using Mann–Whitney‐U and Kruskal–Wallis tests.

**Results**: In total, 627 patients with available CT scans were identified. The current cohort consisted of 241 patients, of whom 104 (43.2%) were male. Median age and BMI were 51 (interquartile range [IQR] 39–60) and 25.4 (IQR 23.5–28.7) kg/m^2^, respectively. ASA classification was 1–2 in 237 (99.2%) patients. Median SMI (57.0 versus 43.4 cm^2^/m^2^, p < 0.001) and SMD (45 versus 43 HU, p = 0.005) were significantly higher in males compared with females. Significant decreases per age group were observed for SMI (youngest 51.1 versus oldest 45.9 cm^2^/m^2^, overall p = 0.015) and SMD (youngest 49 versus oldest 38 HU, overall p < 0.001) in males. In females, significant decreases in SMD (p < 0.001), but non‐significant decreases in SMI (p = 0.100) were found. SMI significantly increased per BMI group for both sexes (p < 0.001), while SMD significantly decreased (males p = 0.034, females p < 0.001).

**Conclusion**: This is the first study describing sex, age, and BMI‐specific CT‐assessed SMI and SMD measures in healthy Western subjects. Currently, the cohort is being enlarged and cut‐off values are being defined, but these first results underline the need for stratified cut‐off values to compare various patient populations.

**1-33**

**The effect of contrast‐enhancement on computed tomography‐based skeletal muscle mass and skeletal muscle density measurements**

Jeroen L.A. van Vugt ^1^, Kevin M. Veen^1+^, Henk‐Jan W. Schippers^1+^, Robert R.J. Coebergh van den Braak^1^, Stef Levolger^1^, Ron W. F. de Bruin1, Jan N.M. IJzermans^1^ and François E.J.A. Willemsen^2^

^1^ *Department of Surgery, Erasmus University Medical Center, Rotterdam, The Netherlands;* ^2^ *Department of Radiology, Erasmus University Medical Center, Rotterdam, The Netherlands*

*†Contributed equally to this study*

**Introduction**: Skeletal muscle mass (SMM) and density (SMD) are often measured on computed tomography (CT) scans without taking different contrast‐enhancement phases into account. Possible consequences as a result of from contrast‐enhancement remain unknown.

**Methods**: Fifty multiphase (unenhanced, arterial, (portal)venous) abdominal CT‐examinations were randomly selected. Cross‐sectional skeletal muscle area corrected for patients height (skeletal muscle index [SMI]; in cm^2^/m^2^) and density (SMD; in Hounsfield units [HU]) were measured by two observers, at the level of the third lumbar vertebra on preselected slices. The average of the two measurements was used for analysis. Low SMM was defined as SMI < 41 cm^2^/m^2^ (women), and <43 (men, BMI <25 kg/m^2^) or <53 (men, BMI ≥25 kg/m^2^). Agreement between enhancement phases for SMM and SMD was calculated using intra‐class correlation coefficients (ICCs). Cohen's κ's were calculated for the agreement of sarcopenia assessment.

**Results**: The study cohort included 27 (54%) males. The Mean BMI was 24.2 (standard deviation [SD] 4.0) kg/m^2^. Mean SMI was 42.5 (SD 9.9) cm^2^/m^2^ on enhanced phase, compared with 42.8 (SD 9.9) and 43.6 (SD 9.9) for the arterial and portal‐venous phase, respectively (p < 0.01). Mean SMD was lower for the unenhanced phase (30.9; SD 8.0) compared with the arterial (38.0; SD 9.9) and portal‐venous (38.7; SD 9.2) phase (both p < 0.001). No significant difference was found between the mean SMD in the portal‐venous and arterial phase (p = 0.161). The ICCs were excellent (≥0.992) for all SMIs and for SMD between the contrast‐enhanced phases (0.949). The ICCs for the blanc phase compared with the arterial (0.676) and portal‐venous (0.665) phase were considered fair to good. The Cohen's κ's for sarcopenia assessment was excellent (0.88–0.96).

**Conclusion**: Significant, but clinically unimportant differences were found in SMI between (non)contrast‐enhanced phases. Contrast‐enhancement strongly influenced SMD measurements. Therefore, we recommend that studies using this measurement should include (non)contrast‐enhanced CTs only.

**1-34**

**Limb immobilisation for 3 d markedly reduces deuterium oxide (D2O)‐derived muscle protein synthesis despite limited changes in atrophy‐related pathways in young healthy humans**

Arfan M. Ali ^1^, Brigitte E. Scammell^1^, Tariq Taylor^2^, Joanne E. Mallinson^2^, Robert W. Kerslake^1^, Matthew Brook^2^, Daniel J. Wilkinson^2^, Kenneth Smith^2^, Marco V. Narici^2^, Phil J. Atherton^2^ and Paul L. Greenhaff^1,2^

^1^ *ARUK Centre for Sport, Exercise and Osteoarthritis;* ^2^ *MRC/ARUK Centre for Musculoskeletal Ageing ResearchFaculty of Medicine and Health Sciences, University of Nottingham*

**Background**: Quantification of muscle protein synthesis (MPS) using stable‐isotope tracer infusions pre‐ and post‐disuse is well documented, but no study has documented changes in MPS over the course of a sustained period of immobilisation. Furthermore, little information is available regarding the molecular basis of disuse atrophy under these conditions. We therefore assessed the impact of 3 d of unilateral, below knee limb immobilisation upon chronic myofibrillar protein fractional synthetic rates (FSR) and expression levels of putative regulators of anabolic, catabolic, and endoplasmic reticulum stress (ER)‐related pathways.

**Methods**: Twelve young volunteers (6 M/6 F, 25.1 ± 5.2 years) ingested a single 150 ml bolus of deuterium oxide (D_2_O, 70%) 3–4 d before unilateral, limb immobilisation. Muscle biopsies were obtained in the fasted state from the medial gastrocnemius muscle of the non‐immobilised limb before D_2_O ingestion and before limb immobilisation, and from both limbs immediately after 3 d immobilisation. Muscle tissue was used to determine myofibrillar protein FSR in both limbs (n = 7, 4 M/3 F) and protein expression and phosphorylation levels using western blotting (n = 12). Data are mean ± SEM. Differences between limbs detected using Student's paired *t*‐test.

**Results**: Chronic muscle protein FSR was ~24% less in the immobilised limb when comparing limbs (1.4 ± 0.1%^.^d^‐1^ vs 1.0 ± 0.1%^.^d^‐1^, *p* < 0.01). No major differences were observed when comparing target protein expression levels between limbs (*phospho*‐AKT^Ser473^, *total*‐AKT, *total*‐mTOR, *phospho*‐P70S6K^Thr389^, *total*‐4EBP1, *total*‐ATF4, *total*‐CHOP, *total*‐FoxO1, *total*‐FoxO3, and *total*‐Caspase‐3), with the exception of *phospho*‐4EBP1^Thr37/46^ which was 28 ± 16% less in the immobilised limb *p* < 0.05.

**Conclusion**: Chronic myofibrillar protein FSR is ~24% less over 3 d of unilateral lower limb immobilisation compared to the non‐immobilised contralateral limb in healthy, young volunteers. This occurred in the absence of robust differences in expression of putative regulators of muscle protein synthesis, catabolism, and ER stress with exception of *phospho*‐4EBP1. Inhibition of cap‐dependent initiation may be implicated in the chronic suppression of myofibrillar FSR in response to immobilisation.

**1-35**

**Untargeted metabolomics to identify links between ageing and musculoskeletal health in humans**

Daniel J. Wilkinson ^1,2^, Warwick B. Dunn^1,3,4^, Bethan E. Phillips^1,2^, Giovanny Rodriguez‐Blanco^3,4^, Andrew Chetwynd^3,4^, Kenneth Smith^1,2^ and Philip J. Atherton^1,2^

^1^ *MRC‐Arthritis Research UK Centre for Musculoskeletal Ageing Research;* ^2^ *School of Medicine, University of Nottingham, Royal Derby Hospital Centre, Derby, DE22 3DT, UK;* ^3^ *School of Biosciences;* ^4^ *Phenome Centre Birmingham, University of Birmingham, Birmingham, B15 2TT, UK*

**Background and aims**: Resistance‐exercise‐training (RET) increases muscle mass and function and currently provides the most effective countermeasure against age related musculoskeletal decline (sarcopenia). Yet the responses to RET in older adults have been shown to be impaired when compared to young. The aim of this study was to use an untargeted metabolomics approach to assess the impact of ageing on the muscle metabolome, whether the metabolome could be altered by a program of resistance exercise training and how this differed across age groups.

**Methods**: Muscle samples from three age groups (18–30 y N = 11, 45–55 y N = 20 and 65–75 y N = 20) were collected in the post‐absorptive state, and following acute resistance exercise both prior to and following 20 weeks of supervised whole body resistance exercise training. Muscle samples were extracted and analysed using HILIC and reversed phase UHPLC‐MS. All data were analysed applying univariate and multivariate statistics to assess associations between metabolite concentrations, age, and exercise response.

**Results and Conclusions**: There was clear divergence between the age groups for the muscle metabolome. Young showed significantly higher levels of TCA cycle intermediates, branched chain amino acids and energy metabolites (phosphocreatine (PCr) and NAD) immediately following a single bout of resistance exercise compared to middle and older aged. Moreover, young and middle aged showed higher levels of the NAD and PCr following 20‐week RET, suggesting an age‐related impairment in the regulation of skeletal muscle energetic pathways, which may contribute to the overall age related musculoskeletal decline. These data in turn highlight the power of metabolomics for uncovering discrete differences between age groups and the potential of this approach for creating prognostic and diagnostic biomarkers of age related muscle impairment.

**1-36**

**Heart failure symptoms, handgrip strength and nutritional status have an impact on frailty in outpatients with chronic heart failure in Japan**

Norio Suzuki ^1^, Keisuke Kida^2^, Chikayuki Ito^2^, Kohei Ashikaga^2^, Kengo Suzuki^2^, Kazuto Omiya^1^, Tomoo Harada^2^ and Yoshihiro J. Akashi^2^

^1^ *Division of CardiologyDepartment of Internal Medicine, St. Marianna University School of Medicine Yokohama City Seibu Hospital, Yokohama, Japan;* ^2^ *Division of CardiologyDepartment of Internal Medicine, St. Marianna University School of Medicine, Kawasaki, Japan*

**Background and aims**: This study investigated the usefulness of novel frailty index calculated according to the 5‐item self‐report questionnaire for frailty, which included nutrition/shrinking, physical function, physical activity, forgetfulness, and emotions/exhaustion, and the frailty‐associated factors in patients with chronic heart failure (CHF).

**Methods**: Totally, 135 outpatients with CHF aged over 65 years were enrolled. All study patients filled in the frailty index. The patients were stratified into the frailty (≥3 frailty scores), prefrailty (1 or 2 frailty scores), or robust group. Nutritional status was assessed by the Mini Nutritional Assessment Short Form (MNA®‐SF). Moreover, low handgrip strength was defined as <26 kg for male and <18 kg for female by Asian Working Group for Sarcopenia. Heart failure hospitalization and all‐cause mortality were defined as an event.

**Results**: The mean age was 76.0 ± 7.4 years, and left ventricular ejection fraction was 43.6 ± 17.2%. Of the study patients, 54.7% patients were male, 20.7% patients had ischemic heart failure, 45.9% patients had MNA®‐SF score ≤11, 20.0% patients had frailty, and 45.2% patients had prefrailty. The 180‐day event‐free survival rates were 66.9%, 94.9%, and 95.7% in the frailty, prefrailty, and robust groups (Log‐rank, p = 0.007). The multivariate logistic regression analysis indicated that the hazard ratios were 6.70 in the New York Heart Association (NYHA) classification 2 or more (95% confidence interval, CI; 1.46–49.15, p = 0.013), 3.42 in low handgrip strength (95% CI, 1.03–14.72, p = 0.047), and 4.25 in the MNA®‐SF score ≤11 (95% CI, 1.29–15.26, p = 0.017), suggesting that these ratios might be independent predictors for frailty.

**Conclusions**: The CHF outpatient with frailty based on novel frailty index was a significantly poor prognosis. Then, severity of heart failure symptoms, low handgrip strength, and malnutrition were possible predictive factors for frailty. They are often reversible; thus, early detection and intervention are necessary for its improvement.

**1-37**

**Cutt‐off points for skeletal muscle mass index estimated using a bioelectrical impedance analysis**

Mirele Savegnago Mialich, Bruna Ramos da Silva and Alceu Afonso Jordão

*Department of Internal MedicineRibeirao Preto Medical School, University of Sao Paulo, Ribeirao Preto, SP, Brazil*

**Background and Objectives**: The clinical definition of sarcopenia involved gait speed, grip strength, and muscle mass measurements, and the low muscle mass is prevalent in older populations. In this study, we aimed to define the cut‐off thresholds for skeletal muscle mass (SMM) from Brazilian in order to improve the diagnostic criteria for sarcopenia for this population.

**Subjects and Methods**: Healthy young adults (18–39 years) served as reference population for assessing SMM threshold data. Another group (>60 years) was recruited to serve as the older reference population. Body composition was assessed with bioimpedance analysis using a Tanita BC 558 monitor. For statistical analyzes, the cut‐offs threshold for skeletal muscle mass index (SMMI) were defined as the mean −2SD of the values of the young reference study population.

**Results**: The young reference group included a total of 468 participants (127 men, 341 women). Mean age was 20.6 ± 2.4 years, SMMIs 10.8 ± 1.1 kg/m^2^, and 8.5 ± 0.7 kg/m^2^. The SMMI cut‐off points were designated as 8.6 kg/m^2^ and 7.1 kg/m^2^, in men and women, respectively. And these data are consistent with values of young populations of reference from different nations, e.g. 9.2 kg/m^2^ and 7.4 kg/m^2^ in Turkey (Bahat et al., 2016); 8.3 kg/m^2^ and 6.7 kg/m^2^ in Spain (Masanes et al., 2012); 8.9 kg/m^2^ and 6.5 kg/m^2^ in Taiwan (Chien et al., 2008); 8.6 kg/m^2^ and 6.2 kg/m^2^ in France (Tichet et al., 2008), for men and women, respectively. The prevalence of low muscle mass in older reference population (n total = 180, 58 men, 122 women) was 4,4% [n = 6 (10.3%) in men; n = 2 (1,7%) in women].

**Conclusions**: This study corroborates other studies in the literature, which converge on similar cut‐off point for SMMI. Future studies aim to supplement this data with additional measures of gait speed, grip strength to integrate the diagnostic criteria for definition of sarcopenia.

**1-38**

**Impact of muscle mass, evaluated by ultrasound, and muscle strength in the engraftment of Hematopoietic Stem Cell Transplant (HSCT) patients**

Andrea Z. Pereira ^1^, Juliana B.S. Barban^2^, Marister N. Cocco^1^, Bianca A. Pitito^3^ and Nelson Hamershlak^1^

^1^ *Oncology and Hematology Department, Hospital Israelita Albert Einstein, S. Paulo, SP, Brazil;* ^2^ *Hematology Department, UNIFESP, S. Paulo, SP, Brazil;* ^3^ *Public Health Department, UNIFESP, S. Paulo, SP, Brazil*

**Introduction**: In the HSCT, muscle mass and visceral fat are associated with comorbidities, mortality, length of stay, duration of use of immunosuppressive drugs, the development of graft‐versus‐host disease (GVHD), and survival. A recent study in patients undergoing allogeneic HSCT showed an inverse association between areas of visceral and peripheral fat with disease‐free interval. In allogeneic HSCT, decreased muscle mass is associated with a higher prevalence of chronic GVHD and low performance.

**Objectives**: To evaluate the thickness of the quadriceps femoris muscle and visceral fat (VF) and the muscle strength (MS), correlating them with time to engraftment (EN).

**Methods**: We studied 14 HSCT patients (≥18 years), Hospital Israelita Albert Einstein, São Paulo, Brazil, in the first day of hospitalization, before HSCT. We measured the thickness of the right femoral quadriceps muscle (RFQ), 6 cm from the top edge of the patella, using ultrasound (US) in B‐mode. The VF was measured in the abdominal region and was characterized by the distance between the linea alba and the anterior wall of the aorta artery. In addition, all patients had their dominant upper limb strength evaluated by hand grip.

**Results**: Most patients were women (57%) with a mean age of 50 years (±16 years) and 50% of our patients were elderly (≥60 years). The haploidentical (57%) was the predominant HSCT, autologous (36%) and allogeneic (7%). Most of our patients were overweight, with body mass index (BMI) of 27 kg/m2 (±4 kg/m2). The average time EN was 16 days (±6 days). RFQ was 1.5 cm (±0.3 cm), the VF was 5,3 cm (±1.4 cm), and MS was 31 kgf (±7,0 kgf). There was a negative correlation between EN and RFQ (rs = 0.8, p <0.05), independent of the age and the HSCT type by Linear Regression. There was no significant correlation between VF and MS with EN.

**Conclusion**: The RFQ showed a strong correlation with EN. The ultrasound is practical, low cost, no risk in HSCT; it could be more used for these patients.

**2-01**

**Alterations of mitochondrial quality control checkpoints in skeletal muscle of cachectic patients with gastric cancer**

Anna Picca ^1^, Maria Lorenzi^1^, Riccardo Calvani^1^, Francesco Landi^1^, Fausto Rosa^2^, Giovanni Battista Doglietto^2^, Amerigo Menghi^1^, Roberto Bernabei^1^, Maurizio Bossola^1,2^ and Emanuele Marzetti^1^

^1^ *Department of GeriatricsNeurosciences and OrthopedicsCatholic University of the Sacred Heart School of Medicine, Teaching Hospital “Agostino Gemelli”, Rome, Italy;* ^2^ *Department of SurgeryCatholic University of the Sacred Heart School of Medicine, Teaching Hospital “Agostino Gemelli”, Rome, Italy*

**Background and aims**: Cancer cachexia (CC) is a multifaceted debilitating syndrome featured by body weight loss mainly due to skeletal muscle wasting. The mitochondrial involvement in muscle wasting has attained consensus over time, although its role in the pathogenesis of CC is still unclear. We investigated mitochondrial quality control (MQC) signalling in muscle and cachexia in patients with gastric cancer.

**Methods**: Biopsies from the rectus abdominis muscle of 18 patients with gastric cancer (9 with CC and 9 non‐cachectic (NCC)) were collected and assayed for the expression of a set of MQC mediators.

**Results**: Mitochondrial plasticity was analyzed first, and no changes were found between groups in the protein content of either mitofusin 2 (Mfn2) or optic atrophy protein 1 (OPA1). CC patients, instead, showed an up‐regulation of fission protein 1 (Fis1) gene expression relative to NCC. The calculation of the “fusion index” (Mfn/Fis1 protein ratio), as a measure of cell ability to compensate for mitochondrial impairment, revealed a failure for such a compensation in CC patients. As for mitophagy, there was no difference in the expression of the PTEN‐induced putative kinase 1 (PINK1) between groups, but interestingly, the protein ratio of the lipidated and non‐lipidated form of microtubule‐associated protein 1 light chain 3B (LC3B II/LC3B I), an index of ongoing autophagy, showed a decrease in CC patients compared with NCC counterpart. Neither the protein expression of autophagy‐associated protein 7 (Atg7) and lysosome‐associated membrane protein 2 (LAMP‐2) nor the mRNA abundance of the mitochondrial biogenesis factors peroxisome proliferator‐activated receptor‐γ coactivator‐1α (PGC‐1α), and mitochondrial transcription factor A (Tfam) were changed between groups.

**Conclusions**: Our results suggest an association between CC and derangements in mitochondrial dynamics, tagging for disposal, and execution of mitophagy, these latter representing checkpoints of the MQC and precious elements for the identification of targets for pharmacological interventions.

**2-02**

**Peptidic and Nonpeptidic Growth Hormone Secretagogues exert a protective effect on mitochondrial parameters analysed in a rat model of cachexia**

Giuseppe Sirago ^1^, Flavio Fracasso^1^, Antonella Liantonio^2^, Elena Conte^2^, Giulia Maria Camerino^2^, Antonio Torsello^3^, Jean‐Alain Fehrentz^4^, Jean Martinez^4^, Vito Pesce^1^ and Palmiro Cantatore^1^

^1^ *University of Bari (Italy)Department of Biosciences, Biotechnologies and Biopharmaceutics;* ^2^ *University of Bari (Italy), Department of Pharmacy‐Drug Sciences;* ^3^ *University of Milano‐Bicocca (Monza, Italy), Department of Health Sciences;* ^4^ *University of Montpellier (ENSCM, Montpellier, France), Max Mousseron Institute of Biomolecules UMR5247, CNRS*

**Background and aims**: Cachexia and muscle atrophy are common derivations of cancer and chemotherapy treatments (cisplatin). Growth Hormone Secretagogues (GHSs) are synthetic peptidic and nonpeptidic molecules, able to stimulate Growth Hormone secretion and to target a specific receptor in skeletal muscle counteracting cachexia.

We report the effects of GHSs on skeletal muscle mitochondrial biogenesis and dynamics in a rat model of cisplatin‐induced cachexia.

**Methods**: Cachexia was induced in adult rats by intraperitoneal injection of cisplatin (1 mg/kg) once daily for 3 days. The treatment with GHSs was hexarelin, 160 µg/Kg and JMV2894, 320 µg/Kg, ip, and b.i.d, for 5 days.

**Results**: We measured in rat tibialis anterior of cisplatin treated group, a decrease of the level of mtDNA, PGC‐1α and TFAM (proteins involved in mitochondrial biogenesis), PRX3 and SOD2 (antioxidant mitochondrial enzymes) and an increase of the oxidized total cellular PRXs which suggest an increase of ROS damages. GHSs treatment increased mtDNA, PGC‐1α and TFAM, PRX3 and SOD2 level while reduced the oxidized total cellular PRXs. The MFN2 and Drp1 proteins level increased with cisplatin administration, whereas it decreased after GHSs treatment. These results demonstrated that cisplatin treatment depresses several parameters linked to mitochondrial biogenesis and integrity and that the GHSs administration prevents these alterations. It is involved in the disease the activation of proteolysis due to AKT and FoxO3a dephosphorylation, whereas PGC‐1α is able to inhibit the transcriptional activity of FoxO3a, suppressing atrogenes expression and protein degradation.

In particular, GHSs stimulated the phosphorylation of AKT and FoxO3a, thus inducing a recovery of skeletal muscle mass. In addition, the here reported increase of PGC‐1α prevents the activation of atrogenes by blocking the FoxO3a function.

**Conclusions**: These data indicate that treatment with GHSs exert a muscle protective effect in cisplatin‐induced model of cachexia and may be a therapeutic promising tool for supportive care in cachexia.

**2-03**

**Skeletal muscle myotubes show decreased viability and protein synthesis after treatment with chemotherapy**

Francina J. Dijk ^1^, Miriam van Dijk^1^, Bram Dorresteijn^1^, Marion Jourdan^1^, Onno Kranenburg^2^ and Yvette Luiking^1,3^

^1^ *Nutricia Advanced Medicial Nutrition, Nutricia Research, Utrecht, The Netherlands;* ^2^ *Department of Surgical OncologyCancer Centre, UMC Utrecht, Utrecht, The Netherlands;* ^3^ *Center for Translational Research in Aging and LongevityDepartment of Health and Kinesiology, Texas A&M University, USA*

**Background and aims**: Chemotherapy is a very non‐specific treatment that targets all high proliferating cells like cancer cells, but also healthy cells like skeletal muscle cells. Cancer itself can cause muscle wasting (cachexia); however, limited information is available on the direct effect of chemotherapy on skeletal muscle. Therefore, we aimed to study the effect of chemotherapy on protein synthesis (MPS), viability, necrosis, and apoptosis in a model for skeletal muscle (C2C12 myotubes).

**Methods**: Differentiated myotubes were incubated for 24 h with a concentration range of irinotecan (IR; 125‐2000 μM), oxaliplatin + 5‐Fluorouracil (OXF; 2–250 μM), or left untreated (control). Chemotherapy treatment was followed by a 4‐h serum‐ and leucine‐free period. Subsequently, viability was measured by CellTiter‐Glo, necrosis by LDH‐release, and apoptosis by caspase‐3/7 activity. MPS was measured with the SUnSET method after 1‐h stimulation with anabolic stimuli insulin (100nM) and leucine (5 mM), or in myotubes left unstimulated (basal state).

**Results**: IR and OXF treatment decreased cell viability and increased necrosis and apoptosis dose‐dependently. MPS decreased dose‐dependently in both stimulated (IR‐IC50 = 1608 μM, OXF‐IC50 = 23.7 μM) and unstimulated treated myotubes (IR‐IC50 = 1645 μM, OXF‐IC50 = 20.3 μM). IR decreased MPS in unstimulated myotubes only at concentrations 1500–2000 μM (P < 0.05 vs control). MPS response to insulin‐leucine stimulation was observed at IR concentrations 125–250 μM (P < 0.05 vs unstimulated); however, it remained below the level of stimulated controls (P < 0.05). OXF decreased MPS in unstimulated myotubes at all concentrations (P < 0.05 vs control). Insulin‐leucine stimulation increased MPS at 2–32 μM OXF (P < 0.05 vs unstimulated), but could not restore MPS to levels of stimulated controls.

**Conclusion**: Treatment of myotubes with chemotherapeutics IR or OXF causes cell death by apoptosis and necrosis and negatively affects cell viability and MPS. IR inhibits MPS at dosages likely exceeding those from pharmacological exposure. OXF inhibits basal MPS at low concentrations, but the ability to respond to anabolic stimulation was present, however, less than in chemotherapy‐free conditions.

**2-04**

**Pulmonary inflammation‐induced loss and subsequent recovery of skeletal muscle mass require functional poly‐Ub conjugation**

Ramon CJ Langen, Judith JM Ceelen, Nathalie GM Thielen, Marco CJM Kelders, Chiel C de Theije and Annemie MWJ Schols

*The Department of Respiratory MedicineSchool of Nutrition and Translational Research in Metabolism (NUTRIM), Maastricht University Medical Center (MUMC), Maastricht, The Netherlands*

**Background and aims**: Exacerbations in COPD are often accompanied by pulmonary and systemic inflammation and are associated with an increased susceptibility to weight loss and muscle wasting. Both the ubiquitin (Ub)‐proteasome system (UPS) and the autophagy lysosome pathway (ALP) have been implicated in inflammation‐induced muscle atrophy. Our aim was to provide a more comprehensive overview of protein turnover regulation in skeletal muscle atrophy following pulmonary inflammation and to investigate UPS dependency of this process.

**Methods**: Pulmonary inflammation was induced in mice by an intratracheal instillation of LPS. Proteolysis (UPS and ALP) and synthesis signaling were examined in muscle homogenates. UPS dependency of muscle atrophy was addressed using Ub‐K48R mice with impaired poly‐ubiquitin conjugation.

**Results**: Administration of intratracheal LPS caused a rapid decrease in skeletal muscle mass which was attenuated in the Ub‐K48R‐ compared to control mice. Expression and activity of UPS and ALP constituents increased significantly, suggesting induction of these proteolytic pathways acutely after LPS, whereas markers for protein synthesis signaling were decreased. From 72 hours post‐LPS, control mice regained muscle weight, in line with reduced expression of UPS and ALP constituents and increased protein synthesis signaling. K48R mice however displayed impaired recovery of muscle mass.

**Conclusions**: Pulmonary inflammation‐induced muscle atrophy is only in part attributable to UPS‐mediated proteolysis, implying the contribution of ALP activation and reduced protein synthesis signaling. Changes in ALP‐ and synthesis signaling are regulated independently of poly‐Ub conjugation during muscle atrophy. Furthermore, K48 Ub conjugation is required for the recovery of muscle mass following atrophy.

**2-05**

**Role of autophagy in cancer‐related muscle wasting**

Fabio Penna ^1^, Riccardo Ballarò^1^, Paula Martinez^2^, Josep M. Argilés^3^, Paola Costelli^1^ and Antonio Zorzano^2,3,4^

^1^ *Dept. of Clinical and Biological Sciences, University of Torino, Torino, Italy;* ^2^ *Institute for Research in Biomedicine (IRB Barcelona), Barcelona, Spain;* ^3^ *Departament de Bioquímica i Biomedicina MolecularFacultat de Biologia, Universitat de Barcelona, Barcelona, Spain;* ^4^ *Centro de Investigación Biomédica en Red de Diabetes y Enfermedades Metabólicas Asociadas (CIBERDEM), Instituto de Salud Carlos III, Madrid, Spain*

**Background**: Cancer cachexia is a multifactorial syndrome characterized by anorexia, weight loss, and muscle wasting that significantly impairs patients' quality of life and survival, also reducing their tolerance to anti‐neoplastic treatments. Despite the relevance of cachexia to patient outcome, effective cachexia treatments are still lacking, and only recently tumor‐induced muscle wasting is becoming to be considered as a prognostic tool. The mechanisms underlying muscle wasting are still unclear, although the ubiquitin‐proteasome system has been involved in the degradation of bulk myofibrillar proteins. Recently, also autophagic degradation has been proposed to play a role in the onset of muscle depletion in cancer cachexia.

**Methods and Results**: The aim of this work was to test if the modulation of muscle autophagy could counteract muscle atrophy in experimental models of cancer cachexia. In order to block stress‐induced autophagy, Beclin‐1 was knocked‐down via electroporation of a specific shRNA. The results show that in sh‐Beclin‐1 electroporated tibialis of C26 carcinoma‐bearing mice, the suppression of autophagy was unable to rescue muscle fiber CSA, and unfortunately, even muscle morphology got worse. Conversely, the excessive stimulation of muscle autophagy, experimentally obtained by the overexpression of TP53INP2 (also known as DOR), exacerbated muscle atrophy in tumor‐bearing mice. At last, C26‐bearing mice were treated with formoterol, a selective β2‐agonist that protected heart and skeletal muscle against muscle atrophy. After formoterol administration, the levels of autophagy‐related proteins, p62, LC3B II, and Beclin‐1 were reduced without affecting autophagy flux, suggesting a regulation of the lysosomal‐autophagic system in response to the administration of the drug.

**Conclusions**: Overall, the results suggest that autophagic degradation plays an important role in the induction of muscle atrophy and that muscle atrophy prevention should not be pursued through direct inhibition of autophagy but through strategies that block the molecular signals upstream the activation of the lysosomal‐autophagic system.

**2-06**

***FBXL13* and *TKT* genes are associated with low pectoralis muscle area index in COPD cases**

Merry‐Lynn N. McDonald*^1^, Brian D. Hobbs^1,2^, Sungho Won^3^, Alejandro A. Diaz^2,4^, Raul San Jose Estepar^4,5^, Margaret Parker^1^, Emily Wan^1,2^, Robert Busch^1,2^, Elizabeth A. Regan^6^, Stephen I. Rennard^7^, Richard H. Casaburi^8^, Dawn L. DeMeo^1,2^, Michael H. Cho^1,2^, Craig P. Hersh^1,2^, George R. Washko^1,2^, Edwin K. Silverman^1,2^ and for the COPDGene Investigators

^1^ *Channing Division of Network Medicine;* ^2^ *Division of Pulmonary and Critical Care Medicine;* ^3^ *Department of Public Health Science, Seoul National University, South Korea;* ^4^ *Department of Radiology, Brigham and Women's Hospital, Harvard Medical School, Boston, MA;* ^5^ *Department of Pulmonary Diseases, Pontificia Universidad Católica de Chile, Santiago, Chile;* ^6^ *National Jewish Health, Denver, CO, USA;* ^7^ *Department of Medicine, Nebraska Medical Center, Omaha, Nebraska, USA;* ^8^ *Rehabilitation Clinical Trials Center, Los Angeles Biomedical Research Institute at Harbor‐UCLA Medical Center, Torrance, CA, USA*

**Background**: Cachexia is a severe complication of chronic obstructive pulmonary disease (COPD) that is likely influenced by genetic factors. However, genetic investigations in large COPD cohorts have been limited by availability of measures for monitoring muscle wasting. We investigated the genetics of a novel measure of muscle from chest CT, pectoralis muscle area index (PMI), in COPD cases using protein‐coding region genetic variants.

**Methods**: The cross‐sectional area of the pectoralis major and minor muscles were assessed in a single axial slice and divided by height squared to generate PMI. Low PMI was defined as the lowest tertile in all COPDGene subjects, a US‐based cohort of current and ex‐smokers (N = 9664). Genotyping was performed using the Illumina HumanExome array. Single nucleotide variant (SNV) and gene‐level tests were adjusted for age, sex, smoking pack‐years, and genetic ancestry. Bonferroni correction was used to define exome‐wide significance.

**Results**: COPD cases had a high prevalence of low PMI (47% overall, 63% in severe, GOLD 4 subjects). No SNV was significantly associated with low PMI. Among NHW cases (N = 2644), the *FBXL13* gene was significantly associated with low PMI (P = 8.5x10^‐7^). *FBXL13* codes for the F‐Box and Leucine‐Rich Repeat Protein 13 and is involved in the regulation of protein ubiquitination as part of an Skp, Cullin, F‐box (SCF) multi‐protein complex. Among the AA cases (N = 748), the *TKT* gene was significantly associated with low PMI (P = 1.7x10^‐6^). TKT codes for the transketolase enzyme and is necessary for the production of NADPH. Decreased transketolase activity is often associated with thiamine deficiency due to malnutrition.

**Conclusions**: We have presented evidence *FBXL13* is associated with low PMI among NHW COPD cases, and *TKT* is associated with low PMI among AA COPD cases. Future efforts will be directed at replicating the findings in other COPD populations.

**Funding**: NIH grants K99HL121087 (M.N.M.), R01HL107246 (G.R.W.), R01HL122464 (G.R.W.), R01HL089856 (E.K.S.), R01 HL089897 (E.K.S.), P01HL105339 (E.K.S.), and the Parker B. Francis Foundation Fellowship Program (M.N.M.). The COPDGene study is also supported by the COPD Foundation through contributions made to an Industry Advisory Board comprised of AstraZeneca, Boehringer Ingelheim, Novartis, GlaxoSmithKline, Pfizer, Siemens and Sunovion.

**2-07**

**Cachexia assessed by Brached‐chain amino acids and Skeleton Muscle Index in chronic stable heart failure patients**

Shuhei Tsuji and Sato Yukihito

*Department of Cardiology, Hyogo Prefectual Amagasaki General Medical Centre, Amagasaki, Hyogo, Japan*

**Background**: Sarcopenia is a common complication in severe chronic heart failure (CHF). Patients with CHF complicated with sarcopenia showed increased mortality. Branched‐chain amino acids (BCAAs) reportedly improve sarcopenia. Hence, this study aimed at identifying malnutrition in patients with CHF and identifying relationship between body composition such as skeleton muscle index (SMI), and blood samples such as brain natriuretic peptide (BNP) and amino acid concentration, especially BCAAs, in CHF patients.

**Methods**: We enrolled 220 patients who have various cardiovascular diseases and collected blood samples from them. We divided patients in two groups, one with CHF (n = 108, 62% male, aged) and without (n = 112, 66% male, aged 70.0 ∓ 9.8 years). We also selected 27 patients from CHF group (52% male, aged 74.2 ∓ 10.3 years) at random and measured their body composition using InBody720, Bioelectrical Impedance Analysis.

**Result**: Between two groups, there was no significant difference in age (71.1 ∓ 11.0 years old vs 70.0 ∓ 9.8 years old, P = 0.13) and body mass index (24.6 ∓ 17.0 kg/m^2^ vs 24.4 ∓ 3.6 kg/m^2^, P = 0.92). Hemoglobin, albumin, triglyceride, and BCAAs concentration were significantly lower in CHF group compared with non‐CHF group (12.4 ∓ 2.0 g/dl vs 13.1 ∓ 1.7 g/dl P < 0.003, 3.86 ∓ 0.38 g/dl vs 4.06 ∓ 0.36 g/dl P < 0.001, 148.3 ∓ 70.7 mg/dl vs 171.5 ∓ 80.0 mg/dl P = 0.02, 448.3 ∓ 112.9 µmol/l vs 498.8 ∓ 118.0 µmol/l P = 0.001, respectively). Average SMI was 8.6 ∓ 1.4 kg/m^2^. Patients with higher SMI was younger (P = 0.02), and had lower high‐density lipoprotein (HDL) cholesterol (P = 0.004), and BNP (P < 0.001), and higher hemoglobin (P = 0.001), triglyceride (P = 0.04), and Valine, one of BCAAs (P = 0.03).

Patients with CHF were in lower nutrition state than those without. In patients with CHF, SMI was positively correlated with Valine and paralleled with poor control of CHF represented as a high BNP concentration. Thus, HF patients may have poor nutritional status and waste skeleton muscle.

**2-08**

**mTORC1 regulation by eccentric contractions in cachectic skeletal muscle**

Justin P. Hardee ^1^, Song Gao^1^, Brandon N. VanderVeen^1^, Dennis K. Fix^1^ and James A. Carson^1,2^

^1^ *Integrative Muscle Biology Laboratory, University of South Carolina, Columbia, SC;* ^2^ *Center for Colon Cancer Research, University of South Carolina, Columbia, SC*

Eccentric contraction (ECC) induced skeletal muscle hypertrophy involves the activation of protein synthesis through mTORC1 signaling. While cancer cachexia disruption of muscle protein turnover regulation is well established, the sensitivity of cachectic muscle protein synthesis to ECCs warrants further investigation. We have established that severely cachectic muscle can initiate a growth response to repeated ECCs. However, the role of mTORC1 activation in this growth process was not established. Therefore, we examined the ECC responsiveness of cachectic muscle mTORC1 signaling and protein synthesis using 2 distinct preclinical cancer cachexia models. Male, *Apc* ^*Min*/+^ (N = 9; 16% body weight loss) and Lewis Lung Carcinoma (LLC) tumor‐bearing (N = 8; 14% body weight loss) mice performed a single bout of ECC (10 sets of 6 repetitions), and mTORC1 signaling was examined 3‐h post‐contraction. The left tibialis anterior (TA) performed ECC while the right TA served as an intra‐animal control. Age‐matched C57BL/6 (WT) mice served as controls. In control muscle *Apc* ^*Min*/+^ and LLC mice had decreased muscle mass, p70S6K (T389) phosphorylation, and protein synthesis compared to WT controls. Cachexia did not inhibit the acute ECC‐induction of p70S6K (T389) phosphorylation in *Apc* ^*Min*/+^ or LLC mice. While ECC induced protein synthesis in *Apc* ^*Min*/+^ mice, protein synthesis remained suppressed compared to WT mice. Interestingly, ECC did not stimulate muscle protein synthesis in LLC mice. Although cachexia suppressed muscle anabolic signaling, cachectic muscle maintained the ability to activate mTORC1 signaling by ECC. These results demonstrate that ECC induced responsiveness of mTORC1 signaling was maintained in cachectic muscle, but there was a disconnect between this responsiveness and the activation of muscle protein synthesis.

Supported by NIH/NCI R01‐CA121249.

**2-09**

**Skeletal muscle‐derived extracellular vesicles (EVs): an integrated approach to study the role of protein lipidation in their biogenesis and structural organization**

Valentina Buffa^1^, Daniele P. Romancino^1^, Ines Ferrara^1^, Alessandra d'Azzo^2^, Mauro Manno^3^ and Antonella Bongiovanni ^1^

^1^ *Institute of Biomedicine and Molecular Immunology (IBIM), National Research Council (CNR), Palermo, Italy;* ^2^ *Genetics Department, St. Jude Children's Research Hospital, Memphis, United States;* ^3^ *Institute of Biophysics, National Research Council (CNR), Palermo, Italy*

**Background and Aims**: Several cell types have the capacity to secrete small extracellular vesicles (EVs), as exosomes, which contain cell‐specific collections of proteins, lipids, and genetic material. Recently, we and others have shown that skeletal muscle (SkM) cell can release Alix‐positive exosomes, suggesting a new paradigm for understanding how muscles communicate with adipose tissue, the brain, or tumors. Our aims here are to understand how muscle cells generate these vesicles, to evaluate their heterogeneity and what their regulators are.

**Methods and Results**: A skeletal muscle cell line was treated or not with specific inhibitors of protein lipidation, and then the SkM‐derived exosomes were isolated using differential ultracentrifugation. To characterize exosomes and determine the role of protein lipidation (i.e., S‐palmitoylation), we applied an integrated biological/biophysical approach. We were able to determine that Alix (exosomal marker) is S‐palmitoylated and that palmitoylation inhibition altered its subcellular localization and protein interaction. We also proved that the inhibition of palmitoylation influences the number, size, heterogeneity of exosomes using dynamic light scattering (DLS), and Atomic Force Microscopy (AFM). Small‐angle X‐ray scattering (SAXS), and small‐angle neutron scattering (SANS) analyses showed that the structural organization of the lipid bilayer of palmitoylated‐inhibited exosomes is qualitatively different compared to non‐treated exosomes.

**Conclusion**: Thus, we propose that S‐palmitoylation might regulate the proper function of Alix in SkM EV biogenesis, support the interactions among the exosome‐specific regulators/biomarkers, and maintain proper EV membrane structural organization. A better understanding of EV biogenesis and function would pave a way for a possible application of SkM‐derived exosomes as a novel cell‐free based therapy for muscle degenerative diseases.

**2-10**

**Inflammatory cytokines‐induced deregulation of PI3Kγ inhibits Unacylated Ghrelin anti‐atrophic activity in skeletal muscle: implications for cancer cachexia**

Michele Ferrara^1,2^, Sara Clerici^1,2^, Elia Angelino^2^, Simone Reano^2^, Emanuela Agosti^2^, Hana Sustova^2^, Paola Costelli^3^, Emilio Hirsch^4^, Nicoletta Filigheddu^2^ and Andrea Graziani ^1,2^

^1^ *Università Vita‐Salute San Raffaele Medical School, Milano, Italy;* ^2^ *Dept. of Translational Medicine, Università del Piemonte Orientale, Novara, Italy;* ^3^ *Unit of Experimental and Clinical PathologyDepartment of Clinical and Biological Sciences, Università di Torino, Torino, Italy;* ^4^ *Unit Molecular Biotechnology CenterDepartment of Molecular Biotechnology and Health Sciences, Università di Torino, Torino, Italy*

**Background and aims**: Ghrelin is an acylated peptide hormone stimulating food intake, GH release, and positive energy balance through binding to its acylation‐selective hypothalamic receptor, GHSR1. In addition, both acylated and unacylated Ghrelin (AG and UnAG respectively) counteract muscle wasting and improve muscle function by acting directly in the skeletal muscle through a yet unidentified Gα_s_‐coupled receptor distinct from GHSR1, suggesting that Ghrelin may counteract cachexia through multiple mechanisms (1).

Gα_s_‐coupled receptors expression at cell surface is down‐regulated by the induction of PI3Kγ p101 regulatory subunit, thus making PI3Kγ independent from its negative regulation mediated by its alternative p84/87 regulatory subunit (2).

Thus, we hypothesized that the induction of PI3Kγ p101 subunit regulates UnAG responsiveness in skeletal muscle by controlling receptor density of UnAG unidentified receptor.

**Methods and Results**: We show that p101 PI3Kγ regulatory subunit is a negative regulator of UnAG anti‐atrophic activity in skeletal muscle as i) pharmacological inhibition of PI3Kγ in c2c12 myotubes enhances UnAG anti‐atrophic activity; ii) simultaneous up‐regulation of circulating UnAG and lack of PI3Kγ activity obtained by crossing Myh6/Ghrl transgenic mice with PI3Kγ kinase dead knock in mice, induces muscle hypertrophy and strongly impairs fasting‐induced muscle atrophy; iii) p101 expression is induced in cachectic skeletal muscle and positively correlates with tumor weight and muscle weight loss; iv) treatment with M1 inflammatory cytokines TNFα/IFNγ, but not with IL6, induces p101 expression in c2c12 myotubes and blunts UnAG anti‐atrophic activity; v) over‐expression of p101 in C2C12 myotubes impairs UnAG signaling.

**Conclusions**: These findings suggest the hypothesis that tumor‐induced M1 like inflammatory cytokines such as TNFα and IFNγ contribute to cancer cachexia by making the skeletal muscle resistant to ghrelin anti‐atrophic activity.

1) Porporato et al. (2013) *J*.*Clin*.*Invest*.123:611‐22

2) Perino et al. (2011) *Mol Cell*.; 42:84–95

**2-11**

**TWEAK induces oxidative stress and causes mortality in cultured mouse primary myotubes**

Dil Afroze ^1,2^, Yuji Ogura^2^ and Ashok Kumar^2,3^

^1^ *Department of Immunology and Molecular Medicine, Sher‐i‐Kashmir Institute of Medical Sciences, Srinagar, Jammu and Kashmir, India;* ^2^ *Department of Anatomical Sciences and Neurobiology, Health Science Building A, Room 1014;* ^3^ *University of Louisville School of Medicine, 500 South Preston Street, Louisville, KY, USA*

Tumor necrosis factor (TNF)‐like weak inducer of apoptosis (TWEAK) is a proinflammatory cytokine belonging to TNF super family. TWEAK produces a variety of cellular responses through binding to fibroblast growth factor inducible 14 (Fn14), a member of TNF receptor superfamily. Although Fn14 lacks a death domain, TWEAK has been found to induce apoptosis in some cell types by perturbing the activity of certain pathway such as TNF‐receptor signalling. TWEAK is also known to regulate proliferation and differentiation of myogenic cells. We have previously reported that TWEAK‐Fn14 system causes skeletal muscle wasting both in vitro and in vivo. Moreover, it has been reported that TWEAK is a mediator of atrophy in disuse conditions such as denervation. However, it remains unknown whether TWEAK can affect the viability of muscle cells. We have studied the effects of recombinant TWEAK protein on survival of cultured mouse primary myotubes. Our results demonstrate that TWEAK reduces myotube viability in a dose‐dependent manner evident by increased levels of lactate dehydrogenase (LDH) in culture supernatants. Furthermore, we have found that the levels of cleaved poly ADP ribose polymerase (PARP) and cleaved (activated) caspase‐3 are increased upon treatment with TWEAK. TWEAK also induces oxidative stress in cultured myotubes. A broad range antioxidant, N‐acetyl‐L‐cysteine (NAC), partially blocked TWEAK‐induced cytotoxicity in cultured myotubes. These results provide initial evidence that in addition to causing atrophy, TWEAK can also diminish skeletal muscle mass by inducing oxidative stress and affecting the survival of myofibers in catabolic conditions.

**2-12**

***S*‐oxprenolol delays disease progression and extends survival in a mouse model of amyotrophic lateral sclerosis (ALS)**

Cathleen Drescher ^1^, Sandra Palus^1^, Vincenzo Musolino^2^, Stefan D. Anker^1^ and Jochen Springer^1^

^1^ *Innovative Clinical TrialsDepartment of Cardiology & Pneumology, University Medical Center Göttingen (UMG), Göttingen, Germany;* ^2^ *Institute of Research for Food Safety & Health (IRC‐FSH), University of Catanzaro “Magna Graecia”, Catanzaro, Italy*

Amyotrophic lateral sclerosis (ALS) is a neurodegenerative disease that causes progressive paralysis and death to degeneration of upper and lower motoneurons in spinal cord, brainstem, and motor cortex. Cachexia is also present in ALS. The exact pathophysiology of ALS‐associated cachexia is still unknown. Currently, only riluzole is approved for the treatment of ALS. Here, we tested novel therapeutic options in an internationally standardized and established model. Using male and female transgenic G93A mice, with a mutation in the gene encoding the superoxide dismutase (SOD1), the effects of different beta blockers (10 mg/kg/d propranolol, n = 28; 20 mg/kg/d oxprenolol, n = 28; 10 or 20 mg/kg/d *R*‐oxprenolol, n = 29 and n = 30, respectively; 10 or 20 mg/kg/d, *S*‐oxprenolol n = 45 and n = 28, respectively) on survival and disease progression in comparison to riluzole as a positive control (30 mg/kg/d, n = 28) and placebo (n = 45) were tested. The disease progression assessed using neurological scores determined by international SOPs from PRIZE4LIFE and “The Jackson Laboratory.”

Survival is significantly improved at 10 and 20 mg/kg/d *R*‐oxprenolol (HR: 0.57, 95% Cl: 0.35–0.93, p = 0.0227; HR: 0.54, 95% Cl: 0.34–0.88, p = 0.013) and 20 mg/kg/d *S*‐oxprenolol (HR: 0.45, 95% Cl: 0.27–0.73, p = 0.0014) vs placebo while riluzole, propranolol, and the racemate oxprenolol had no impact in comparison to placebo. The disease progression from score 1 to score 3 of ALS was significantly attenuated by 20 mg/kg/d *S*‐oxprenolol compared to placebo (HR: 0.47, 95% Cl: 0.28–0.81, p = 0.0061). In contrast, there were no positive effects detected by treating with riluzole, propranolol, oxprenolol, and both doses of *R*‐oxprenolol as well as the lower dose of *S*‐oxprenolol.

In summary, *S*‐oxprenolol attenuates disease progression and improves survival in a G93A mouse model of ALS.

**2-13**

***S*‐oxprenolol has benefits on body weight and lean mass loss as well as on the catabolic and atrophy level in a mouse model of amyotrophic lateral sclerosis (ALS)**

Cathleen Drescher ^1^, Sandra Palus^1^, Vincenzo Musolino^2^, Stefan D. Anker^1^ and Jochen Springer^1^

^1^ *Innovative Clinical TrialsDepartment of Cardiology & Pneumology, University Medical Center Göttingen (UMG), Göttingen, Germany;* ^2^ *Institute of Research for Food Safety & Health (IRC‐FSH), University of Catanzaro “Magna Graecia”, Catanzaro, Italy*

Cachexia is a serious consequence of many diseases with symptoms like body weight loss, especially skeletal mass loss. These complications are also seen in amyotrophic lateral sclerosis (ALS). ALS is a neurodegenerative disease that affects motoneurons in brain and spinal cord, resulting in atrophy of skeletal muscles like the *M*. *gastrocnemius* (GC). This muscle wasting leads to a reduced quality of life in patients. For treatment of ALS is just one drug, named riluzole, on the market.

In this study male and female transgenic G93A mice with a mutation in the gene encoding the superoxide dismutase (SOD1) were euthanized at a defined endpoint (median survival of combined placebo groups from a survival study done before). The impact of treatment with selected beta blockers (enantiomers of oxprenolol: *R*‐oxprenolol at 20 mg/kg/d, n = 29 and *S*‐oxprenolol at 10 or 20 mg/kg/d, n = 16 and n = 26, respectively) and riluzole (30 mg/kg/d, n = 24) as well as wildtypes (n = 20) on body weight, body composition, and proteasome activity as well as atrophy level of the skeletal muscle GC compared to placebo (n = 22) were investigated. Proteasome activity analysis was measured with specific fluorogenic substrates for the chymotrypsine‐like, peptidyl‐glutamyl‐protein‐hydrolysing (PGPH) and trypsin‐like subunits of the proteasome in a flourometer. For testing the effect of the treatment on the atrophy level in muscle, the cross sectional area of the muscles fibers of GC by a hematoxylin eosin staining were determined.

Mice showed no difference in baseline body weight. Treatment with 10 mg/kg/d *S*‐oxprenolol significantly reduces body weight loss (p < 0.05). Both doses of *S*‐oxprenolol significantly attenuates loss of lean mass (p < 0.05) whereas no effect was observed for fat mass. In contrast to male mice, the proteasome activity is reduced by treatment in female mice, especially the trypsin‐like activity is significantly decreased compared to placebo (30 mg/kg/d riluzole: 1318 ± 122 nmol/mg/min; 20 mg/kg/d *R*‐oxprenolol: 1159 ± 131 nmol/mg/min; 10 mg/kg/d *S*‐oxprenolol: 1212 ± 212 nmol/mg/min; 20 mg/kg/d *S*‐oxprenolol: 1059 ± 132 nmol/mg/min vs placebo: 1744 ± 54 nmol/mg/min, respectively, p < 0.05). The cross‐sectional area of the GC fibers is improved by treating with 20 mg/kg/d *S*‐oxprenolol in comparison to placebo in female mice (20 mg/kg/d *S*‐oxprenolol: 1197 ± 175 µm^2^ vs placebo: 767 ± 96 µm^2^, p = 0.0556). In male mice the 20 mg/kg/d *R*‐oxprenolol treatment improves the size of the muscle fibers in GC (20 mg/kg/d *R*‐oxprenolol: 1181 ± 160 µm^2^ vs placebo: 899 ± 143 µm^2^).

Taken together, *S*‐oxprenolol reduces the body weight and lean mass loss. The proteasome activity is decreased, especially in treated female mice, which could have a positive impact on catabolic signaling. Interestingly, *R*‐ and *S*‐oxprenolol treatments have beneficial effects on the atrophy level in GC muscle in the ALS G93A mouse model.

**3-01**

**Selumetinib attenuate skeletal muscle wasting in murine cachexia model through ERK inhibition and AKT activation**

Yang Quan‐Jun, Huo Yan, Han Yong‐Long, Wan Li‐Li, Li Jie, Huang Jin‐Lu, Lu Jin and Guo Cheng

*Department of Pharmacy, Shanghai Jiao Tong University Affiliated Sixth People's Hospital, Shanghai, China*

Cancer cachexia is a multifactorial syndrome affecting the skeletal muscle. Previous clinical trials showed MEK inhibitor selumetinib treatment resulted in skeletal muscle anabolism. However, it is conflicting that MAPK/ERK pathway control mass of skeletal muscle. The present study investigated the therapeutical effect and mechanisms of selumetinib in amelioration of cancer cachexia. The classical cancer cachexia model was established via transplantation of CT26 colon adenocarcinoma into BALB/c mice. The effect of selumetinib on body weight, tumor growth, skeletal muscle, food intake, serum proinflammatory cytokines, E3 ligases, and MEK/ERK‐related pathways was analyzed. Two independent experiments showed that 30 mg/kg/d selumetinib prevented the loss of body weight in a murine cachexia mice. Muscle wasting was attenuated, and the expression of E3 ligases MuRF1 and Fbx32 was inhibited following selumetinib treatment of muscle gastrocnemius. Further, selumetinib efficiently reduced tumor burden without influencing the cancer cell proliferation, cumulative food intake, and serum cytokines. These results indicated that the role of selumetinib in attenuating muscle wasting was independent of cancer burden. Detailed mechanism analysis revealed AKT and mTOR were activated, while ERK, FoxO3a, and GSK3β were inhibited in selumetinib treated cachexia group. These indicated selumetinib effectively prevented skeletal muscle wasting in cancer cachexia model through ERK inhibition and AKT activation in muscle gastrocnemius via cross‐inhibition. The study not only elucidated the mechanism of MEK/ERK inhibition in skeletal muscle anabolism, but also validated selumetinib therapy as an efficacious prophylactic and therapeutic strategy against cancer cachexia.

**3-02**

**Resistance exercise training prevents cachexia‐associated muscle wasting, attenuating systemic inflammation and muscle oxidative stress in Walker‐256 tumor‐bearing rats**

Camila S. Padilha ^1^, Fernando H. Borges^2^, Lilian E.C. M Silva^3^, Fernando T. T Frajacomo^1^, Alceu Afonso Jordão^3^, José Alberto Duarte^5^, Rubens Cecchini^2^, Flávia A. Guarnier^4^ and Rafael Deminice^1^

^1^ *Department of Physical Education, State University of Londrina, Londrina, PR, Brazil;* ^2^ *Laboratory of Free Radicals and Pathophysiology State University of Londrina, Londrina, PR, Brazil;* ^3^ *Nutrition and MetabolismFaculty of Medicine of Ribeirão Preto, University of São Paulo, Ribeirão Preto, SP, Brazill;* ^4^ *Laboratory of Pathophysiology of Skeletal Muscle Adaptations, State University of Londrina, Londrina, PR, Brazil;* ^5^ *CIAFELFaculty of Sport, University of Porto, Porto, Portugal*

**Background and aims**: Cancer‐induced cachexia represents a complex metabolic disorder characterized by a progressive loss of body weight, mainly resulting from loss of skeletal muscle and adipose tissue, in a short period of time. The aim of our study was to investigate the effects of resistance exercise training (RE) on prevention of muscle waste, oxidative stress (OS), systemic inflammation, and protein synthesis/degradation balance in Walker‐256 tumor‐bearing rats. Thirty‐seven Wistar rats were divided into 4 groups: control (C,n = 9), tumor‐bearing (T,n = 10), exercised (E,n = 9), and tumor‐bearing exercised (TE,n = 10). RE protocol consisted of climbing a ladder apparatus with weights tied to the animal's tail. After 6 weeks of RE training, Walker‐256 tumor cells were implanted in the right flank. Animals of E and TE continued the RE training for more 12‐days. The physical activity of C and T rats was confined to the space of the cage.

**Results**: Tumor cells inoculation promoted reduced muscle strength (−42%), muscle wasting (P75 = −32%), increased cachexia index (9.5%), body weight loss (−93%), and decreased fat content (−45%). Increased systemic leukocytes (+153%) and systemic interleukins (TNF‐α:8‐fold, IL‐6:20‐fold, IL‐10:−39%), muscle OS (MDA:+38%, lipid hydroperoxide:+76%, and GSH/GSSG:‐61%), as well as decreased mTOR gene mRNA was also demonstrate in T group compared to C. In contrast, RE in TE group was able to increase muscle strength (+202%), attenuate muscle wasting (P75 = +58%), decrease cachexia index (5%), attenuate body weight loss (+73%), decrease fat weight (+23%). Inflammation was also mitigated as demonstrated by decreased systemic leukocytes (−38%) and interleukins TNF‐a (−45%) and IL‐6 (−80%). Exercise was also able to prevent muscle OS (MDA:−50%, lipid hydroperoxide:−52% and GSH/GSSG:+80%), but promote no changes in FBXO32 and mTOR gene mRNA.

**Conclusion**: In conclusion, RE prevents cachexia development and muscle wasting, attenuating tumor‐induced systemic pro‐inflammatory condition as well as muscle OS and oxidative damage in Walker‐256 tumor‐bearing rats.

**3-03**

**Activation of the SDF1/CXCR4 pathway retards muscle atrophy during cancer cachexia**

Giulia Benedetta Martinelli^1^, Davide Olivari^1^, Andrea David Re Cecconi^1^, Laura Talamini^1^, Linda Ottoboni^2^, Stewart H. Lecker^3^, Cynthia Stretch^4^, Vickie E. Baracos^4^, Oliver F. Bathe^5^, Andrea Resovi^6^, Raffaella Giavazzi^1^, Luigi Cervo^7^ and Rosanna Piccirillo ^1^

^1^ *Department of Oncology, Milan, Italy;* ^2^ *San Raffaele Scientific Institute, Milan, Italy;* ^3^ *Beth Israel Deaconess Center, Boston, MA, USA;* ^4^ *Department of Oncology, University of Alberta, Edmonton, Alberta, Canada;* ^5^ *Department of Surgery and Oncology, University of Calgary, Canada;* ^6^ *Department of OncologyTumor Angiogenesis Unit, IRCCS ‐ Mario Negri Institute for Pharmacological Research, Bergamo, Italy;* ^7^ *Neuroscience, IRCCS ‐ Mario Negri Institute for Pharmacological Research, Milan, Italy*

**Background and Aims**: Cancer cachexia is a life‐threatening syndrome that affects most patients with advanced cancers and causes severe body weight loss, with rapid depletion of skeletal muscle. No treatment is available. We analyzed microarray datasets to identify a subset of genes whose expression is specifically altered in cachectic muscles of Yoshida hepatoma‐bearing rodents, but not in those with diabetes, disuse, uremia or fasting.

**Methods**: In vitro: C2C12 cells were infected with adenoviruses expressing caFoxO3 or GFP and the protein synthesis and degradation rates measured.

In vivo: we induced cachexia in BALB/c mice with subcutaneous injection of colon adenocarcinoma cells C26 or in nude mice with human renal cancer cells. Tibialis anterior of mice was electroporated with SDF1‐ or CXCR4‐expressing plasmids. Nude mice received sunitinib daily for sixteen days.

**Results**: The expression of all main SDF1 isoforms (α, β, and γ) declined in Tibialis Anterior muscle from cachectic mice bearing murine colon adenocarcinoma or human renal cancer and drugs with anti‐cachexia properties (i.e. sunitinib) restored their expression. Overexpressing genes of this pathway (i.e. SDF1 or CXCR4) in cachectic muscles increased the fiber area by 20%, protecting them from wasting. Similarly, atrophying myotubes treated with either SDF1α or SDF1β had increased total protein content, resulting from reduced degradation of overall long‐lived proteins. Normal myotubes treated with the antagonist of CXCR4, AMD3100, showed a time‐ and dose‐dependent reduction in diameter, until a plateau, and lower total protein content. Notably, we found that in Rectus Abdominis muscle of cancer patients, the expression of SDF1 and CXCR4 were inversely correlated with that of two ubiquitin ligases induced in muscle wasting, atrogin‐1 and MuRF1, suggesting a possible clinical relevance of this pathway.

**Conclusion**: Our findings support the idea that activating the CXCR4 pathway in muscle suppresses the deleterious wasting associated with cancer.

**3-04**

**Oxidative stress and exercise training in experimental cancer cachexia**

Riccardo Ballarò ^1,2^, Fabrizio Pin^1,2^, Marc Beltrà^1,2^, Fabio Penna^1,2^, Mari‐Carmen Gomez‐Cabrera^3^, José Viña^3^ and Paola Costelli^1,2^

^1^ *Department of Clinical and Biological SciencesExperimental Medicine and Clinical Pathology Unit, University of Turin, Italy;* ^2^ *Interuniversity Institute of Myology, Italy;* ^3^ *Department of PhysiologyFaculty of Medicine, University of Valencia, Fundación Investigacion Hospital Clinico Universitario/INCLIVA, Valencia, Spain*

**Background and aims**: Cachexia is a multifactorial syndrome that occurs in 50 to 80% of cancer patients. It is becoming evident that oxidative stress is also involved in the pathogenesis of cachexia (1). Some years ago, moderate physical training has been proposed as a component of cachexia treatments (2) and (3) and, in physiological conditions, has been demonstrated to induce the overexpression of anti‐oxidant enzymes (4). The present study has been aimed at evaluating i) the involvement of oxidative stress in the pathogenesis of skeletal muscle atrophy; and ii) the effects of moderate exercise training on muscle wasting, with particular focus to the oxidative balance.

**Methods**: As preclinical model of cancer cachexia, Balb/c mice were subcutaneously injected with C26 colon carcinoma cells. Animals were randomized and divided into two groups, namely, controls (C, n = 11) and tumor bearers (C26, n = 16). Both controls and C26 were divided into two sub‐groups (n = 6 for C and n = 8 for C26) that were exercised through a motorized wheel.

**Results**: Exercise appeared to protect tumor‐bearing mice from reduced food intake, body weight loss, and loss of muscle mass and function. Exercise decreased the levels of carbonylated proteins compared to sedentary C26 mice. Regarding proteins involved in the antioxidant defense, exercise appeared to increase G6PD activity (while not significantly) and the levels of catalase. The Cu/Zn SOD content in tumor bearing‐mice was higher than in healthy mice without differences between sedentary and exercised groups.

**Conclusions**: Oxidative stress does not seem to be a main factor in the pathogenesis of cancer‐induced muscle wasting, at least in the experimental model used in the present study, probably because, despite being altered, muscle metabolism is still able to compensate. However, this observation does not mean that impinging on the redox balance is useless to correct muscle loss.

**References**:

1. Puig‐Vilanova et al., Free Radic. Biol. Med. 79 (2015) 91–108.
2. Penna et al., J. Cachexia Sarcopenia Muscle. (2011) 2: 95–104.
3. Penna et al., Expert Opin Investig Drugs. (2016) 25 (1):63–72.
4. Gomez‐Cabrera et al., Free Radic Biol Med. (2008) 15;44 (2):126–31.

**3-05**

**Sarcopenia is associated with hospital expenditure in patients undergoing gastrointestinal and hepatopancreatobiliary cancer surgery**

Stefan Buettner ^1†^, Jeroen L.A. van Vugt^1†^, Stef Levolger^1^, Robert R.J. Coebergh van den Braak^1^, Mustafa Suker^1^, Marcia P. Gaspersz^1^, Cornelis Verhoef^2^, Casper H.C. van Eijck^1^, Niek Bossche^3^, Bas Groot Koerkamp^1^ and Jan N.M. IJzermans^1^

^1^ *Department of Surgery, Erasmus University Medical Centre, Rotterdam, The Netherlands;* ^2^ *Department of Surgical Oncology, Erasmus MC Cancer Institute, Rotterdam, The Netherlands;* ^3^ *Department of Control and Compliance, Erasmus University Medical Centre, Rotterdam, The Netherlands*

*†Authors contributed equally to this study.*

**Introduction**: Sarcopenia has been correlated with poor postoperative outcomes, medium‐ and long‐term survival, as well as poor outcomes in specific oncologic subgroups. Furthermore, it is associated with increased health‐care cost in the United States of America. We sought to determine its effect on hospital expenditure in a Western European health‐care system, with equal access for all patients.

**Methods**: Computed tomography‐assessed skeletal muscle mass, as well as clinical and financial characteristics were obtained for 518 cancer patients who underwent abdominal cancer surgery in Erasmus University Medical Centre between 2005 and 2015. Patients were classified as (non‐)sarcopenic based on the cut‐offs established by Martin *et al*. The relationship between sarcopenia and hospital costs was assessed using linear regression analysis and Mann–Whitney U‐tests.

**Results**: Median age was 64.6 (interquartile range 57.8–71.6), and most patients were male (n = 377, 61.5%). The majority of patients had an ASA classification of 1 or 2 (n = 312, 83.4%). Most patients underwent a resection for colorectal cancer (n = 207, 33.8%), while 185 (30.2%) underwent surgery for secondary liver tumors, 160 (26.1%) for primary liver tumors, and 61 (10.0%) for pancreatic cancer. Almost half of our cohort (n = 241, 44.7%) had sarcopenia. Total hospital costs for these patients were significantly higher than for patients without sarcopenia (€16,100 vs. €11,958; p < 0.001). This effect was also observed in patients without postoperative complications (€9,926 vs. €11,737; p = 0.044). In linear regression analysis, presence of sarcopenia was associated with a cost increase of €5,392 (p = 0.001). This effect persisted after correcting for length of hospital stay and other cost‐related factors, such as cancer type and type and extent of operation.

**Conclusion**: Sarcopenia was independently associated with increased hospital costs. Reduction of sarcopenia might therefore reduce hospital costs in an era of increasing health‐care costs and an increasingly ageing population.

**3-06**

**Right ventricle loss of function, modulated by oxidative stress and calpain activity, is prevented by creatine supplementation in rats bearing Walker‐256 tumor**

Fernando H. Borges ^1^, Camila S. Padilha^2^, Poliana C. Marinello^3^, Alessandra Lourenço Cecchini^3^, Rafael Deminice^2^, Rubens Cecchini^1^ and Flávia A. Guarnier^4^

^1^ *Laboratory of Pathophysiology and Free Radicals, State University of Londrina, Paraná, Brazil;* ^2^ *Department of Physical Education‬, State University of Londrina, Paraná, Brazil;* ^3^ *Laboratory of Molecular Pathology, State University of Londrina, Paraná, Brazil;* ^4^ *Laboratory of Pathophysiology and Muscle Adaptation, State University of Londrina, Paraná, Brazil*

Heart is one of the main organs affected in cancer cachexia syndrome, and substantial heart mass loss has been reported in tumor‐bearing rats. Righ and left heart specific loss is poorly reported. The aim of this study was to investigate the effect of creatine supplementation on the right ventricle mass loss, oxidative damage and proteolytic activity after 5 and 10 days of Walker‐256 tumor implantation. Rats were divided into 5 groups: control (C), 5 (T5) and 10 (T10) days of tumor impantation (8.0 x 10^7^ cells in 0.3 mL PBS, i.m. – right side), T5 and T10 treated with creatine for 5 (TC5), and 10 (TC10) days (tumor implantation + creatine in drinking water *ad libitum* – 8 g/L). Right ventricle weight and thickness decreased 23% and 28% in T5 and T10 groups, respectively, when compared to C. Creatine partially avoided this loss at both time points. In terms of function, electrocardiogram showed increased S wave in a VL derivation (T5: −0.1; T10: −0.14 mV [p < 0.05]). Interestingly, creatine‐treated did not develop functional alterations. The specific analysis of right ventricle oxidative stress showed that both T groups showed increased levels of malondialdehyde (T5: 5.49; T10: 3.52 nmol/10 mg tissue [p < 0.001]) and carbonylated proteins (T5: 9.44; T10: 12.18 nmol/mg protein [p < 0.001]), what, in the same way of function, was prevented by creatine treatment. In addition, calpains that are markers of atrophy moodulation showed to be increased (T5: 623.4%; T10: 237.3% [p < 0.001]) when compared to C. Creatine avoided calpain‐like activity induction by tumor at both time points (TC5: −61.5%; TC10: 170.7% [p < 0.01]). We concluded that (a) tumor can promote specific right side modifications in mass and function in hearts of tumor‐bearing rats, as well as increase in oxidatie stress; and (b) creatine supplementation during tumor development prevent right ventricle mass loss probably by atenuating oxidative protein modification.

**3-07**

**Sildenafil treatment in a rat model of cancer cachexia**

Vincenzo Musolino ^1,2,3^, Cristina Carresi^1,2,3^, Micaela Gliozzi^1,2^, Caterina Giancotta^1,2^, Saverio Nucera^1,2,3^, Francesca Bosco^1,2^, Miriam Scicchitano^1,2^, Jessica Maiuolo^1,2^, Carolina Muscoli^1,2,3^ and Vincenzo Mollace^1,2,3^

^1^ *Institute of Research for Food Safety & Health (IRC‐FSH), University of Catanzaro “Magna Graecia”, Catanzaro, Italy;* ^2^ *NUTRAMED S.c.a.r.l., Roccelletta di Borgia, Catanzaro, Italy;* ^3^ *IRCCS San Raffaele Pisana, Rome, Italy*

**Introduction**: Cachexia is a complex metabolic disorder occurring in late stages of chronic disease including cancer and characterized by involuntary weight loss caused by an ongoing wasting of skeletal muscle with or without loss of adipose tissue. Cachexia also affects the cardiac muscle. As a consequence of the atrophy of the heart, cardiac function is impaired. Anti‐cachectic therapy in patients with cancer cachexia is so far limited to nutritional support. Sildenafil, a selective inhibitor of the enzyme phosphodiesterase‐5 (PDE5), has been shown to induce myocardial protective effects and to improve energy balance in a variety of experimental model. We hypothesized that sildenafil ameliorates the wasting process and the heart function in the Yoshida hepatoma tumor model.

**Study design**: In this study the effects of sildenafil were tested in cachectic tumour‐bearing rats (Yoshida AH‐130). Rats were treated daily with 30 mg/kg of sildenafil for a period of 16 days. Body weight and composition were assessed at baseline and at the end of the study. Cardiac function was analyzed by echocardiography at baseline and at day 11.

**Results**: Treatment with 30 mg/kg/d of sildenafil attenuated the loss of body weight and the wasting of fat mass. Administration of 30 mg/kg/d of sildenafil protected the heart from general atrophy. Tumor‐bearing rats displayed cardiac dysfunction, as indicated by the significant impairment of the left ventricular ejection fraction (LVEF) and the left ventricular fractional shortening (LVFS). In contrast, sildenafil improved cardiac dysfunction. Although sildenafil did not reduce the loss of lean body mass, it protects from adipose tissue depletion.

**Conclusions**: Sildenafil treatment in the Yoshida hepatoma model showed an attenuation of fat tissue loss in animals with progressive weight loss in cancer cachexia. Moreover, the drug led to an improvement of cardiac function. Larger studies with longer follow‐up and molecular analysis are required to verify these findings.

**3-08**

**Smooth muscle cell contractile phenotype and cachexia in pancreatic cancer patients: a pilot study**

Rianne D.W. Vaes ^1^, David P.J. van Dijk^1^, Linda van den Berk^1^, Liesbeth Rayen^1^, Dorit Rennspiess^2^, Axel zur Hausen^2^, Steven W.M. Olde Damink^1^ and Sander S. Rensen^1^

^1^ *Department of Surgery and NUTRIM School of Nutrition and Translational Research in Metabolism, Maastricht University, Maastricht, The Netherlands;* ^2^ *Department of Pathology and GROW School for Oncology and Developmental Biology, Maastricht University Medical Center (MUMC), Maastricht, The Netherlands*

**Background and aim**: Muscle loss in cachectic pancreatic cancer patients is most obvious in skeletal muscle, but clinical symptoms suggest that cachexia may manifest itself also in smooth muscle, a tissue that is responsible for the contraction of the gastrointestinal tract. Under pathological conditions, smooth muscle cells (SMCs) switch to a less contractile stage, which is characterized by decreased smooth muscle‐specific contractile marker proteins. To investigate whether intestinal SMC contractile phenotype is affected in cancer cachexia, we studied the abundance of several contractile SMC protein markers in the jejunum of cachectic pancreatic cancer patients.

**Methods**: We randomly selected nine jejunum tissue sections from a retrospective cohort of 133 pancreatic cancer patients who underwent surgery between 2008 and 2013 at the MUMC+. Tissue sections were immunohistochemically stained for the SMC contractile protein markers α‐smooth muscle actin (α‐SMA), SM22α, and smoothelin. Staining intensity of the two intestinal SMC layers was separately analyzed by ImageJ software. Skeletal muscle depletion (L3 muscle index) as well as skeletal muscle quality (CT muscle attenuation index (CT‐MAI)) was assessed by CT‐image analysis.

**Results**: Patient characteristics were age 71 ± 8.1 yrs, BMI 24 ± 2.9 kg/m^2^, plasma CRP 10.7 ± 10.3 mg/L, plasma albumin 42.8 ± 30.0 g/L, L3 skeletal muscle index 43.0 ± 6.7 cm^2^/m^2^, MAI 32.7 ± 15.4 HU. All SMC contractile markers were detectable in both intestinal SMC layers in all patients. SM22α staining intensity in the longitudinal intestinal smooth muscle layer was strongly correlated with CT‐MAI (ρ = 0.733, p = 0.025). Furthermore, a trend towards a significant correlation between α‐SMA staining intensity in the longitudinal smooth muscle layer and the L3 muscle index was observed (ρ = 0.567, p = 0.112). Plasma CRP and albumin levels were not associated with SMC contractile marker staining intensities.

**Conclusion**: These data suggest that cancer cachexia is not only associated with skeletal muscle wasting but also affects the contractile phenotype of intestinal smooth muscle.

**3-09**

**Effects of beta‐blockers on brown adipose tissue in a rat cancer cachexia model**

Junichi Ishida, Masakazu Saitoh, Masaaki Konishi, Stefan D. Anker and Jochen Springer

*Innovative Clinical TrialsDepartment of Cardiology and Pneumology, University Medical Centre Göttingen, Göttingen, Germany*

**Background and aims**: Cancer cachexia is a complex syndrome characterized by increased lipolysis, proteolysis, and anorexia. Previous studies indicated that chronic adrenergic stimulation increased brown adipose tissue (BAT) activity, which was partly responsible for adipose tissue loss in patients with cancer cachexia and experimental cachectic animal models. In this study, we tested the hypothesis that beta‐adrenergic blockade has anti‐thermogenic effects on BAT in a rat cachexia model.

**Methods**: 10^8^ Yoshida AH‐130 hepatoma tumor cells were intraperitoneally injected to rats, and they were randomized to receive placebo, bisoprolol 5 mg/kg/day (LBis), 50 mg/kg/day (HBis), espindolol 0.3 mg/kg/day (LEsp), or 3 mg/kg/day (HEsp). BAT was obtained on day 16 or earlier due to reaching ethical endpoints and analyzed with qPCR analysis.

**Results**: HEsp significantly improved loss of BAT and the other treatments also showed a trend towards an increased BAT weight (137 ± 64 g for HEsp, p < 0.01, 100 ± 51 g for LEsp, p = 0.83, 110 ± 64 g for HBis, p = 0.44, 119 ± 59 g for LBis, p = 0.11, vs. 88 ± 26 g for placebo). Compared with placebo group, mRNA expression levels of thermogenic genes, UCP1, and PGC‐1alpha in BAT were 1.2‐fold and 0.6‐fold with HEsp, 1.3‐fold and 1.0‐fold with LEsp, 1.1‐fold and 0.7‐fold with HBis, and 0.9‐fold and 1.5‐fold with LBis, respectively (all p = ns, vs. placebo).

**Conclusions**: High‐dose espindolol showed a preventive effect on loss of BAT, while beta‐blockers did not seem to influence gene expression involved in thermogenesis in BAT in Yoshida hepatoma model of cancer cachexia.

**3-10**

**Espindolol preserved body weight and adipose tissue and improved survival in a rat cancer cachexia model**

Junichi Ishida, Masakazu Saitoh, Masaaki Konishi, Stefan D. Anker and Jochen Springer

*Innovative Clinical TrialsDepartment of Cardiology and Pneumology, University Medical Centre Göttingen, Göttingen, Germany*

**Background and aims**: Cancer cachexia is a complex syndrome, characterized by increased lipolysis, proteolysis and anorexia. Loss of white adipose tissue (WAT), as well as skeletal muscle wasting, is associated with poor prognosis in this setting and enhanced chronic adrenergic stimulation is considered to make partial contribution to this pathological condition. In this study, we tested the hypothesis that beta‐adrenergic blockade has preventive effects on loss of body weight and WAT, resulting in better survival in a rat cachexia model.

**Methods**: 10^8^ Yoshida AH‐130 hepatoma tumor cells were intraperitoneally injected into rats, and they were randomized to receive placebo, espindolol 0.3 mg/kg/day (low‐dose), or 3 mg/kg/day (high‐dose). Body weight, body composition, and WAT were analyzed on day 16 or earlier due to reaching ethical endpoints.

**Results**: High‐dose espindolol, but not low‐dose, significantly improved survival (HR, 0.29; 95% CI, 0.16–0.51, p < 0.001 for high‐dose espindolol; HR, 0.51; 95% CI, 0.26–1.00, p = 0.051 for low‐dose espindolol, vs placebo) and preserved lean body mass (145.7 ± 10.4 g for low‐dose espindolol, 170.0 ± 13.5 g for high‐dose espindolol, vs. 118.5 ± 1.6 g for placebo) and WAT (233.7 ± 363.5 g for low‐dose espindlol, p = 0.57, 455.8 ± 488.9 g for high‐dose espindolol, p = 0.02 vs. 112.3 ± 165.9 g for placebo). In WAT of rats treated with high‐dose espindolol, expression levels of mRNA for ATGL, UCP1, and PGC‐1alpha were unexpectedly 85.3‐fold, 184.8‐fold and 7.3‐fold higher, respectively, than those of untreated rats.

**Conclusions**: Espindolol preserved body weight, lean body mass, and adipose tissue as well as improved survival in a rat cancer cachexia model. Espindolol could prevent burnout of adipose tissue in cancer cachexia.

**3-11**

**The association between sarcopenia and survival in stage I–III colorectal cancer patients**

Harm van Baar ^1^, Anne Pakkert^2^, Sandra Beijer^3^, Martijn Bours^4^, Matty Weijenberg^4^, Ellen Kampman^1^ and Renate Winkels^1^

^1^ *Division of Human Nutrition, Wageningen University, Wageningen, Netherlands;* ^2^ *VU University, Amsterdam, Netherlands;* ^3^ *Netherlands Comprehensive Cancer Organisation (IKNL), Eindhoven, Netherlands;* ^4^ *Department of Epidemiology, Maastricht University, Maastricht, Netherlands*

**Background**: It is suggested that low muscle mass (sarcopenia) at cancer diagnosis negatively affects survival in cancer patients, including colorectal cancer (CRC). However, to our knowledge, only a few small studies (i.e. <300 patients) with inconsistent results have been performed to assess the association between sarcopenia and survival in CRC patients. Also, most of these studies included metastatic (stage IV) CRC patients which may have affected the association for non‐metastatic (stage I‐III) CRC patients since the survival rate of metastatic CRC patients is considerably lower. Therefore, we aimed to investigate the association between sarcopenia and survival in stage I‐III CRC patients in a large cohort study.

**Method**: We combined data from two prospective cohort studies, the COLON and EnCoRe study, with data of the Netherlands Comprehensive Cancer Organisation. The final data set included 1,500 stage I–III CRC patients, diagnosed between 2006 and 2015. Skeletal muscle cross‐sectional area was assessed using pre‐operative computed tomography (CT) scans at the level of the 3rd lumbar vertebrae. It was normalized for height to obtain the skeletal muscle index (SMI). Sarcopenia was determined using body mass index‐ and gender‐specific SMI cut‐off points from literature. Adjusted Cox regression models were used to assess the association between sarcopenia and mortality.

**Results**: Data analysis is currently ongoing. A preliminary analysis was performed using data of 749 patients. Average age was 68.4 ± 10.3 years, 42.5% was female and 49.8% was identified as sarcopenic. The average follow‐up time was 45.3 months. No statistically significant association was found between sarcopenia and overall survival, after adjustment for age, gender, and stage of disease (HR 1.18, 95% CI: 0.87–1.61).

**Conclusion**: In the preliminary analysis, no significant association between sarcopenia and overall survival was found. Updated results will be presented during the Conference.

**4-01**

**BIO101, a drug candidate targeting Mas Receptor for the treatment of age‐related muscle degeneration. From molecular target identification to clinical development**

Pierre Dilda ^1^, Anne‐Sophie Foucault^1^, Maria Serova ^1^, Sissi On^1^, Sophie Raynal^1^, Stanislas Veillet^1^, Waly Dioh^1^ and René Lafont^1,2^

^1^ *Biophytis, Parc Biocitech, 102 avenue Gaston Roussel93230, Romainville, France;* ^2^ *Sorbonne Universités, UPMC Univ Paris 06, CNRS ‐ Institut de Biologie Paris Seine (BIOSIPE), 75005, Paris, France*

**Background**: Above 40, skeletal muscles undergo progressive loss of volume (sarcopenia) and strength (dynapenia). Reduced performances impair mobility and engage a vicious cycle where reduced physical activity results in a further muscle degradation enhanced in obese people due to the deleterious effects of muscle infiltration by adipose tissue (sarcopenic obesity).

Muscle decay results from both reduced protein synthesis and enhanced protein degradation; thus, attempts to treat sarcopenia target the muscle cells in order to improve the balance between protein synthesis and degradation processes.

**Methods**: BIO101 is a pharmaceutical grade preparation of 20‐hydroxyecdysone purified at 97% from *Stemmacantha carthamoides*. This molecule was initially investigated *in vitro* on a mouse myocytes cell line (C2C12). Markers of protein synthesis and degradation as well myotubes diameter have been determined to evaluate the effects of BIO101. The nature of BIO101 receptor and subsequent signalling pathways were studied by western blot and pull‐down assays (see presentation by Serova et al.).

BIO101 has been further tested by chronic oral administration to 12‐ and 22‐month‐old C56Bl6/J mice under hyperlipidic diet. The whole animal physical performances, the *in situ* functionality of *tibialis anterior*, and different molecular markers of selected muscles were measured before and after 4‐month treatment.

**Results**: BIO101 dose‐dependently reduced the expression of myostatin, stimulated the phosphorylation of S6K and the incorporation of ^3^H‐leucine, and increased the size of myotubes. These effects involve the activation of Mas, the angiotensin (1–7) receptor. Most importantly, we demonstrated that the treatment of old animals by BIO101 can compensate the significant loss of functionality as a consequence of aging.

**Conclusions**: Our *in vitro* and *in vivo* investigations demonstrate the BIO101 potential in improving skeletal muscle quality in ageing mammals and justify the clinical development of this drug candidate in sarcopenic patients (see presentation by Dioh et al.).

**4-02**

**Mechanism of action of BIO101, a Mas receptor activator: A drug candidate for the treatment of sarcopenia**

Maria Serova^1^, Sophie Raynal^1^, Sissi On^1^, Stanislas Veillet^1^, Waly Dioh^1^, Pierre Dilda^1^ and René Lafont^1,2^

^1^ *Biophytis, Parc Biocitech, 102 avenue Gaston Roussel, Romainville, France;* ^2^ *Sorbonne Universités, UPMC Univ Paris 06CNRS ‐ Institut de Biologie Paris Seine (BIOSIPE), Paris, France*

**Background**: Sarcopenia, a progressive age‐related reduction in skeletal muscle mass and strength which contributes to overall physical frailty, is a worldwide health challenge with limited therapeutic options. The drug candidate BIO101 is a pharmaceutical grade preparation of 20‐hydroxyecdysone purified from *Stemmacantha carthamoides*. In young animal submitted to chronic oral administration of BIO101, skeletal muscles exhibited higher protein content and significantly reduced myostatin gene expression.

**Methods**: Mouse C2C12 myoblasts were induced for differentiation, and myotube diameters were measured under fluorescent microscopy. Baseline and phosphorylated protein levels were assessed by Western blot. Relative level of mRNA expression was evaluated using qRT‐PCR. BIO101 receptor's was confirmed by pull‐down assay using 20‐hydroxyecdysone 22‐hemisuccinate‐coupled to Affi‐Gel102.

**Results**: BIO101 induced a significant enlargement of myofibers diameter (+24%, p < 0.001) and protected muscle fibers from dexamethasone induced atrophy. Pull‐down assay using C2C12 cell lysate identified Mas, the Angiotensin1‐7 receptor, as BIO101 plasma membrane target. Activation of Mas with BIO101 led to rapid (>2‐fold) increase in phospho‐AKT, phospho‐p70S6K, phospho‐ERK1/2 levels, suggesting the activation of AKT/mTOR and MAPK signaling pathways. These effects were abolished using the Mas receptor inhibitor A779. Interestingly, BIO101 also induced AMPKα and β and reduced acetyl‐CoA carboxylase activity, suggesting effects on energy homeostasis and fatty acid synthesis. BIO101 at 10 μM induced a significant decrease in myostatin gene expression (−45%; p < 0.01) similar to those elicited by 10 μM Angiotensin 1‐7 or 50 ng/ml IGF‐1. Mas receptor down‐regulation by siRNA prevented BIO101 and Angiotensin 1‐7 inhibition of myostatin gene expression.

**Conclusion**: This study demonstrates that the observed *in vitro* and *in vivo* anabolic properties of BIO101 result from an activation of Mas receptor followed by the activation of AKT/mTOR, MAPK, and AMPK pathways leading to the inhibition of myostatin gene expression. BIO101 is currently tested in a combined safety/pharmacokinetic study in human.

**4-03**

**The BET inhibitor JQ1 counteracts skeletal muscle loss during cancer cachexia and prolong survival**

Marco Segatto ^1^, Raffaella Fittipaldi^1^, Hossein Zare^2^, Roberta Sartori^3^, Claudio Fenizia^1^, Paola Costelli^4^, Marco Sandri^3^, Panagis Filippakopoulos^5^, Vittorio Sartorelli^2^ and Giuseppina Caretti^1^

^1^ *Department of Biosciences, University of Milano, Via Celoria 2620133, Milan, Italy;* ^2^ *Laboratory of Muscle Stem Cells and Gene Regulation, NIH/NIAMS, 50 South Drive, Bethesda, MD, USA;* ^3^ *Venetian Institute of Molecular Medicine, 35131, Padova, Italy;* ^4^ *Department of Clinical and Biological SciencesUnit of General and Clinical Pathology, University of Turin, Italy;* ^5^ *Structural Genomics Consortium, Old Road Campus Research BuildingNuffield Department of Medicine, Oxford, OX3 7DQ, UK*

**Background and aims**: Skeletal muscle loss is a hallmark of cancer cachexia. This multifactorial syndrome is responsible for about 25% of cancer deaths. In particular, muscle wasting in cachectic patients often leads to increased morbidity and mortality, decreased beneficial effects from chemotherapy, and poorer quality of life. Therefore, the development of therapeutic strategies aimed at preventing muscle loss during cancer cachexia is attracting increasing clinical interest. To date, no effective therapies for preventing cancer cachexia are available. Recently, we showed that the bromodomain protein BRD4 regulates pro‐atrophic genes and that the administration of the small BET inhibitor JQ1 increases muscle fiber size, protecting from dexamethasone‐induced atrophy in C2C12 myotubes. In the present study, we evaluated the effect of BRD4 inhibition by JQ1 treatment in skeletal muscle of cachectic mice.

**Methods**: C26‐tumor bearing mice were treated with JQ1 or vehicle, daily. After 12 days, body weight, skeletal muscle weight, and the anabolic/catabolic pathways involved in skeletal muscle homeostasis were evaluated. Epigenetic and transcriptional regulation of key genes involved in cancer cachexia was also analyzed by ChIP, ChIP‐seq, and RT‐PCR.

**Results**: JQ1 treatment prolongs survival by protecting tumor‐bearing mice from body weight loss, adipose tissue atrophy, and muscle wasting. In skeletal muscle, pharmacological treatment prevents muscle loss through two different mechanisms, both converging on the transcriptional regulation of muscle atrophy‐related genes: the former involves JQ1‐mediated displacement of BRD4 from atrogenes; the latter is ascribable to the suppression of IL6/AMPK/FoxO3 axis.

**Conclusions**: The obtained results suggest that the epigenetic modulation mediated by BET inhibitors is able to efficiently prevent muscle wasting and increase lifespan of tumor‐bearing mice, thus representing a promising therapeutic approach for the management of cancer cachexia.

**5-01**

**SARA‐PK: A combined study of the safety and pharmacokinetics of BIO101 in healthy young and older volunteers after single ascending and multiple ascending oral doses for 14 days**

Waly Dioh, Susanna Del Signore, Philippe Dupont, Louiza Daudigny and Stanislas Veillet

*Biophytis, Parc Biocitech, 102 avenue Gaston Roussel,93230, Romainville, France*

**Background and aims**: Ageing‐related sarcopenia, characterized by the loss of muscle mass and function, represents a key underlying cause of physical frailty, a reversible condition in older patients that often leads to mobility disability and dependency. Sarcopenic obesity (SO) is an emerging condition affecting older obese individuals and is defined by fat mass increase associated to reduced muscle mass and performance. Biophytis has developed the drug candidate Sarconeos (BIO101) acting via the renin‐angiotensin system, and specifically via the Angiotensin1‐7 receptor Mas, for the treatment of sarcopenia, including sarcopenic obesity. SARA‐PK is a randomised, double‐blind clinical trial in healthy young and older volunteers, evaluating single and multiple ascending oral doses of BIO101.

SARA‐PK objectives are to evaluate the safety, pharmacokinetics, and food effect of BIO101 administered by oral route. As an exploratory objective, the pharmacodynamic effects of BIO101 will be investigated by measuring the variations of selected biomarkers.

**Methods**: The Single Ascending Dose (SAD) consists in a staggered design where the drug BIO101 is administered to 24 subjects from two age groups: 5 cohorts of young adults (i.e., 18 ≤ age ≤55 years) at escalating doses of 100 to 1400 mg, and one cohort of older adults (i.e., 65 ≤ age ≤85 years) at one intermediate dose. In the Multiple Ascending Dose (MAD), 3 selected doses of BIO101 will be administered by oral route to 3 panels of 10 older adults (i.e., 65 ≤ age ≤85 years) over 14 days.

**Results**: Healthy volunteers' enrollment will start by the third quarter 2016 and safety and pharmacokinetics results on SAD and MAD will be presented as they become available.

**Conclusions**: SARA‐PK results will allow to define the oral doses of SARA‐INT, the interventional Phase2 trial to evaluate the efficacy and safety of Sarconeos (BIO101) for the treatment of sarcopenia, including sarcopenic obesity.

**5-02**

**Effect of docosahexaenoic acid (DHA) oral supplementation on plasma DHA levels and omega‐3 index in patients with breast cancer (BC)**

Alessio Molfino ^1^, Maria Ida Amabile^1,2^, Cesarina Ramaccini^1^, Alessio Farcomeni^3^, Sara Mazzucco^4^, Massimo Monti^2^, Gianni Biolo^4^, Filippo Rossi Fanelli^1^ and Maurizio Muscaritoli^1^

^1^ *Department of Clinical Medicine, Sapienza University of Rome, viale dell'Università 37,Rome, Italy;* ^2^ *Department of Surgical Sciences, viale Regina Margherita 324,Rome, Italy;* ^3^ *Department of Public Health and Infectious Diseases, Sapienza University of Rome, piazzale Aldo Moro 5, Rome, Italy;* ^4^ *Department of MedicalTechnological and Translational Sciences, University of Trieste, Ospedale di Cattinara, Strada di Fiume 447, Trieste, Italy*

**Background and aims**: Plasma DHA levels may affect survival in BC patients, influencing patients' nutritional and metabolic status and tumor cells sensitivity to chemo‐ and radio‐therapy. The possibility of recognizing BC patients with low DHA incorporation would help to treat this condition aimed at increasing cancer‐cell sensitivity to therapies. Aim: to ascertain whether there are differences in the incorporation of DHA in BC patients compared to controls and, specifically, differences between BC patients from families with a high aggregation of BC, sporadic BC patients and controls.

**Methods**: BC patients and healthy women were recruited. DHA (2 g/day) in the form of algal oil was administered for 10 consecutive days. Blood samples were collected at baseline (T0) and after 10 days of DHA supplementation (T1) to assess plasma DHA levels, omega‐3 index in red blood cells, and inflammatory (TNF‐α, IL‐6) markers. Parametric and non‐parametric tests were performed, as appropriate.

**Results**: Forty‐three women completed the study: 10 healthy women in group C, 10 sporadic patients in group S, 12 familiar patients in group F, and 11 BRCA1/2 gene mutation positive patients in group M. DHA and omega‐3 index increased from T0 to T1 in each group of BC patients and in controls (P < 0.001). No difference between BC patients and controls in DHA incorporation was found, except for group M (beta = 0.42, P = 0.03). DHA supplementation had no effect on inflammation. Thirty‐three participants were considered good fish consumer and 10 participants low fish consumer, whose baseline DHA levels were significantly lower with respect to the other group (P = 0.01). DHA increase from T0 to T1 was higher in low fish consumer with respect to good fish consumer (P < 0.0001).

**Conclusions**: BC patients with BRCA1/2 gene mutation showed the highest DHA incorporation. DHA supplementation increases DHA levels and omega‐3 index independently from the type of BC presentation and low fish consumers highly incorporate DHA after oral supplementation.

**5-03**

**Identification of a skeletal muscle ER‐stress‐like response to the anabolic agent, Ractopamine**

David M. Brown ^1^, Kevin JP Ryan^5^, Zoe CTR Daniel^1^, Molebeledi HD Mareko^6^, Richard Talbot^4^, Jo Moreton^3^, Tom C Giles^3^, Richard D Emes^2,3^, Thomas Charles Hodgman^1^, John M Brameld^1^ and Tim Parr^1^

^1^ *School of Biosciences, University of Nottingham, Sutton Bonington Campus, Loughborough, LE12 5RD, UK;* ^2^ *School of Veterinary Medicine and Science, University of Nottingham, Sutton Bonington Campus, Loughborough, LE12 5RD, UK;* ^3^ *Advanced Data Analysis Centre, University of Nottingham, Sutton Bonington Campus, Loughborough, LE12 5RD, UK;* ^4^ *The Roslin Institute and Royal (Dick) School of Veterinary Studies, University of Edinburgh, MidlothianEH25 9RG, UK;* ^5^ *Genetics, Guy's Hospital, London, SE1 9RT, UK;* ^6^ *Botswana College of Agriculture, Gaborone, Botswana*

**Background**: Synthetic beta‐adrenergic agonists (BA) were originally developed, and now widely prescribed, as smooth muscle bronchodilators for the treatment of asthma. At above therapeutic doses, these agents also have anabolic and anti‐catabolic effects on skeletal muscle. Despite their well‐documented efficacy to modulate muscle mass, a number of deleterious side effects have limited their application in clinical settings, such as muscle wasting associated with cachexia. An improved understanding of the biology underpinning the physiological actions of these agents would enhance further drug discovery potential in this field.

**Methods**: We administered pigs (77 ± 7 kg) with the BA, Ractopamine (20 ppm in feed), and made comparisons to a growth hormone (GH) treated and feed only control cohort. Pigs were electrically stunned and exsanguinated following 1, 3, 7, 13 (*n* = 10 per treatment, per time point), and 27 (*n* = 15 per treatment) days of treatment. We examined changes to the *Longissimus Dorsi* muscle transcriptome using quantitative RNA‐sequencing (total *n* = 45) followed by inferred pathway analysis. Gene expression changes were validated by Q‐PCR on samples from all 164 pigs. Animal work was conducted in accordance with the UK Animals (Scientific Procedures) Act of 1986 (Project License PPL 40/3010).

**Results**: Ractopamine treatment increased expression of genes involved in amino acid biosynthesis, amino acid transport, and protein translation. This response was most prominent on day 3 and diminished in magnitude thereafter. More than 20 of the BA‐responsive genes at day 3 were well‐reported transcriptional targets of the Activating Transcription Factor 4 (ATF4), strongly suggesting that an endoplasmic reticulum (ER) stress‐like response occurs within days of initiating treatment with a BA. Importantly, not all ATF4 target genes displayed increased expression with BA treatment, indicating a selective transcriptional response.

**Conclusion**: A role for this novel transcriptional ER‐stress‐like response warrants exploration in the context of regulating skeletal muscle mass, metabolism and function.

**6-01**

**Predictive value and clinical impact of anorexia, cachexia, and malnutrition on long term outcomes in patients at first medical oncology visit**

Simone Lucia^1^, Sara Emerenziani^2^, Marco Imperatori^3^, Maria P. Rescio^2^, Giuseppe Tonini^3^, Daniele Santini^1^, Filippo Rossi Fanelli^1^ and Maurizio Muscaritoli ^1^

^1^ *Department of Clinical Medicine, Sapienza University of Rome, Italy;* ^2^ *Gastroenterology Unit, Campus Bio‐Medico, Rome, Italy;* ^3^ *Oncology Unit, Campus Bio‐Medico, Rome, Italy*

**Background and aims**: Anorexia, malnutrition, and cachexia are highly prevalent in cancer patients determining up to 30% of deaths by decreasing response and tolerance to antineoplastic treatments, functional performance, reducing quality of life and survival. The present study aimed at identifying the clinical impact and the predicting value of nutritional/metabolic alterations at first medical oncology visit on clinical outcomes.

**Methods**: After informed consent, patients upon first medical oncology visit were consecutively enrolled. Inclusion criteria were diagnosis of solid tumor, age > 18 years, no previous anticancer therapies (e.g. radio‐ or chemotherapy), and life expectancy >3 months according with PaP score. All patients were assessed for anthropometric indices, anorexia (by AC/S‐12 FAACT and VAS), malnutrition (by MNA®), pre‐cachexia, and cachexia. CRP, albumin, hemoglobin, and inflammation (i.e. modified Glasgow Prognostic score, mGPS, and neutrophil/lymphocyte ratio, NLR). Evaluation for re‐hospitalization, treatment toxicity and survival was performed at 6, 24, and 30 months. ROC curves and Youden index were used to identify a predictive cut‐off value for VAS‐score. Appropriate statistical analysis was performed according to parameters’ characteristics.

**Results**: One hundred two patients (50 M:52 F;63 ± 12 years) were enrolled. Respiratory, breast, pancreas, and colorectal cancer accounted for >80% of main primitive sites mainly in advanced stages (III 25%, IV 67%). At diagnosis, 63 cancer patients (62%) had yet experienced weight loss (WL). Prevalence of anorexia, cachexia, pre‐cachexia, and malnutrition was 47%, 27%, 23%, and 3%, respectively. Gastro‐esophageal, pancreatic, and colorectal cancer showed the highest degree of WL (10, 7, and 6, respectively) that was in turn correlated with cancer site (p < 0.01) and stage (p < 0.001), anorexia (p < 0.001), inflammation (p < 0.001). Survival rates were 74%, 46%, and 44%, respectively at 6, 24, and 30 months and were predicted by malnutrition (p < 0.05), cachexia, and anorexia (p < 0.001). VAS cutoff point ≤75 showed the highest predictive value of 30‐month survival with AUC 0.80 (0.71–0.89), sensitivity 62%, specificity 86%, positive predictive value (PPV) 85%, negative predictive value (NPV) 64%, and accuracy of 72%.

**Conclusions**: Long‐term outcomes are significantly related to nutritional/metabolic alterations (i.e. anorexia, malnutrition, and cachexia). Our data strengthen the view that early, multimodal interventions aimed at maintaining/ameliorating nutritional status of cancer patients might translate into significantly improved outcomes.

**6-02**

**Relationship between undernutrition and anemia in ulcerative colitis patients**

Sergei Ivanov, Igor Khoroshilov, Evgeniy Tkachenko and Mariia Khrabrova

*North‐Western State Medical University named after I.I. Mechnikov, St. Petersburg, Russian Federation*

**Background and aim**: The undernutrition and the anemia are both frequent complications of the ulcerative colitis (UC), but impact of the undernutrition to anemia is not assessed from practical approach. Our objective is to assess relationship between of the undernutrition and the anemia in UC patients.

**Methods**: Our cross‐sectional retrospective analysis included data of 80 UC patients. Demographic characteristics, disease behavior (relapse, remission), extent of the gut damages (proctitis, left‐sided colitis, total colitis), immunosuppressive therapy, and laboratory data (levels of the haemoglobin and total serum protein) were collected. The body composition with the help of bioelectrical impedance analysis (“Diamant,” Russia) was carried out. Diagnosis of the anemia had been established according to WHO criteria. Binary logistic regression was performed to study relationship between of the undernutrition and the anemia occurrence adjusted for demographic and disease‐associated characteristics. Adjusted model includes ages of the patients, diseases activity, extent of gut damages, quantity of disease relapses, and presence of the immunosuppressive medication.

**Results**: Prevalence of the anemia in UC patients was 40%. In adjusted binary logistic model the level of total serum protein below 64 g/l and fat mass losses were associated with high frequency of the anemia occurrence: OR 5.1 (95% CI: 1.5–17.8) and 8.5 (95% CI: 1.1–63.6), respectively. There was not significant relationship between of the fat‐free mass losses and frequency of the anemia occurrence.

**Conclusions**: The undernutrition is one of the main causes of the anemia in UC patients. Findings in present study could have significant implications for treatment UC patients with undernutrition that complicated by the anemia.

**6-03**

**Increase in skimmed milk intakes brings positive calcium balances and higher serum urea nitrogen levels during body weight reduction by exercise**

Tetsuo Yamada ^1^, Shin‐ichi Kurasawa^1^, Masami Matsuzaki^1^ and Akira Tanaka^2^

^1^ *Department of Nutrition and DieteticsCollege of Nutrition, Kanto Gakuin University;* ^2^ *Laboratory of Clinical Nutrition and Medicine, Kagawa Nutrition University*

**Background and aims**: Enhancing nutrient intakes and exercise are expected for improvement in muscle wasting and bone metabolism. We investigated the effects of increasing skimmed milk intakes on protein and calcium metabolism, and nutritional status during body weight reduction by exercise.

**Methods**: Six adult male volunteers participated in two 8‐day experiments. Each experiment consisted of two 4‐day periods (total 8 days); the first half as an adjustment period and the second half as a period of body weight reduction by exercise. A wash out period was set between both of 8‐day experiments. Increasing skimmed milk intakes treatment was followed by control experiment in half of the participants, and the order was reversed for the other half (each group: n = 3). Energy intake during the adjustment period was 2,400 kcal/day (in case of 60 kg of body weight). During the second four days, the participants exercised on a bicycle ergometer (480 kcal per day, i.e. 20% of energy intake level) and consumed either control diets as same as the adjustment period, or experimental diets which contained increased skimmed milk (12.5% of energy intake level) but the same energy levels as control diets.

**Results**: Although urinary and fecal calcium excretion levels were higher, calcium balances were significantly more positive during experimental period than during control period. On the other hand, nitrogen balances tended to be more positive, and Δ serum urea nitrogen levels during the body weight reduction were significantly higher in the increased skimmed milk period than in control period. Also, larger apparent riboflavin retention based on urinary and fecal excretion levels by skimmed milk intake was observed.

**Conclusions**: Increase in skimmed milk intakes during body weight reduction by exercise brings positive calcium balances, and possibly activate protein metabolism and improve nutritional status of riboflavin.

**6-04**

**Postprandial muscle protein synthesis and muscle mass in healthy older adults is improved by supplementing breakfast with a vitamin D and leucine‐enriched whey protein medical nutrition drink**

Sjors Verlaan ^1,2^, Audrey Chanet^3,4^, Yvette C. Luiking^1^, Stéphane Walrand^3^ and Yves Boirie^3,5^

^1^ *Nutricia Research, Nutricia Advanced Medical Nutrition, Utrecht, The Netherlands;* ^2^ *Department of Internal MedicineSection of Gerontology and Geriatrics, VU University Medical Center, Amsterdam, The Netherlands;* ^3^ *INRA, UMR1019, UNH, CRNH Auvergne, Clermont‐Ferrand,, France;* ^4^ *Clermont UniversitéUniversité d'AuvergneUnité de Nutrition Humaine, Clermont‐Ferrand, France;* ^5^ *CHU Clermont‐Ferrand, Service de Nutrition Clinique, Clermont‐Ferrand, France*

**Background and aims**: A promising strategy to preserve or build muscle mass in older adults is to optimize muscle anabolism by providing an adequate amount of high quality leucine‐rich protein per meal, distributed evenly over the meals. This study aimed to investigate the acute effect of supplementing breakfast with a medical nutrition drink on postprandial muscle protein fractional synthesis rate (FSR) and to evaluate the longer‐term metabolic and muscle effects after 6 weeks of intervention in healthy older adults.

**Methods**: In a randomized, placebo‐controlled, double blind study, 24 healthy older men (71 ± 4y) consumed a vitamin D and leucine‐enriched whey protein medical nutrition drink (test group) or a placebo (control group) prior to breakfast for 6 weeks. Muscle FSR was measured at week 0 before and after intervention drink intake with standard breakfast using L‐[^2^H_5_]‐phenylalanine tracer infusion. At week 0 and week 6, dietary intake was assessed using a 3‐day dietary record and muscle mass was measured by dual‐energy x‐ray absorptiometry.

**Results**: Postprandial muscle FSR (0–4 h) was significantly higher in the test *vs*. the control group (ANCOVA, p = 0.001). During the 6 weeks intervention, the protein intake at breakfast increased from 10.1 ± 5.1 to 28.3 ± 5.0 g/day in the test group, while the intake in the control group did not change (ANCOVA, p < 0.001). The test group gained more appendicular muscle mass after 6 weeks than the control group, estimate of difference of 0.37 kg; 95%CI [0.03–0.72]; ANCOVA, p = 0.035), with a predominant gain in leg lean mass (p = 0.034).

**Conclusions**: Supplementing breakfast with a vitamin D and leucine‐enriched whey protein medical nutrition drink resulted in a higher protein intake at breakfast and a more even distribution of protein intake over the day. This nutritional strategy stimulated postprandial muscle anabolism and increased skeletal muscle mass after 6 weeks of intervention in healthy older adults.

**6-05**

**Lower postprandial glycemic response after breakfast by combined intake of a vitamin D and leucine‐enriched whey protein medical nutrition drink in healthy older adults**

Yvette C. Luiking ^1^, Audrey Chanet^2,3^, Sjors Verlaan^1,4^, Stéphane Walrand^2^ and Yves Boirie^2,5^

^1^ *Nutricia Research, Nutricia Advanced Medical Nutrition, Utrecht, The Netherlands;* ^2^ *INRA, UMR1019, UNH, CRNH Auvergne, Clermont‐Ferrand, France;* ^3^ *Clermont Université, Université d'Auvergne, Unité de Nutrition Humaine, Clermont‐Ferrand, France;* ^4^ *Department of Internal MedicineSection of Gerontology and Geriatrics, VU University Medical Center, Amsterdam, The Netherlands;* ^5^ *CHU Clermont‐Ferrand, Service de Nutrition Clinique, Clermont‐Ferrand, France*

**Background and aims**: Postprandial glycemia after breakfast or a carbohydrate load is lowered by protein intake, mainly due to a larger insulinemic response. A whey protein pre‐load before breakfast in healthy young adults, however, reduced postprandial glycemia without increasing insulin. We studied the postprandial glycemic response in healthy older adults after intake of a vitamin D and leucine‐enriched whey protein medical nutrition drink prior to breakfast.

**Methods**: In a randomized, placebo‐controlled, double‐blind, parallel‐group study, 24 healthy older men (71 ± 4y) with normal glucose tolerance consumed a medical nutrition drink (test group; 628 kJ, 21 g protein, 3 g total leucine, 3 g fat, 9 g carbohydrates, 800 IU vitamin D) or a placebo (control group) prior to breakfast for 6 weeks. Plasma glucose and insulin were measured before and during 4 h after a standard breakfast (1567 kJ, 6 g protein, 11 g fat, 60 g carbohydrates), and peak concentration (C_max_), time to peak (T_max_), and area under the curve (iAUC) were derived at week 0 and 6. The insulinogenic index (IGI) was calculated as iAUC_insulin(0‐60min)_/iAUC_glucose(0‐60min)_. Data are means ± SD.

**Results**: Both at week 0 and 6, postprandial glucose response after breakfast in the test group was lower (C_max_; 8.0 ± 1.1 vs 9.1 ± 0.9 mmol/L at week 0, *t*‐test, p = 0.018; p = 0.007 at week 6) and delayed (T_max_ 124.4 ± 54.4 vs 60.3 ± 17.3 min at week 0, *t*‐test, p = 0.002; p = 0.001 at week 6), despite higher glucose intake and similar insulin response. IGI was higher in the test group (58.7 ± 37.0 vs 19.0 ± 14.2 mU/mmol at week 0, *t*‐test, p = 0.004; p = 0.035 at week 6). C_max_ and iAUC of glucose and insulin at week 6 were not different from week 0 (MMRM, p > 0.05).

**Conclusions**: Supplementing breakfast with a vitamin D and leucine‐enriched whey protein medical nutrition drink in older adults can improve glycemic control after breakfast in the absence of an elevated insulinemic response. Glucose metabolism was not altered by 6‐week leucine‐enriched protein supplementation.

**6-06**

**Low leucine, branched chain amino acid and essential amino acid blood levels are associated with low muscle mass, strength and function**

Sovianne ter Borg ^1^, Yvette C. Luiking^1,2^, Sjors Verlaan^1^, Yves Boirie^3,4,5^, Jos M.G.A. Schols^6^ and Lisette C.P.G.M. de Groot^7^

^1^ *Nutricia Research, Nutricia Advanced Medical Nutrition, Utrecht, The Netherlands;* ^2^ *Center for Translational Research in Aging and LongevityDepartment of Health and Kinesiology, Texas A&M University, USA;* ^3^ *University of Clermont Auvergne, Unité de Nutrition Humaine, Clermont‐Ferrand, France;* ^4^ *INRA, UMR 1019, UNH, CRNH Auvergne, Clermont‐Ferrand, France;* ^5^ *CHU Clermont‐Ferrand, Clinical Nutrition Department, Clermont‐Ferrand, France;* ^6^ *Department of Health Services Research and Department of Family MedicineSchool CAPHRI, Maastricht University, Maastricht, The Netherlands;* ^7^ *Wageningen University, Division of Human Nutrition, Wageningen, The Netherlands*

**Background**: Nutritional recommendations for the management of sarcopenia are based on adequate intake of dietary protein, including sufficient amount of essential amino acids (EAA) especially leucine. Although studies indicate a relation between higher protein and leucine intake and lower muscle mass loss, little observational data are available on the association between blood amino acid levels and muscle parameters in older adults.

**Methods**: The Maastricht Sarcopenia Study included 227 community‐dwelling older adults (74.9 ± 7.2y; mean ± SD). Postabsorptive serum amino acid levels were analysed with Ultra Fast Liquid Chromatography. Muscle mass (skeletal muscle index: SMI), strength, and function were measured by bio‐electrical impedance, handheld‐dynamometer, 5‐times chair stand, and 4‐meter gait speed. Amino acid level quartiles (Q) were compared by ANOVA and Tukey's post‐hoc comparison. Data are presented as mean ± SD.

**Results**: Participants with the lowest essential amino acids (EAA) levels (Q1: <1020 µmol/L) compared to the highest levels (Q4: >1232 µmol/L) had a lower SMI (7.6 ± 1.5 vs 8.4 ± 1.7 kg/m^2^, P < 0.001), handgrip strength (21.2 ± 8.5 vs 28.9 ± 9.4 kg, P < 0.001), and gait speed (0.9 ± 0.3 vs 1.1 ± 0.3 m/s, P = 0.013). Moreover, time to complete the chair stand test was longer in Q1 vs Q4 for EAA (15.0 ± 6.2 vs 12.3 ± 2.8 s, P = 0.009). Participants with the lowest leucine levels (Q1: <137 µmol/L) compared to the highest levels (Q4: >178 µmol/L) had lower SMI (7.4 ± 1.5 vs 8.8 ± 1.6 kg/m^2^, P < 0.001) and handgrip strength (21.1 ± 8.4 vs 30.9 ± 9.2 kg, P < 0.001), and a longer chair stand time (14.7 ± 6.2 vs 12.1 ± 8.4 s, P = 0.026). Similar results were obtained for total BCAA. No significant differences were observed for total amino acids.

**Conclusions**: Low blood levels of leucine, BCAA and EAA are associated with lower muscle mass, strength and increased chair stand time in older adults. In addition, low levels of EAA are associated with low gait speed. Increasing the dietary intake of these amino acids may contribute to improving muscle parameters in older adults.

**6-07**

**Aged mice require leucine in the context of a protein meal to induce an anabolic response**

Francina J. Dijk ^1^, Miriam van Dijk^1^, Stéphane Walrand^2^, Luc JC van Loon^3^, Klaske van Norren^4^ and Yvette Luiking^1,5^

^1^ *Nutricia Research, Nutricia Advanced Medicial Nutrition, Utrecht, The Netherlands;* ^2^ *Unite de Nutrition Humaine, INRA‐UdA, Clermont‐Ferrand, France;* ^3^ *NUTRIM School of Nutrition and Translational Research in Metabolism, Maastricht University Medical Centre, Maastricht, The Netherlands;* ^4^ *Nutrition and Pharmacology GroupDivision of Human Nutrition, Wageningen University, Wageningen, The Netherlands;* ^5^ *Center for Translational Research in Aging and LongevityDepartment of Health and Kinesiology, Texas A&M University, USA*

**Background and aims**: Anabolic resistance has been suggested to contribute to the failure of muscle maintenance in the elderly. Of all amino acids, leucine is the most prominent anabolic stimulus of skeletal muscle. Our aim was to assess the anabolic response after a bolus of free leucine or leucine combined with whey‐protein on muscle protein synthesis (MPS) in aged mice.

**Methods**: Overnight fasted 25‐mo old C57/BL6RJ‐mice received an oral gavage with leucine (LEU, 0.75 g/kg bw), leucine‐enriched whey‐protein (WHEY + LEU, 0.75 g/kg bw total leucine), or 0.5 mL water (fasted CONTROL). Subsequently, after 30, 60, or 90 min, mice were s.c. injected with puromycin (0.04 µmol/g bw) and sacrificed 30 min thereafter. Leucine and essential amino acid (EAA) concentrations were determined in plasma and *tibialis anterior* (TA) muscle. MPS was measured in TA by the SUnSET method. Total and phosphorylated proteins of p70S6 kinase, 4EBP1, and Akt were measured by Western blotting.

**Results**: Plasma and muscle free leucine concentrations increased about 6‐fold and 4‐fold, respectively, at 60 min (P < 0.05 vs CONTROL), not different between groups. Plasma leucine increased further after LEU (P < 0.05 at 90 min vs 60 min and vs WHEY + LEU). While WHEY + LEU increased free EAA concentration in TA (P < 0.05 vs CONTROL), EAA remained unchanged after LEU. LEU did not change MPS after 60 min, however, after 90 min MPS decreased to 58% of CONTROL (P < 0.05). In contrast, WHEY + LEU stimulated MPS up to 165% at 60 min (P < 0.05 vs CONTROL). Phospho/total ratios of p70S6k and 4EBP1 showed an increase after WHEY + LEU (P < 0.05 vs CONTROL), with no changes observed after LEU. No significant changes were observed for Akt‐phosphorylation.

**Conclusions**: MPS is stimulated in aged mice by an oral bolus of leucine‐enriched whey‐protein, but not by administration of leucine only. Aged mice probably require leucine in the context of a protein meal with adequate availability of other EAA to induce a muscle protein synthetic response.

**6-08**

**Leucine‐rich diet improves liver metabolism, maintaining energy store and reducing gluconeogenesis enzyme expression in Walker 256 tumour‐bearing rats (experimental model of cancer cachexia)**

Laís Rosa Viana, Anna Caroline Perina Luiz, Carla de Moraes Salgado, Emilianne Miguel Salomão, Bianca Cristine Favero and Maria Cristina Cintra Gomes‐Marcondes

*Laboratory of Nutrition and CancerDepartment of Structural and Functional Biology, University of Campinas, São Paulo, Brazil*

**Background and Aims**: Liver tissue may contribute to the hypermetabolic state usually found in cancer cachexia hosts. The present study evaluated the hepatic metabolism in Walker 256 tumour‐bearing rats under the modulation effects of leucine‐rich diet, largely used to prevent skeletal muscle loss.

**Methods**: Wistar rats were randomly distributed into 4 groups: control (**C**) and tumour‐bearing (**W**), both groups fed a control diet (18% protein), and leucine (**L**) and leucine tumour‐bearing (**LW**) groups fed a leucine‐rich diet (18% protein + 3% L‐leucine). After 21 days of tumour evolution, liver tissue was collected to access the histological Periodic acid Schiff (PAS) stain, real‐time PCR, and Western blotting analyses.

**Results**: Hepatic glycogen content reduced in W, but was preserved in LW group (no difference was found between LW and L groups). Phosphoenolpyruvate carboxykinase (PEPCK) gene expression was increased in W group (P = 0.0004), but unchanged in LW compared to control groups. Also, the AMP‐activated protein kinase (AMPK) activation was higher in W than in C group, but in LW this kinase reduced in comparison to W group. In parallel, serum lactate content decreased in LW compared to W group. Additionally, the cachexia index was lower in LW than in W group.

**Conclusions**: Our results suggest that leucine‐rich diet may decrease the hypermetabolism state and consequently reducing the host tissues waste, maintaining the energy stores and likely decreasing the futile (Cori) cycle in the liver.

**6-09**

**Patterns of protein‐to‐calorie intake in minority hemodialysis patients**

Amy S. You, Amanda Brown, Kavanaugh Kaji, Tracy Nakata, Elani Streja, Lidia Lou, Mary Veliz, Kamyar Kalantar‐Zadeh and Connie M. Rhee

*Division of Nephrology, University of California Irvine, Orange, CA, USA*

**Background and aims**: Given that heightened catabolism and dialytic amino acid/protein losses, National Kidney Foundation‐Kidney Disease Outcomes Quality Initiatives Nutrition Guidelines recommend that hemodialysis patients consume a higher protein intake of 1.2 g/kg and 30–35 kcal/kg of body weight per day. However, little is known about the proportion of caloric intake from dietary protein sources in this population, particularly among minorities.

**Methods**: Among 62 hemodialysis patients from the prospective “Malnutrition, Diet, and Racial Disparities in Chronic Kidney Disease” study, we examined the distribution of protein‐to‐caloric intake according to racial/ethnic groups. Information on patients’ protein and caloric intake was obtained using the Block Food Frequency Questionnaire administered over the period of October 2011‐March 2015.

**Results**: The mean ± SD age of the cohort was 52 ± 13 years, among whom 45% were women, and 48%, 26%, and 21% were Hispanic, African‐American, and Asian, respectively. When comparing the proportion of caloric intake from dietary protein across racial/ethnicity, we found that Hispanics and African‐Americans had the lower proportion of caloric intake due to protein sources, whereas Asians had the highest proportion of intake: median (IQR) 16% (13%, 18%), 16% (12%, 21%), and 20% (17%, 25%), respectively (Figure). However, African–Americans had the highest absolute intake of protein across racial/ethnic groups: median (IQR) 41.9 (31.3, 62.5), 67.1 (34.3, 99.0), and 41.1 (23.0, 69.1) for Hispanics, African–Americans, and Asians, respectively. African–Americans also had the highest absolute caloric intake: median (IQR) 989 (718, 1531), 1528 (745, 1876), and 933 (562, 1344) kcal for Hispanics, African–Americans, and Asians, respectively.

**Conclusion**: Across minority hemodialysis patients, Asians had the highest proportion of caloric intake due to dietary protein. However, African‐Americans had the highest absolute protein and caloric intake across racial/ethnic groups. Further studies are needed to determine how differences in dietary composition of minority hemodialysis patients influences outcomes in this population.